# Photoinduced Cascade Reactions of 2-Allylphenol Derivatives toward the Production of 2,3-Dihydrobenzofurans

**DOI:** 10.1021/acs.joc.3c00347

**Published:** 2023-03-31

**Authors:** Vasco Corti, Jacopo Dosso, Maurizio Prato, Giacomo Filippini

**Affiliations:** † Department of Chemical and Pharmaceutical Sciences, Center of Excellence for Nanostructured Materials (CENMAT), INSTM − UdR Trieste, University of Trieste, 34127 Trieste, Italy; ‡ Center for Cooperative Research in Biomaterials (CIC biomaGUNE), Basque Research and Technology Alliance (BRTA), 20014 Donostia, San Sebastián, Spain; § Basque Foundation for Science, Ikerbasque, 48013 Bilbao, Spain

## Abstract

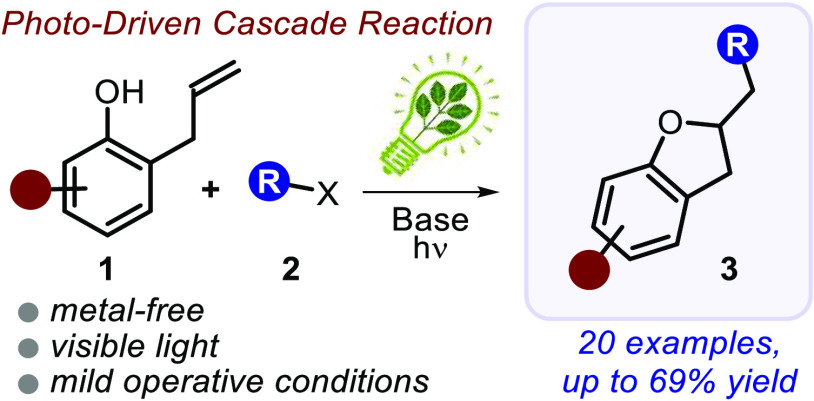

A light-driven protocol for the synthesis of 2,3-dihydrobenzofurans under mild conditions is reported. Specifically, the cascade process is initiated by the photochemical activity of allyl-functionalized phenolate anions, generated *in situ* upon deprotonation of the corresponding phenols. The reaction proceeds rapidly with reaction times as low as 35 min, delivering a wide range of densely functionalized products (20 examples, yields up to 69%). Mechanistic studies have also been performed providing convincing evidence for the photochemical formation of carbon-centered radical species. A cascade reaction pathway involving a tandem atom transfer radical addition (ATRA) and an intramolecular nucleophilic substitution (SN) process is proposed to occur.

## Introduction

**Figure 1:**
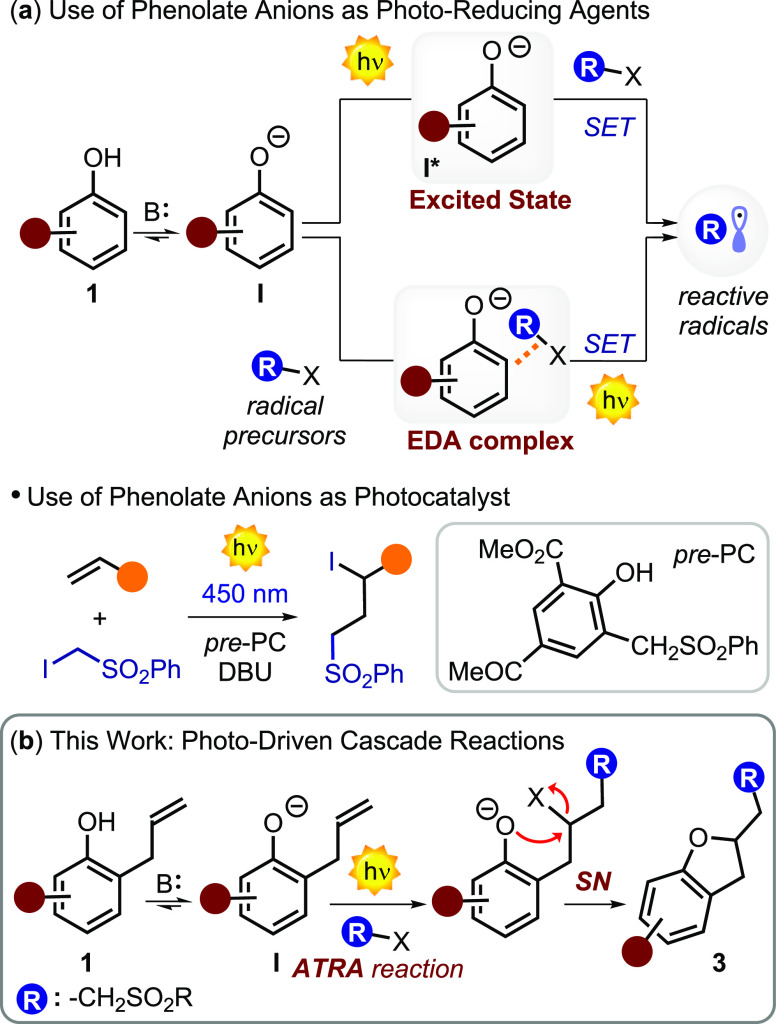
(a) Exploitation of phenolate anions **I** for the photochemical formation of reactive radicals from suitable radical precursors. (b) This work: use of visible light to drive the transformation of 2-allylphenol derivatives **1** into 2,3-dihydrobenzofurans **3**. B: base; X: halogen atom; SET: single electron transfer; ATRA: atom transfer radical addition; DBU: 1,8-diazabicyclo[5.4.0]undec-7-ene.

Phenols **1** are ubiquitous chemical functionalities that play key roles in many natural, synthetic, and industrial processes.^1^ Indeed, these aromatic moieties are widely present in numerous natural compounds (e.g., hormones, amino acids, vitamins, and neurotransmitters), active drugs, functional materials, biopolymers (such as lignin), among others.^2−5^ Consequently, in recent years, organic chemists have taken a resolute step toward the development of new effective synthetic methodologies that allow for the selective functionalization of the phenolic scaffold.^6^ Specifically, phenols and their conjugate bases, namely, phenolate anions **I** (Figure 1), are electron-rich aromatic species that show a strong nucleophilic character. Indeed, typical derivatization protocols of phenols rely on classical organic transformations, such as (i) Friedel–Craft alkylation and acylation, (ii) nitration and nitrosation, (iii) electrophilic halogenation, (iv) metal-catalyzed C–H functionalization, among others.^6^ In addition, phenolates are active organic chromophores that may absorb light within the visible region when functionalized with electron-withdrawing groups (EWGs).^7^ In particular, phenolates **I** become strong reductants in the excited state capable of generating reactive radicals from suitable precursors via single electron transfer (SET) processes (Figure 1a).^8^ Hence, these anions may be employed to photochemically trigger strategic bond-forming reactions, including their direct aromatic C–H functionalization, avoiding the utilization of an external photoredox catalyst.^9a−9c^ The ability of electronically excited phenolate anions I* (Figure 1a) to produce reactive open-shell species was first described in 2015. Specifically, it was reported as a direct strategy to install fluoroalkyl groups on the phenyl rings of phenol derivatives.^9c^ Interestingly, suitable phenolate anions may be also employed as photo-organocatalysts to drive the synthesis of relevant molecules.^7,10a,10b^ As examples, Shang and co-workers have described the use of *o*-phosphinophenolates as photocatalysts for the defluoroalkylation and hydrodefluorination of trifluoromethyl groups and for the borylation of aryl halides.^11a,11b^ Recently, employing a similar approach, our group developed a novel phenolate-based photocatalytic system capable of driving the production of valuable alkyl iodides (Figure 1a).^10c^ An alternative mechanistic manifold is represented by the ability of phenolate derivatives to form electron donor–acceptor (EDA) complexes with electron-poor radical precursors (Figure 1a).^7,9d,12^ An intriguing aspect of these ground-state molecular aggregates is that, generally, their absorption profiles show a bathochromic shift.^13^ Thus, when the EDA complex is irradiated with light of an appropriate wavelength, an electron transfer can occur, resulting in the formation of reactive radicals that can be used to initiate organic transformations.^14^ As an example, this strategy was used by Guo et al. to develop light-promoted dearomative fluoroalkylation of β-naphthols.^12a^ In addition, in 2022, our group found that **I** and α-iodo sulfones can form EDA complexes through halogen-bond interactions, which are capable of photochemically trigger alkylation reactions of **1**.^9d^ Here, we report a cascade reaction that merges the excited-state and ground-state reactivity of phenolate anions. This strategy converts 2-allylphenol derivatives **1** and suitable radical precursors, such as α-iodo sulfones **2**, into synthetically valuable sulfone-containing 2,3-dihydrobenzofurans **3** in a single strike (Figure 1b). Importantly, the 2,3-dihydrobenzofuran core is widely present in natural compounds and biologically active drugs.^15^ This class of products displays various biological activities, such as anti-HIV, antimalarial, anticancer, antinociceptive, anti-inflammatory, antifungal, and antibacterial activities.^15,16^

Available synthetic strategies for the construction of the 2,3-dihydrobenzofuran scaffold generally rely on thermal methods, such as (i) rearrangement reactions of chromanones, (ii) *O*-arylation of suitable alcohols, (iii) hydrogenation of the C_2_–C_3_ double bonds of benzofurans, (iv) cycloaddition reactions of alkyne-containing ether derivatives, (v) metal-catalyzed C–H functionalization reactions, among others.^15^ These approaches typically employ harsh operative conditions, such as the use of high reaction temperatures and transition-metal-based catalytic systems, which may be expensive and potentially toxic. To overcome these problems, in recent years, organic photochemistry has become a prominent tool to guide the development of greener and more sustainable synthetic protocols. Despite this progress, photochemical protocols which allow the direct production of 2,3-dihydrobenzofuran remain rare.^16^ Remarkably, our two-step process, which is initiated by the photochemical activity of phenolates **I**, involves an initial atom transfer radical addition (ATRA) reaction followed by a nucleophilic substitution (SN) to afford products **3**.

## Results and Discussion

**Figure 2:**
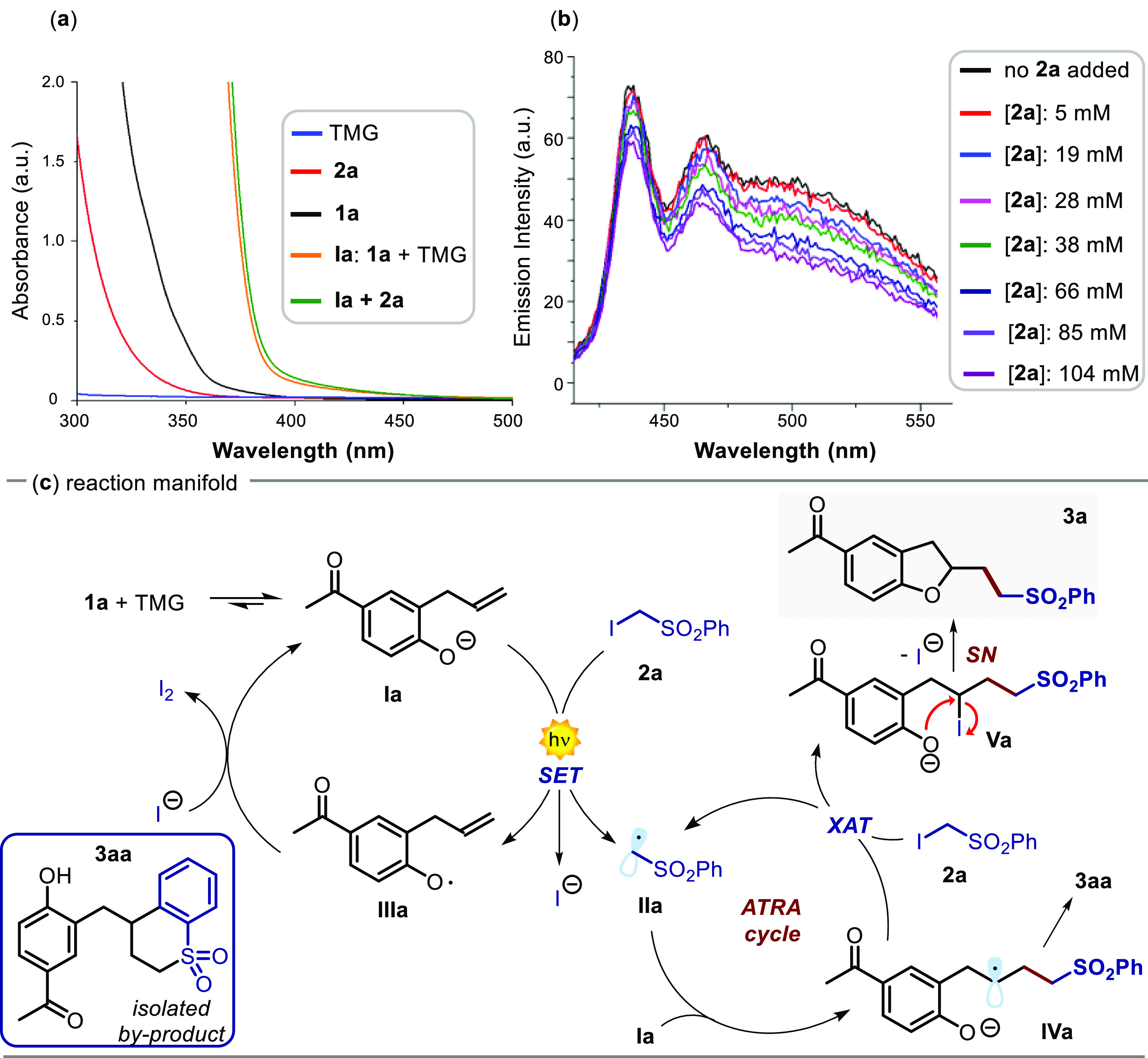
(a) Optical absorption spectra recorded in 1,2-dichlorobenzene: [**1a**] = 0.01 M (black line); [**2a**] = 0.01 M (red line); [TMG] = 0.01 M (blue line). (b) Quenching of the phenolate **Ia** emission ([**Ia**] = 0.015 M in 1,2-dichlorobenzene, excitation at 400 nm) in the presence of increasing amounts of **2a**. (c) Mechanism of the photoinduced cascade reaction. XAT: halogen-atom transfer.

We started our studies by reacting 4-acetyl-2-allylphenol **1a** and α-iodo sulfone **2a** (Table 1). The experiments were carried out at ambient temperature in acetonitrile and under visible light irradiation using a Kessil lamp at 456 nm. Importantly, when adding the base—namely, 1,1,3,3-tetramethylguanidine (TMG)—the desired dihydrobenzofuran **3a** was formed in moderate chemical yield (entry 1, Table 1). In order to gain more mechanistic insights, we carried out a series of control experiments. Excluding the light source resulted in the suppression of the process, therefore establishing the photochemical nature of the transformation (entry 2, Table 1). The presence of air in the reaction vessel prevented the formation of the desired product, probably indicating that a radical mechanism is operating (entry 3, Table 1). In addition, performing the reaction in the absence of TMG resulted in no reaction (entry 4, Table 1). This result highlights that phenolate **Ia**, generated *in situ* from **1a**, was essential for carrying out this transformation. Indeed, upon addition of TMG, the solution of **1a**, which was almost colorless, promptly intensified its yellow coloration, indicating the ability of the anion **Ia** to absorb visible light (yellow line in Figure 2a). Addition of the radical precursor **2a** resulted in subtle changes of the absorption spectrum (green line in Figure 2a). This result suggests that the formation of an EDA complex between **Ia** and **2a** might not be at the roots of the observed reactivity.^17^ Therefore, from a mechanistic point of view, the reaction is probably triggered by the photoredox properties of phenolate **Ia** (Figure 2c). In fact, upon light absorption, **Ia** can directly reach an electronically excited state Ia* becoming a strong reducing agent, as indicated by its reduction potential, which was estimated to be −2.87 V (vs SCE).

**Table 1:**
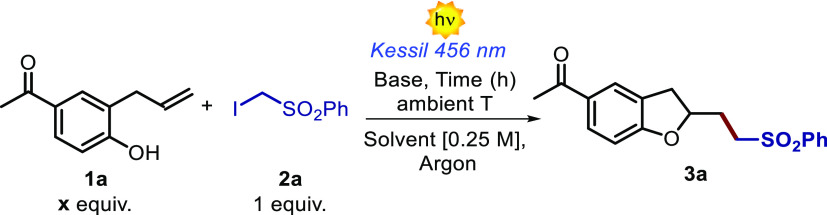
Optimization Studies and Control Experiments^a^

Entry	x	Time	Solvent	Base (equiv.)	Deviations	Yield **3a** (%)
1	3	18 h	Acetonitrile	TMG (3)	-	40
2	3	18 h	Acetonitrile	TMG (3)	*in the dark*	<5
3	3	18 h	Acetonitrile	TMG (3)	*in the air*	<5
4	3	18 h	Acetonitrile	-	-	<5
5	1.5	35 min	1,2-Cl_2_-C_6_H_4_	TMG (1.5)	-	65

a Reactions were performed on a 0.10 mmol scale. Yields were determined by ^1^H NMR analysis using 1,1,2-trichloroethene as the internal standard.

**Scheme 1:**
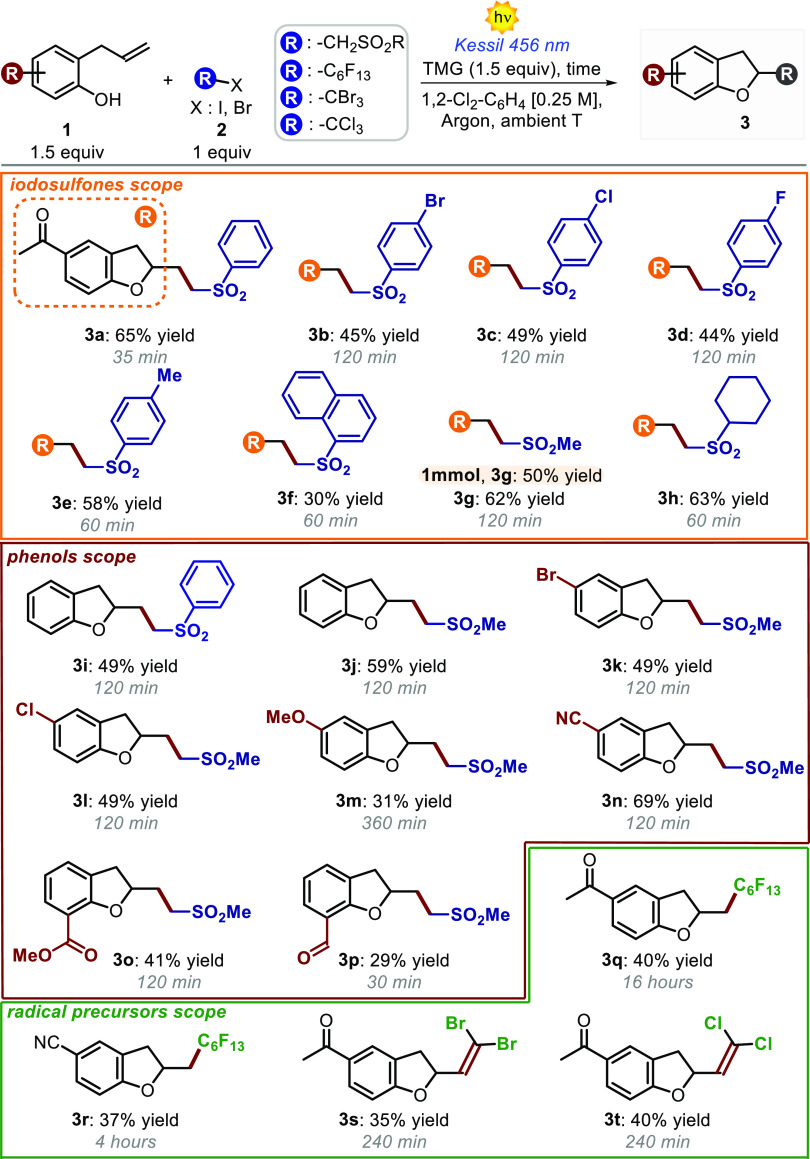
Scope of the 2-Allylphenols **1** and the Radical Precursors **2** That May Participate in the Photoinduced Cascade Process Reactions were performed on a 0.15 mmol scale using 1.5 equiv of **1**.

Thus, Ia* can trigger the generation of an electron-deficient radical **IIa** through the reductive cleavage of the C–I bond within **2a** (*E*_red_ = −1.4 V vs SCE) via a single electron transfer (SET) mechanism.^18^ To corroborate this hypothesis, we have recorded the emission spectra of **Ia** upon excitation at 400 nm (Figure 2b, maximum emission at 465 nm). Stern–Volmer quenching experiments were performed, which showed that the radical precursor **2a** effectively quenched the excited state of **Ia**. In our studies, a linear Stern–Volmer correlation is observed, meaning that a single type of quenching phenomenon occurs, likely via a SET mechanism (see Figure S5).^17^ After the photochemical initiation step, the radical **IIa** reacts with the alkene fragment of **Ia**, possibly entering an ATRA chain cycle to yield **Va**.^10c^ This intermediate undergoes an intramolecular SN reaction forming the final product **3a**. Additionally, we were able to isolate in low yield (less than 10%) the main byproduct of the process, namely, **3aa**, under the conditions depicted in entry 1 of Table 1 (blue box in Figure 2c). Likely, this bicyclic compound arises from the intramolecular cyclization reaction between the C-centered radical and the phenyl ring of the sulfone moiety of **IVa**. Further optimizations (see the Supporting Information) revealed that using 1,2-dichlorobenzene (1,2-Cl_2_-C_6_H_4_) as solvent along with a slight excess of **1a** (1.5 equiv) led to the formation of **3a** in good yield (65%) after only 35 min overall reaction time (entry 5, Table 1). Afterward, using the optimized reaction conditions, we explored the generality of the reaction with respect to the α-iodo sulfone component (Scheme 1). We successfully employed both aryl- and alkyl-substituted α-iodo sulfones as radical precursors (products **3a**–**3h**). In all cases, we registered moderate to good chemical yields (up to 65%). On the other hand, the reaction efficiently tolerates various phenol derivatives bearing halide, ether, cyano, ester, and aldehyde moieties (products **3i**–**3p**). The photochemical transformation is amenable to scale-up (1 mmol, product **3g**) with only a poor erosion of the chemical yield (50% yield). We then evaluated the possibility to apply our strategy to other easily reducible alkyl halides, such as perfluorohexyl iodide **2i**. Interestingly, we isolated products **3q** and **3r** in moderate chemical yields. We found that **Ia** and perfluorohexyl iodide may actively form a photoactive EDA complex when mixed in solution and that this aggregate is capable of initiating the photodriven cascade reaction (see Figure S3). Interestingly, the dual-reactivity profile of phenolate anions **I**, which are able to act both as photoreducing agents and donors in EDA complexes formation, allowed the development of a more general approach under very mild reaction conditions. In addition, also tetrabromomethane **2j** and bromo(trichloro)methane **2k** were suitable substrates for this transformation. Surprisingly, the use of these precursors led to the production of compounds **3s** and **3t**, which bear a *gem*-dibromoalkene and a *gem*-dichloroalkene fragment, respectively.

**Scheme 2:**
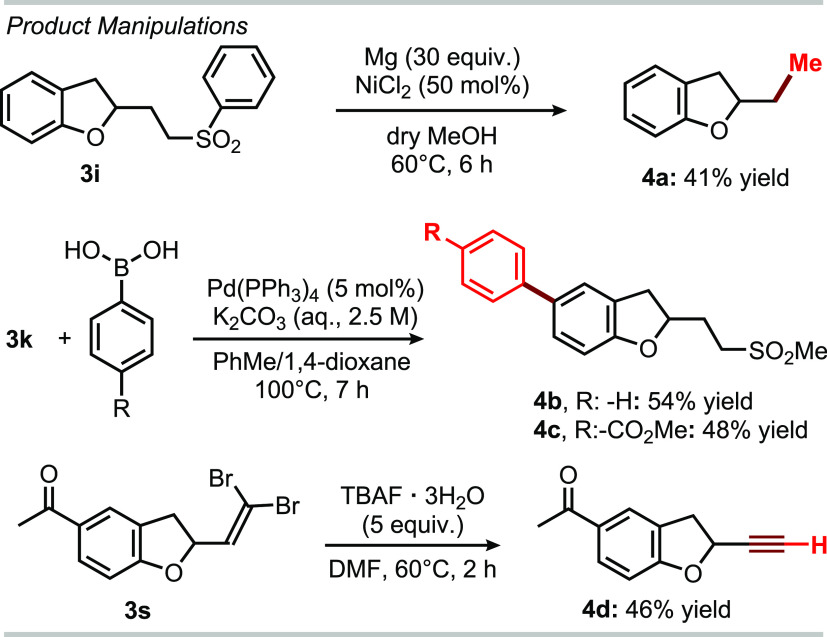
Manipulations of Products **3** TBAF: tetrabutylammonium fluoride.

These products are probably the result of an additional elimination step of 1 equiv of HX that may take place under basic conditions. Also in these cases, **Ia** and the electron-deficient species **2j** and **2k** can form EDA complexes that are responsible for the observed reactivity (see Figures S1 and S2). To further demonstrate the synthetic potential of the developed photochemical cascade process, we decided to carry out manipulation reactions on the obtained products **3** (Scheme 2). Desulfonylation of **3i** was easily achieved under reducing conditions (Mg in dry MeOH) to afford the ethyl group and the desired adduct **4a**.^18,19^ Moreover, the bromide atom of **3k** was used to increase the molecular complexity, hence providing products **4b** and **4c** (54 and 48% yields, respectively) through Pd-catalyzed Suzuki cross-coupling reactions. Lastly, compound **3s** was effectively transformed into the corresponding alkyne-containing derivative, namely, **4d**. These experiments demonstrate the relevance of compounds **3**, which may be effectively employed as synthetic building blocks to access relevant molecular architectures.

## Conclusions

In conclusion, we have developed a new metal-free photochemical cascade reaction that enables the direct conversion of 2-allylphenol derivatives **1** and easily reducible alkyl halides **2** into synthetically valuable 2,3-dihydrobenzofurans **3**, under mild reaction conditions. These transformations are initiated by the photochemical activity of phenolate anions **I**, produced *in situ* upon deprotonation of **1**, that can either directly photoreduce the radical precursors or form photoactive EDA complexes with these electron-deficient species. Importantly, this photochemical transformation provides a wide variety of functionalized 2,3-dihydrobenzofurans **3** (20 examples, up to 69% yield). Lastly, the synthetic potential of this approach was demonstrated by scaling up the process (up to 1 mmol) while accessing a series of relevant product manipulations.

## Experimental Section

### General Information

NMR spectra were recorded on a Bruker 400 Avance III HD equipped with a BBI-z grad probe head 5mm and a Bruker 500 Avance III equipped with a BBI-ATM-z grad probe head 5mm (^1^H: 400 MHz, ^13^C: 100.5 MHz, ^19^F: 376 MHz, ^1^H: 500 MHz, ^13^C: 125 MHz). The chemical shifts (δ) for ^1^H and ^13^C are given in ppm relative to residual signals of the solvents (CHCl_3_ @ 7.26 ppm for ^1^H NMR, and @ 77.16 ppm for ^13^C NMR; CFCl_3_ @ 0.0 ppm for ^19^F NMR spectra). Coupling constants are given in hertz. The following abbreviations are used to indicate the multiplicity: s, singlet; d, doublet; t, triplet; q, quartet; m, multiplet; br, broad signal. NMR yields were calculated by using trichloroethylene as internal standard. Microwave synthesis was performed on a CEM Discover-SP, using 10 mL glass microwave tubes. High-resolution mass spectra (HRMS) were obtained using a Bruker micrOTOF-Q (ESI-TOF). Absorption spectroscopy studies have been performed on a Varian Cary 50 UV–Vis double-beam spectrophotometer (more info at: www.varianinc.com). All of the spectra were recorded at room temperature using a 10 mm path length Hellma Analytics quartz cuvettes. All of the cyclic voltammograms were recorded with a scan rate of 0.1 V/s. A typical three-electrode cell was employed, which was composed of a glassy carbon (GC) working electrode (3 mm diameter), a platinum wire as counter electrode, and a saturated aqueous calomel electrode (SCE) as reference electrode. The glass electrochemical cell was kept closed with a stopper annexed to the potentiostat. Oxygen was removed by purging the solvent with high-purity argon (Ar), introduced from a line into the cell by means of a plastic tube. Light source at 456 nm: The Kessil lamp PR160L-456 (50W) was purchased from Kessil. The photochemical reactions were carried out in borosilicate glass Schlenk tubes.

### General Procedure for the Synthesis of **3**

A 10 mL Schlenk tube was charged with radical precursors **2** (0.15 mmol, 1.0 equiv), 2-allylphenols **1** (0.225 mmol, 1.5 equiv), *N*,*N*,*N*′,*N*′-tetramethylguanidine (TMG, 0.225 mmol, 1.5 equiv), and 1,2-dichlorobenzene (600 μL [2] = 0.25 M). The reaction mixture was thoroughly degassed via three cycles of freeze–pump–thaw, and the vessel was refilled with argon and placed at 4–5 cm from a Kessil lamp (λ = 456 nm). The temperature was kept at around 30 °C by using a fan. Stirring was maintained for the indicated time (generally 30 min to 24 h) after which the irradiation was stopped. The reaction mixture was then quenched with an aqueous solution of HCl (5 mL, 1 M) and extracted with ethyl acetate (3 × 10 mL). The volatiles were removed *in vacuo*, and the residue was purified by column chromatography (cyclohexane/EtOAc) to give the desired products **3**.

#### 1-(2-(2-(Phenylsulfonyl)ethyl)-2,3-dihydrobenzofuran-5-yl)ethan-1-one **3a**

Following the general procedure applying phenol **1a** and sulfone **2a**, full conversion of **1a** was observed after 35 min. Purification by FC on silica gel (10–40% EtOAc/cyclohexane) afforded **3a** as an off-white solid (32.2 mg, 0.075 mmol, 65% yield). ^**1**^**H NMR** (CDCl_3_, 400 MHz): δ [ppm] 7.95–7.90 (m, 2H), 7.80–7.75 (m, 2H), 7.70–7.64 (m, 1H), 7.61–7.55 (m, 2H), 6.73 (d, *J* = 8.2, 1H), 4.99–4.89 (m, 1H), 3.42–3.31 (m, 2H), 3.25 (ddd, *J* = 14.1, 10.1, 5.7, 1H), 2.87 (dd, *J* = 15.6, 6.9, 1H), 2.52 (s, 3H), 2.27–2.08 (m, 2H). ^**13**^**C{**^**1**^**H} NMR** (101 MHz, CDCl_3_) δ 196.7, 163.2, 139.1, 134.1, 131.1, 130.7, 129.6, 128.1, 126.8, 125.8, 109.2, 82.1, 52.7, 34.7, 29.2, 26.5. **HRMS** (ESI-TOF) *m*/*z*: [M + Na]^+^ Calcd for C_18_H_18_O_4_SNa 353.0818; found: 353.0818.

#### 1-(2-(2-((4-Bromophenyl)sulfonyl)ethyl)-2,3-dihydrobenzofuran-5-yl)ethan-1-one **3b**

Following the general procedure applying phenol **1a** and sulfone **2b**, full conversion of **1a** was observed after 120 min. Purification by FC on silica gel (10–40% EtOAc/cyclohexane) afforded **3b** as an off-white solid (27.6 mg, 0.067 mmol, 45% yield). ^**1**^**H NMR** (CDCl_3_, 400 MHz): δ [ppm] 7.81–7.76 (m, 4H), 7.75–7.70 (m, 2H), 6.73 (d, *J* = 8.2, 1H), 5.00–4.90 (m, 1H), 3.44–3.30 (m, 2H), 3.25 (ddd, *J* = 14.0, 10.2, 5.6, 1H), 2.88 (dd, *J* = 15.8, 7.0, 1H), 2.53 (s, 3H), 2.28–2.07 (m, 2H). ^**13**^**C{**^**1**^**H} NMR** (101 MHz, CDCl_3_) δ 196.7, 163.1, 138.1, 132.9, 131.2, 130.7, 129.7, 129.5, 126.8, 125.8, 109.3, 82.0, 52.8, 34.8, 29.2, 26.6. **HRMS** (ESI-TOF) *m*/*z*: [M + Na]^+^ Calcd for C_18_H_18_^79^BrO_4_SNa 430.9924; found: 430.9927; calcd. for C_18_H_18_^81^BrO_4_SNa 432.9903; found: 432.9908.

#### 1-(2-(2-((4-Chlorophenyl)sulfonyl)ethyl)-2,3-dihydrobenzofuran-5-yl)ethan-1-one **3c**

Following the general procedure applying phenol **1a** and sulfone **2c**, full conversion of **1a** was observed after 120 min. Purification by FC on silica gel (10–40% EtOAc/cyclohexane) afforded **3c** as an off-white solid (26.8 mg, 0.074 mmol, 49% yield). ^**1**^**H NMR** (CDCl_3_, 400 MHz): δ [ppm] 7.91–7.84 (m, 2H), 7.83–7.75 (m, 2H), 7.60–7.52 (m, 2H), 6.73 (d, *J* = 8.2, 1H), 5.01–4.89 (m, 1H), 3.43–3.30 (m, 2H), 3.25 (ddd, *J* = 14.1, 10.2, 5.6, 1H), 2.88 (dd, *J* = 15.8, 7.0, 1H), 2.53 (s, 3H), 2.28–2.08 (m, 2H). ^**13**^**C{**^**1**^**H} NMR** (101 MHz, CDCl_3_) δ 196.7, 163.1, 140.9, 137.5, 131.2, 130.7, 129.9, 129.7, 126.8, 125.8, 109.3, 82.0, 52.8, 34.8, 29.2, 26.6. **HRMS** (ESI-TOF) *m*/*z*: [M + Na]^+^ Calcd for C_18_H_18_^35^ClO_4_SNa 387.0429; found: 387.0435; calcd. for C_18_H_18_^37^ClO_4_SNa 389.0399; found: 389.0403.

#### 1-(2-(2-((4-Fluorophenyl)sulfonyl)ethyl)-2,3-dihydrobenzofuran-5-yl)ethan-1-one **3d**

Following the general procedure applying phenol **1a** and sulfone **2d**, full conversion of **1a** was observed after 120 min. Purification by FC on silica gel (10–40% EtOAc/cyclohexane) afforded **3d** as an off-white solid (23.0 mg, 0.066 mmol, 44% yield). ^**1**^**H NMR** (CDCl_3_, 400 MHz): δ [ppm] 7.98–7.92 (m, 2H), 7.81–7.75 (m, 2H), 7.29–7.23 (m, 2H), 6.73 (d, *J* = 8.9, 1H), 4.99–4.91 (m, 1H), 3.43–3.31 (m, 2H), 3.25 (ddd, *J* = 14.0, 10.2, 5.6, 1H), 2.88 (dd, *J* = 15.8, 7.0, 1H), 2.53 (s, 3H), 2.27–2.07 (m, 2H). ^**13**^**C{**^**1**^**H} NMR** (101 MHz, CDCl_3_) δ 196.7, 167.4, 164.8, 163.2, 130.88 (d, *J* = 30.1), 135.2, 135.1, 131.2, 131.1, 126.8, 125.8, 116.94 (d, *J* = 22.7), 109.3, 82.0, 52.9, 34.8, 29.3, 26.5. **HRMS** (ESI-TOF) *m*/*z*: [M + Na]^+^ Calcd for C_18_H_18_FO_4_SNa 371.0724; found: 371.0724.

#### 1-(2-(2-((4-Methylphenyl)sulfonyl)ethyl)-2,3-dihydrobenzofuran-5-yl)ethan-1-one **3e**

Following the general procedure applying phenol **1a** and sulfone **2e**, full conversion of **1a** was observed after 120 min. Purification by FC on silica gel (10–40% EtOAc/cyclohexane) afforded **3e** as an off-white solid (29.9 mg, 0.087 mmol, 58% yield). ^**1**^**H NMR** (CDCl_3_, 500 MHz): δ [ppm] 7.83–7.74 (m, 4H), 7.36 (d, *J* = 8.1, 2H), 6.72 (d, *J* = 8.2, 1H), 4.98–4.89 (m, 1H), 3.39–3.28 (m, 2H), 3.22 (ddd, *J* = 13.9, 10.3, 5.5, 1H), 2.86 (dd, *J* = 15.8, 7.1, 1H), 2.52 (s, 3H), 2.44 (s, 3H), 2.25–2.07 (m, 2H). ^**13**^**C{**^**1**^**H} NMR** (125 MHz, CDCl_3_) δ 196.7, 163.2, 145.1, 136.1, 131.1, 130.6, 130.2, 128.2, 126.9, 125.8, 109.2, 82.2, 52.8, 34.7, 29.3, 26.5, 21.8. **HRMS** (ESI-TOF) *m*/*z*: [M + Na]^+^ Calcd for C_19_H_20_O_4_SNa 367.0975; found: 367.0976.

#### 1-(2-(2-(Naphthalen-1-ylsulfonyl)ethyl)-2,3-dihydrobenzofuran-5-yl)ethan-1-one **3f**

Following the general procedure applying phenol **1a** and sulfone **2f**, full conversion of **1a** was observed after 60 min. Purification by FC on silica gel (10–40% EtOAc/cyclohexane) afforded **3f** as an off-white solid (17.1 mg, 0.045 mmol, 30% yield). ^**1**^**H NMR** (CDCl_3_, 500 MHz): δ [ppm] 8.74 (d, *J* = 8.7, 1H), 8.32 (dd, *J* = 7.3, 1.3, 1H), 8.15 (d, *J* = 8.2, 1H), 7.99 (d, *J* = 7.4, 1H), 7.79–7.69 (m, 3H), 7.67–7.58 (m, 2H), 6.67 (d, *J* = 8.3, 1H), 4.97–4.89 (m, 1H), 3.55 (ddd, *J* = 14.0, 10.0, 5.5, 1H), 3.47 (ddd, *J* = 14.1, 9.8, 5.8, 1H), 3.33 (dd, *J* = 15.7, 9.1, 1H), 2.83 (dd, *J* = 15.7, 7.0, 1H), 2.51 (s, 3H), 2.26–2.10 (m, 2H). ^**13**^**C{**^**1**^**H} NMR** (125 MHz, CDCl_3_) δ 196.7, 163.2, 135.6, 134.4, 134.0, 131.1, 130.9, 130.7, 129.5, 129.03, 128.99, 127.3, 126.9, 125.8, 124.6, 124.1, 109.2, 82.1, 52.3, 34.7, 29.3, 26.5. **HRMS** (ESI-TOF) *m*/*z*: [M + Na]^+^ Calcd for C_22_H_20_O_4_SNa 403.0975; found: 403.0976.

#### 1-(2-(2-(Methylsulfonyl)ethyl)-2,3-dihydrobenzofuran-5-yl)ethan-1-one **3g**

Following the general procedure applying phenol **1a** and sulfone **2g**, full conversion of **1a** was observed after 120 min. Purification by FC on silica gel (10–40% EtOAc/cyclohexane) afforded **3g** as an off-white solid (24.9 mg, 0.093 mmol, 62% yield). ^**1**^**H NMR** (CDCl_3_, 500 MHz): δ [ppm] 7.84–7.78 (m, 2H), 6.78 (d, *J* = 8.3, 1H), 5.06–4.98 (m, 1H), 3.43 (dd, *J* = 15.8, 9.2, 1H), 3.30 (ddd, *J* = 13.8, 10.2, 5.2, 1H), 3.20 (ddd, *J* = 13.8, 10.1, 5.7, 1H), 2.97–2.90 (m, 4H), 2.53 (s, 3H), 2.33 (dddd, *J* = 14.0, 10.0, 5.7, 3.8, 1H), 2.24 (dddd, *J* = 14.0, 10.0, 8.9, 5.2, 1H). ^**13**^**C{**^**1**^**H} NMR** (125 MHz, CDCl_3_) δ 196.7, 163.2, 131.2, 130.7, 126.8, 125.9, 109.3, 82.0, 51.1, 41.1, 34.8, 28.8, 26.5. **HRMS** (ESI-TOF) *m*/*z*: [M + Na]^+^ Calcd for C_13_H_16_O_4_SNa 291.0662; found: 291.0661.

#### 1-(2-(2-(Cyclohexylsulfonyl)ethyl)-2,3-dihydrobenzofuran-5-yl)ethan-1-one **3h**

Following the general procedure applying phenol **1a** and sulfone **2h**, full conversion of **1a** was observed after 60 min. Purification by FC on silica gel (10–40% EtOAc/cyclohexane) afforded **3h** as an off-white solid (31.8 mg, 0.095 mmol, 63% yield). ^**1**^**H NMR** (CDCl_3_, 500 MHz): δ [ppm] 7.85–7.75 (m, 2H), 6.76 (d, *J* = 8.3, 1H), 5.04–4.97 (m, 1H), 3.41 (dd, *J* = 15.8, 9.2, 1H), 3.18 (ddd, *J* = 13.4, 10.3, 5.2, 1H), 3.07 (ddd, *J* = 13.4, 10.1, 5.6, 1H), 2.93 (dd, *J* = 15.8, 7.0, 1H), 2.89–2.82 (m, 1H), 2.53 (s, 3H), 2.31 (dddd, *J* = 14.1, 9.9, 5.6, 3.8, 1H), 2.27–2.14 (m, 3H), 1.98–1.89 (m, 2H), 1.79–1.68 (m, 1H), 1.61–1.50 (m, 2H), 1.36–1.16 (m, 3H). ^**13**^**C{**^**1**^**H} NMR** (125 MHz, CDCl_3_) δ 196.7, 163.2, 131.1, 130.6, 126.9, 125.8, 109.2, 82.5, 61.6, 45.6, 34.8, 27.8, 26.5, 25.3, 25.2, 25.14, 25.12. **HRMS** (ESI-TOF) *m*/*z*: [M + Na]^+^ Calcd for C_18_H_24_O_4_SNa 359.1288; found: 359.1283.

#### 2-(2-(Phenylsulfonyl)ethyl)-2,3-dihydrobenzofuran **3i**

This reaction was carried out on a 0.5 mmol scale of sulfone **2a**. Following the general procedure applying phenol **1b** and sulfone **2a**, full conversion of **1b** was observed after 120 min. Purification by FC on silica gel (10–40% EtOAc/cyclohexane) afforded **3i** as a colorless oil (70.5 mg, 0.245 mmol, 49% yield). ^**1**^**H NMR** (CDCl_3_, 500 MHz): δ [ppm] = 7.70–7.64 (m, 1H), 7.62–7.56 (m, 2H), 7.15–7.12 (m, 1H), 7.11–7.06 (m, 1H), 6.85–6.81 (m, 1H), 6.71 (d, *J* = 7.9, 1H), 4.90–4.79 (m, 1H), 3.41–3.30 (m, 3H), 3.26 (ddd, *J* = 14.0, 10.5, 5.3, 1H), 2.85 (dd, *J* = 15.6, 7.2, 1H), 2.24–2.08 (m, 3H). ^**13**^**C{**^**1**^**H} NMR** (125 MHz, CDCl_3_) δ 159.0, 139.2, 134.0, 129.5, 128.3, 128.1, 126.0, 125.1, 120.8, 109.6, 80.6, 52.9, 35.4, 29.2. **HRMS** (ESI-TOF) *m*/*z*: [M + Na]^+^ Calcd for C_16_H_16_O_3_SNa 311.0712; found: 311.0713.

#### 2-(2-(Methylsulfonyl)ethyl)-2,3-dihydrobenzofuran **3j**

Following the general procedure applying phenol **1b** and sulfone **2g**, full conversion of **1b** was observed after 120 min. Purification by FC on silica gel (10–40% EtOAc/cyclohexane) afforded **3j** as an off-white solid (20.0 mg, 0.089 mmol, 59% yield). ^**1**^**H NMR** (CDCl_3_, 500 MHz): δ [ppm] 7.17 (d, *J* = 7.4, 1H), 7.15–7.09 (m, 1H), 6.89–6.82 (m, 1H), 6.76 (d, *J* = 7.7, 1H), 4.95–4.86 (m, 1H), 3.39 (dd, *J* = 15.7, 9.1, 1H), 3.31 (ddd, *J* = 13.8, 10.4, 5.3, 1H), 3.19 (ddd, *J* = 14.0, 10.2, 5.7, 1H), 2.96–2.86 (m, 4H), 2.35–2.16 (m, 2H). ^**13**^**C{**^**1**^**H} NMR** (125 MHz, CDCl_3_) δ 158.9, 128.4, 126.0, 125.2, 120.9, 109.6, 80.5, 51.3, 41.0, 35.4, 28.8. **HRMS** (ESI-TOF) *m*/*z*: [M + Na]^+^ Calcd for C_11_H_14_O_3_SNa 249.0556; found: 249.0560.

#### 5-Bromo-2-(2-(methylsulfonyl)ethyl)-2,3-dihydrobenzofuran **3k**

Following the general procedure applying phenol **1c** and sulfone **2g**, full conversion of **1c** was observed after 120 min. Purification by FC on silica gel (10–40% EtOAc/cyclohexane) afforded **3k** as an off-white solid (22.4 mg, 0.074 mmol, 49% yield). ^**1**^**H NMR** (CDCl_3_, 500 MHz): δ [ppm] 7.30–7.24 (m, 1H), 7.24–7.18 (m, 1H), 6.63 (d, *J* = 8.4, 1H), 4.97–4.87 (m, 1H), 3.38 (dd, *J* = 15.9, 9.4, 1H), 3.28 (ddd, *J* = 13.8, 10.3, 5.2, 1H), 3.18 (ddd, *J* = 13.9, 10.2, 5.7, 1H), 2.94 (s, 3H), 2.89 (dd, *J* = 15.9, 7.0, 1H), 2.32–2.16 (m, 2H). ^**13**^**C{**^**1**^**H} NMR** (125 MHz, CDCl_3_) δ 158.2, 131.2, 128.5, 128.2, 112.7, 111.2, 81.3, 51.2, 41.1, 35.3, 28.7. **HRMS** (ESI-TOF) *m*/*z*: [M + Na]^+^ Calcd for C_11_H_13_^79^BrO_3_SNa 326.9661; found: 326.9660; calcd. for C_11_H_13_^81^BrO_3_S + Na 328.9641; found: 328.9650.

#### 5-Chloro-2-(2-(methylsulfonyl)ethyl)-2,3-dihydrobenzofuran **3l**

Following the general procedure applying phenol **1d** and sulfone **2g**, full conversion of **1d** was observed after 120 min. Purification by FC on silica gel (10–40% EtOAc/cyclohexane) afforded **3l** as an off-white solid (22.4 mg, 0.074 mmol, 49% yield). ^**1**^**H NMR** (CDCl_3_, 500 MHz): δ [ppm] 7.14–7.11 (m, 1H), 7.09–7.05 (m, 1H), 6.66 (d, *J* = 8.5, 1H), 4.98–4.89 (m, 1H), 3.37 (dd, *J* = 15.9, 9.2, 1H), 3.29 (ddd, *J* = 13.8, 10.3, 5.2, 1H), 3.18 (ddd, *J* = 13.9, 10.2, 5.7, 1H), 2.94 (s, 3H), 2.89 (dd, *J* = 15.9, 7.0, 1H), 2.34–2.23 (m, 1H), 2.24–2.16 (m, 1H). ^**13**^**C{**^**1**^**H} NMR** (125 MHz, CDCl_3_) δ 157.6, 128.3, 128.0, 125.6, 125.3, 110.5, 81.3, 51.2, 41.1, 35.4, 28.7. **HRMS** (ESI-TOF) *m*/*z*: [M + Na]^+^ Calcd for C_11_H_13_^35^ClO_3_SNa 283.0167; found: 283.0166; calcd. for C_11_H_13_^37^ClO_4_S + Na 285.0137; found: 285.0135.

#### 5-Methoxy-2-(2-(methylsulfonyl)ethyl)-2,3-dihydrobenzofuran **3m**

Following the general procedure applying phenol **1e** and sulfone **2g**, full conversion of **1e** was observed after 360 min. Purification by FC on silica gel (10–40% EtOAc/cyclohexane) afforded **3m** as an off-white solid (11.9 mg, 0.047 mmol, 31% yield). ^**1**^**H NMR** (CDCl_3_, 500 MHz): δ [ppm] 6.77–6.74 (m, 1H), 6.66–6.65 (m, 2H), 4.92–4.84 (m, 1H), 3.75 (s, 3H), 3.36 (dd, *J* = 15.7, 9.0, 1H), 3.30 (ddd, *J* = 13.8, 10.4, 5.2, 1H), 3.22–3.14 (m, 1H), 2.94 (s, 3H), 2.88 (dd, *J* = 15.8, 7.0, 1H), 2.33–2.16 (m, 2H). ^**13**^**C{**^**1**^**H} NMR** (125 MHz, CDCl_3_) δ 154.5, 153.1, 127.0, 113.3, 111.5, 109.5, 80.7, 56.2, 51.3, 41.0, 35.9, 28.8. **HRMS** (ESI-TOF) *m*/*z*: [M + Na]^+^ Calcd for C_12_H_16_O_4_SNa 279.0662; found: 279.0663.

#### 2-(2-(Methylsulfonyl)ethyl)-2,3-dihydrobenzofuran-5-carbonitrile **3n**

Following the general procedure applying phenol **1f** and sulfone **2g**, full conversion of **1f** was observed after 120 min. Purification by FC on silica gel (10–40% EtOAc/cyclohexane) afforded **3n** as an off-white solid (26.0 mg, 0.10 mmol, 69% yield). ^**1**^**H NMR** (CDCl_3_, 500 MHz): δ [ppm] 7.47–7.43 (m, 2H), 6.83–6.79 (m, 1H), 5.08–4.99 (m, 1H), 3.43 (dd, *J* = 16.0, 9.2, 1H), 3.29 (ddd, *J* = 13.9, 10.1, 5.3, 1H), 3.20 (ddd, *J* = 13.8, 9.9, 5.9, 1H), 2.97–2.89 (m, 4H), 2.33 (dddd, *J* = 13.9, 9.9, 5.9, 3.8, 1H), 2.28–2.18 (m, 1H). ^**13**^**C{**^**1**^**H} NMR** (125 MHz, CDCl_3_) δ 162.5, 133.9, 129.2, 127.8, 119.4, 110.6, 104.3, 82.1, 51.0, 41.2, 34.7, 28.6. **HRMS** (ESI-TOF) *m*/*z*: [M + Na]^+^ Calcd for C_12_H_13_NO_3_SNa 274.0509; found: 274.0507.

#### Methyl 2-(2-(methylsulfonyl)ethyl)-2,3-dihydrobenzofuran-7-carboxylate **3o**

Following the general procedure applying phenol **1g** and sulfone **2g**, full conversion of **1g** was observed after 120 min. Purification by FC on silica gel (10–50% EtOAc/cyclohexane and then re-purified 10–20% EtOAc/CH_2_Cl_2_) afforded **3o** as an off-white solid (17.6 mg, 0.062 mmol, 41% yield). ^**1**^**H NMR** (CDCl_3_, 500 MHz): δ [ppm] 7.73 (d, *J* = 7.9, 1H), 7.33 (dd, *J* = 7.3, 1.3, 1H), 6.92–6.87 (m, 1H), 5.11–5.03 (m, 1H), 3.89 (s, 3H), 3.42 (dd, *J* = 15.8, 9.2, 1H), 3.35 (ddd, *J* = 13.9, 10.4, 5.2, 1H), 3.24 (ddd, *J* = 13.9, 10.2, 5.6, 1H), 2.96 (s, 3H), 2.92 (ddt, *J* = 15.8, 6.5, 1.1, 1H), 2.38–2.20 (m, 2H). ^**13**^**C{**^**1**^**H} NMR** (125 MHz, CDCl_3_) δ 165.6, 159.4, 130.2, 129.7, 128.4, 120.7, 113.4, 81.8, 52.0, 51.2, 41.1, 34.7, 28.8. **HRMS** (ESI-TOF) *m*/*z*: [M + Na]^+^ Calcd for C_13_H_16_O_5_SNa 307.0611; found: 3070612.

#### 2-(2-(Methylsulfonyl)ethyl)-2,3-dihydrobenzofuran-7-carbaldehyde **3p**

Following the general procedure applying phenol **1h** and sulfone **2g**, full conversion of **1h** was observed after 30 min. Purification by FC on silica gel (10–40% EtOAc/cyclohexane) afforded **3p** as an off-white solid (11.1 mg, 0.044 mmol, 29% yield). ^**1**^**H NMR** (CDCl_3_, 500 MHz): δ [ppm] 10.18 (s, 1H), 7.60 (d, *J* = 7.8, 1H), 7.43–7.35 (m, 1H), 6.96 (t, *J* = 7.5, 1H), 5.16–5.07 (m, 1H), 3.43 (dd, *J* = 15.9, 9.2, 1H), 3.34 (ddd, *J* = 13.8, 10.2, 5.3, 1H), 3.25 (ddd, *J* = 13.8, 10.0, 5.7, 1H), 3.00–2.91 (m, 4H), 2.37 (dddd, *J* = 14.1, 9.9, 5.7, 3.9, 1H), 2.33–2.24 (m, 1H). ^**13**^**C{**^**1**^**H} NMR** (125 MHz, CDCl_3_) δ 188.7, 160.9, 131.2, 128.5, 127.8, 121.3, 119.9, 82.8, 51.1, 41.2, 34.4, 28.6. **HRMS** (ESI-TOF) *m*/*z*: [M + Na]^+^ Calcd for C_12_H_14_O_4_SNa 277.0505; found: 277.0504.

#### 1-(2-(7,7,7,7,7,7,7,7,7,7,7,7,7-Tridecafluoro-7l16-hepta-2,4,6-triyn-1-yl)-2,3-dihydrobenzofuran-5-yl)ethan-1-one **3q**

Following the general procedure applying phenol **1a** and perfluorohexyl iodide, the reaction was stirred for 16 h. Purification by FC on silica gel (10% EtOAc/cyclohexane) afforded **3q** as a white solid (30.0 mg, 0.060 mmol, 40% yield). ^**1**^**H NMR** (CDCl_3_, 500 MHz): δ [ppm] 7.86–7.80 (m, 2H), 6.83 (d, *J* = 8.3, 1H), 5.29–5.21 (m, 1H), 3.52 (dd, *J* = 15.9, 9.1, 1H), 3.05 (dd, *J* = 15.9, 7.5, 1H), 2.80–2.66 (m, 1H), 2.56–2.41 (m, 4H). ^**19**^**F NMR** (CDCl_3_, 376 MHz): δ [ppm] 80.73 to −80.82 (m, 3F), −112.37 to −112.79 (m, 2F), −121.61 to −121.97 (m, 2F), −122.72 to −122.96 (m, 2F), −123.37 to −123.66 (m, 2F), −125.99 to −126.22 (m, 2F). ^**13**^**C{**^**1**^**H} NMR** (125 MHz, CDCl_3_) δ 196.7, 163.0, 131.5, 130.9, 126.5, 125.7, 109.5, 37.2 (t, *J* = 21.3), 35.7, 26.6. **HRMS** (ESI-TOF) *m*/*z*: [M + Na]^+^ Calcd for C_17_H_11_F_13_O_2_Na 517.0444; found: 517.0444.

#### 2-(7,7,7,7,7,7,7,7,7,7,7,7,7-Tridecafluoro-7l16-hepta-2,4,6-triyn-1-yl)-2,3-dihydrobenzofuran-5-carbonitrile **3r**

Following the general procedure applying phenol **1a** and perfluorohexyl iodide, the reaction was stirred for 240 min. Purification by FC on silica gel (10% EtOAc/cyclohexane) afforded **3r** as a white solid (26.5 mg, 0.056 mmol, 37% yield). ^**1**^**H NMR** (CDCl_3_, 500 MHz): δ [ppm] 7.52–7.45 (m, 2H), 6.86 (d, *J* = 7.9, 1H), 5.30–5.22 (m, 1H), 3.52 (dd, *J* = 16.1, 9.1, 1H), 3.06 (dd, *J* = 16.1, 7.7, 1H), 2.83–2.65 (m, 1H), 2.59–2.38 (m, 1H). ^**19**^**F NMR** (CDCl_3_, 376 MHz): δ [ppm] −80.64 to −80.81 (m, 3F), −111.21 to −111.50 (m, 1F), −112.25 to −112.67 (m, 1F), −121.52 to −121.89 (m, 2F), −122.53 to −123.01 (m, 2F), −123.24 to −123.57 (m, 2F), −125.98 to −126.14 (m, 2F). ^**13**^**C{**^**1**^**H} NMR** (125 MHz, CDCl_3_) δ 162.4, 134.1, 129.1, 127.4, 119.4, 110.8, 104.6, 37.1 (t, *J* = 21.0), 35.6. **HRMS** (ESI-TOF) *m*/*z*: [M + Na]^+^ Calcd for C_16_H_8_F_13_NONa 500.0291; found: 500.0290.

#### 1-(2-(2,2-Dibromovinyl)-2,3-dihydrobenzofuran-5-yl)ethan-1-one **3s**

Following the general procedure applying phenol **1a** and tetrabromomethane, the reaction was stirred for 240 min. Purification by FC on silica gel (10% EtOAc/cyclohexane) afforded **3s** as a white solid (18.2 mg, 0.053 mmol, 35% yield). ^**1**^**H NMR** (CDCl_3_, 500 MHz): 7.85–7.79 (m, 2H), 6.82 (d, *J* = 8.3, 1H), 6.71 (d, *J* = 8.0, 1H), 5.48 (ddd, *J* = 9.4, 7.9, 7.0, 1H), 3.55 (dd, *J* = 15.8, 9.4, 1H), 3.05 (dd, *J* = 15.8, 7.0, 1H), 2.54 (s, 3H). ^**13**^**C{1H} NMR** (125 MHz, CDCl_3_) δ 196.7, 163.1, 137.4, 131.4, 130.8, 126.7, 125.7, 109.4, 93.7, 83.4, 34.8, 26.6. **HRMS** (ESI-TOF) *m*/*z*: [M + Na]^+^ Calcd for C_12_H_10_^79^Br^79^BrO_2_Na 366.8940; found: 366.8943; calcd. for C_12_H_10_^79^Br^81^BrO_2_Na 368.8920; found: 368.8924; calcd. for C_12_H_10_^81^Br^81^BrO_2_Na 370.8899; found: 370.8906

#### 1-(2-(2,2-Dichlorovinyl)-2,3-dihydrobenzofuran-5-yl)ethan-1-one **3t**

Following the general procedure applying phenol **1a** and bromotrichloromethane, the reaction was stirred for 240 min. Purification by FC on silica gel (10% EtOAc/cyclohexane) afforded **3t** as a white solid (15.4 mg, 0.06 mmol, 40% yield). ^**1**^**H NMR** (CDCl_3_, 500 MHz): δ [ppm] 7.87–7.77 (m, 2H), 6.82 (d, *J* = 8.3, 1H), 6.15 (d, *J* = 8.3, 1H), 5.60 (ddd, *J* = 9.3, 8.3, 7.2, 1H), 3.54 (dd, *J* = 15.9, 9.4, 1H), 3.04 (dd, *J* = 16.0, 7.2, 1H), 2.54 (s, 3H). ^**13**^**C{**^**1**^**H} NMR** (125 MHz, CDCl_3_) δ 196.7, 163.1, 131.4, 130.8, 128.9, 126.8, 125.7, 125.3, 109.4, 80.8, 35.1, 26.6. **HRMS** (ESI-TOF) *m*/*z*: [M + Na]^+^ Calcd for C_12_H_10_^35^Cl^35^ClO_2_Na 278.9951; found: 278.9951; calcd. for C_12_H_10_^35^Cl^37^ClO_2_Na 280.9921; found: 280.9919; calcd. for C_12_H_10_^37^Cl^37^ClO_2_Na 282.9892; found: 282.9888.

#### 1-(3-((1,1-Dioxidothiochroman-4-yl)methyl)-4-hydroxyphenyl)ethan-1-one **3aa**

Purification by FC on silica gel (10–40% EtOAc/cyclohexane) afforded the side product as a white solid in less than 10% yield ^**1**^**H NMR** (DMSO-*d*_6_, 500 MHz): δ [ppm] 10.56 (br s, 1H), 7.85 (d, *J* = 2.2, 1H), 7.80 (dd, *J* = 7.9, 1.3, 1H), 7.75 (dd, *J* = 8.4, 2.3, 1H), 7.64–7.59 (m, 1H), 7.56 (d, *J* = 7.3, 1H), 7.52–7.48 (m, 1H), 6.94 (d, *J* = 8.5, 1H), 3.75 (ddd, *J* = 14.6, 12.0, 2.8, 1H), 3.48–3.35 (m, 2H), 3.04 (dd, *J* = 13.4, 4.5, 1H), 2.89 (dd, *J* = 13.4, 10.9, 1H), 2.48 (s, 3H), 2.34–2.24 (m, 1H), 2.04–1.96 (m, 1H). ^**13**^**C{**^**1**^**H} NMR** (DMSO-*d*_6_, 125 MHz) δ 196.2, 160.2, 140.5, 138.2, 132.4, 131.9, 129.9, 129.0, 128.6, 127.7, 125.5, 122.8, 114.9, 46.1, 36.2, 35.7, 26.3, 23.5. **HRMS** (ESI-TOF) *m*/*z*: [M + H]^+^ Calcd for C_18_H_18_O_4_SH 331.1004; found: 331.0992.

#### Procedure for the Synthesis of **4a**

To a round-bottom flask containing freshly activated Mg (30 equiv, 11.25 mmol, 273 mg) under Ar was added a solution of **3i** (1 equiv, 0.375 mmol, 108 mg) in dry MeOH (6.0 mL) followed by anhydrous NiCl_2_ (0.5 equiv, 0.188 mmol, 24 mg). The mixture was stirred vigorously at 60 °C for 6 h, in an oil bath. The reaction was quenched by adding an aqueous solution of HCl (1 M). The crude mixture was then transferred to a separatory funnel and extracted with CH_2_Cl_2_ (3 times). The organic phases were combined and dried over Mg_2_SO_4_ before concentration *in vacuo*. The residue was purified by flash chromatography (50% CH_2_Cl_2_ in petroleum ether) to give **4a**.

#### 2-Ethyl-2,3-dihydrobenzofuran **4a**

Following the procedure for the desulfonylation applying product **3i** (0.375 mmol), the reaction was stirred for 5 h. After workup, **4a** was obtained as a colorless oil (22.7 mg, 0.154 mmol, 41% yield). ^**1**^**H NMR** (CDCl_3_, 400 MHz): δ [ppm] 7.16 (d, *J* = 7.3, 1H), 7.13–7.07 (m, 1H), 6.85–6.79 (m, 1H), 6.76 (d, *J* = 8.0, 1H), 4.77–4.66 (m, 1H), 3.27 (dd, *J* = 15.5, 8.9, 1H), 2.87 (dd, *J* = 15.6, 7.8, 1H), 1.92–1.80 (m, 1H), 1.79–1.66 (m, 1H), 1.04 (t, *J* = 7.4, 3H). ^**13**^**C{**^**1**^**H} NMR** (101 MHz, CDCl_3_) δ 159.8, 128.0, 127.1, 125.0, 120.2, 109.3, 84.7, 35.1, 29.1, 9.8. **HRMS** (ESI-TOF) *m*/*z*: [M + Na]^+^ Calcd for C_10_H_12_ONa 149.0961; found: 149.0961.

#### Procedure for the Synthesis of **4b** and **4c**

In a small Schlenk tube equipped with a magnetic stirring bar, compound **3k** (38 mg, 1.0 equiv, 0.12 mmol), toluene (450 μL), 1,4-dioxane (50 μL), the corresponding arylboronic acid (2.5 equiv, 0.30 mmol), and K_2_CO_3_ (2 M aqueous solution, 150 μL, 2.5 equiv, 0.30 mmol) were added in this order. The whole reaction mixture was then degassed using the freezing-pump method (3 times), and Pd(PPh_3_)_4_ (7.0 mg, 5 mol %) was added. The resulting biphasic mixture was vigorously stirred under argon atmosphere at 100 °C, in an oil bath, for 7 h, and then cooled to room temperature. The crude mixture was passed through a short plug of SiO_2_ eluted with CH_2_Cl_2_ (10 mL) and EtOAc (3 × 5 mL), evaporated *in vacuo*, and purified by column chromatography on silica gel (EtOAc/cyclohexane mixtures) to afford the desired products **4b** and **4c**.

#### 2-(2-(Methylsulfonyl)ethyl)-5-phenyl-2,3-dihydrobenzofuran **4b**

Substrate **3k** and phenylboronic acid were reacted for 7 h following the procedure for the Suzuki–Miyaura coupling. Purification by FC on silica gel (10–50% EtOAc/cyclohexane) followed by another FC on silica gel (1–5% Et_2_O/CH_2_Cl_2_) afforded **4b** as a white solid (19.6 mg, 0.065 mmol, 54% yield). ^**1**^**H NMR** (CDCl_3_, 500 MHz): δ [ppm] 7.53–7.49 (m, 2H), 7.45–7.39 (m, 3H), 7.38–7.34 (m, 1H), 7.33–7.27 (m, 1H), 6.82 (d, *J* = 8.3, 1H), 5.03–4.92 (m, 1H), 3.46 (dd, *J* = 15.7, 9.1, 1H), 3.38–3.29 (m, 1H), 3.27–3.18 (m, 1H), 3.01–2.93 (m, 4H), 2.40–2.19 (m, 2H). ^**13**^**C{**^**1**^**H} NMR** (125 MHz, CDCl_3_) δ 158.6, 141.2, 134.7, 128.9, 127.6, 126.9, 126.8, 126.7, 124.1, 109.8, 81.1, 51.3, 41.1, 35.5, 28.9. **HRMS** (ESI-TOF) *m*/*z*: [M + Na]^+^ Calcd for C_17_H_18_O_3_SNa 325.0869; found: 325.0867.

#### 2-(2-(Methylsulfonyl)ethyl)-5-phenyl-2,3-dihydrobenzofuran **4c**

Substrate **3k** and 4-methoxycarbonylphenylboronic acid were reacted for 7 h following the procedure for the Suzuki–Miyaura coupling. Purification by FC on silica gel (10–50% EtOAc/cyclohexane) followed by another FC on silica gel (1–5% Et_2_O/CH_2_Cl_2_) afforded **4c** as a white solid (20.7 mg, 0.06 mmol, 48% yield). ^**1**^**H NMR** (CDCl_3_, 500 MHz): δ [ppm] 8.10–8.05 (m, 2H), 7.61–7.56 (m, 2H), 7.46–7.43 (m, 1H), 7.42–7.38 (m, 1H), 6.84 (d, *J* = 8.3, 1H), 5.04–4.95 (m, 1H), 3.93 (s, 3H), 3.47 (dd, J = 15.8, 9.1, 1H), 3.38–3.29 (m, 1H), 3.27–3.18 (m, 1H), 3.03–2.93 (m, 4H), 2.39–2.21 (m, 2H). ^**13**^**C{**^**1**^**H} NMR** (125 MHz, CDCl_3_) δ 167.2, 159.4, 145.6, 133.3, 130.3, 128.4, 127.9, 127.1, 126.7, 124.2, 110.0, 81.3, 52.2, 51.3, 41.1, 35.4, 28.8. **HRMS** (ESI-TOF) *m*/*z*: [M + Na]^+^ Calcd for C_19_H_20_O_5_SNa 383.0924; found: 383.0921.

#### Procedure for the Synthesis of **4d**

Bromoalkene (**3s**, 64 mg, 0.185 mmol) was dissolved in 0.925 mL of DMF (0.2 M). TBAF·3H_2_O (0.291 g, 0.925 mmol) was added to the solution, and the reaction mixture was heated at 60 °C, in an oil bath, for 2 h (TLC). The reaction mixture was cooled to room temperature and diluted with diethyl ether (10 mL). The organic phase was washed with water and brine, dried over anhydrous MgSO_4_, filtered, and concentrated under reduced pressure. The residue was purified by flash chromatography (5–10% ethyl acetate in cyclohexane) to give **4d** (15.8 mg, 46%) as a colorless oil. ^**1**^**H NMR** (CDCl_3_, 500 MHz): δ [ppm] 7.86–7.80 (m, 2H), 6.85 (d, *J* = 8.1, 1H), 5.44 (ddd, *J* = 9.6, 7.0, 2.2, 1H), 3.56 (dd, *J* = 15.5, 9.6, 1H), 3.35 (dd, *J* = 15.6, 7.0, 1H), 2.64 (d, *J* = 2.2, 1H), 2.54 (s, 3H). ^**13**^**C{**^**1**^**H} NMR** (125 MHz, CDCl_3_) δ 196.7, 162.7, 131.5, 130.8, 126.4, 125.6, 109.6, 81.6, 75.3, 72.5, 37.0, 26.6. **HRMS** (ESI-TOF) *m*/*z*: [M + Na]^+^ Calcd for C_12_H_10_O_2_Na 209.0573; found: 209.0574.

## Data Availability

The data underlying this study are available in the published article and its Supporting Information.

## Supporting Information Available

General information, general procedures, characterization data, optimization and mechanistic studies, and product manipulation protocols (PDF)

## References

[ref1] Tyman J. H. P. Synthetic and Natural Phenols; Elsevier, 1996.

[ref2] Soto-Hernández M.; Tenango M. P.; García-Mateos R. Phenolic Compounds: Biological Activity; BoD – Books on Demand, 2017.

[ref3] Rappoport Z. The Chemistry of Phenols 2; John Wiley & Sons Ltd., 2003.

[ref4] Quideau S.; Deffieux D.; Douat-Casassus C.; Pouységu L. Plant polyphenols: Chemical properties, biological activities, and synthesis. Angew. Chem., Int. Ed. 2011, 50, 586–621. 10.1002/anie.201000044. 21226137

[ref5] Yan J.; Meng Q.; Shen X.; Chen B.; Sun Y.; Xiang J.; Liu H.; Han B. Selective valorization of lignin to phenol by direct transformation of Csp2-Csp3 and C-O bonds. Sci. Adv. 2020, 6, eabd1951 10.1126/sciadv.abd1951. 33158871 PMC7673717

[ref6] Huang Z.; Lumb J. P. Phenol-Directed C-H Functionalization. ACS Catal. 2019, 9, 521–555. 10.1021/acscatal.8b04098.

[ref7] Bartolomei B.; Gentile G.; Rosso C.; Filippini G.; Prato M. Turning the Light on Phenols: New Opportunities in Organic Synthesis. Chem. – Eur. J. 2021, 27, 16062–16070. 10.1002/chem.202102276. 34339553

[ref8] Balzani V.; Ceroni P.; Juris A. Photochemistry and Photophysics: Concepts, Research, Applications; John Wiley & Sons, 2014.

[ref9] aLiang K.; Li T.; Li N.; Zhang Y.; Shen L.; Ma Z.; Xia C. Redox-neutral photochemical Heck-type arylation of vinylphenols activated by visible light. Chem. Sci. 2020, 11, 2130–2135. 10.1039/C9SC06184C. 34123301 PMC8150107

[ref10] aSchmalzbauer M.; Ghosh I.; König B. Utilising excited state organic anions for photoredox catalysis: Activation of (hetero)aryl chlorides by visible light-absorbing 9-anthrolate anions. Faraday Discuss. 2019, 215, 364–378. 10.1039/C8FD00176F. 30957806

[ref11] aShen N.; Li R.; Liu C.; Shen X.; Guan W.; Shang R. Photocatalytic Cross-Couplings of Aryl Halides Enabled by o-Phosphinophenolate and o-Phosphinothiophenolate. ACS Catal. 2022, 12, 2788–2795. 10.1021/acscatal.1c05941.

[ref12] aGuo Q.; Wang M.; Liu H.; Wang R.; Xu Z. Visible-Light-Promoted Dearomative Fluoroalkylation of β-Naphthols through Intermolecular Charge Transfer. Angew. Chem., Int. Ed 2018, 57, 4747–4751. 10.1002/anie.201800767. 29476596

[ref13] Crisenza G. E. M.; Mazzarella D.; Melchiorre P. Synthetic Methods Driven by the Photoactivity of Electron Donor-Acceptor Complexes. J. Am. Chem. Soc. 2020, 142, 5461–5476. 10.1021/jacs.0c01416. 32134647 PMC7099579

[ref14] Lima C. G. S.; Lima T. D. M.; Duarte M.; Jurberg I. D.; Paixão M. W. Organic Synthesis Enabled by Light-Irradiation of EDA Complexes: Theoretical Background and Synthetic Applications. ACS Catal. 2016, 6, 1389–1407. 10.1021/acscatal.5b02386.

[ref15] Chen Z.; Pitchakuntla M.; Jia Y. Synthetic approaches to natural products containing 2,3-dihydrobenzofuran skeleton. Nat. Prod. Rep. 2019, 36, 666–690. 10.1039/C8NP00072G. 30488047

[ref16] Dapkekar A. B.; Sreenivasulu C.; Ravi Kishore D.; Satyanarayana G. Recent Advances Towards the Synthesis of Dihydrobenzofurans and Dihydroisobenzofurans. Asian J. Org. Chem. 2022, 11, e202200012 10.1002/ajoc.202200012.

[ref17] Buzzetti L.; Crisenza G. E. M.; Melchiorre P. Mechanistic Studies in Photocatalysis. Angew. Chem., Int. Ed. 2019, 58, 3730–3747. 10.1002/anie.201809984. 30339746

[ref18] Filippini G.; Silvi M.; Melchiorre P. Enantioselective Formal α-Methylation and α-Benzylation of Aldehydes by Means of Photo-organocatalysis. Angew. Chem., Int. Ed. 2017, 56, 4447–4451. 10.1002/anie.201612045. PMC5396335 28323367

[ref19] Gui J.; Zhou Q.; Pan C. M.; Yabe Y.; Burns A. C.; Collins M. R.; Ornelas M. A.; Ishihara Y.; Baran P. S. C-H methylation of heteroarenes inspired by radical SAM methyl transferase. J. Am. Chem. Soc. 2014, 136, 4853–4856. 10.1021/ja5007838. 24611732 PMC3988686

