# Supplementary material for: Photoinduced Cascade Reactions of 2-Allylphenol Derivatives toward the Production of 2,3-Dihydrobenzofurans
Source: J Org Chem. 2023 Mar 31;88(9):6008–16. doi: 10.1021/acs.joc.3c00347 (PMC10167682; doi:10.1021/acs.joc.3c00347)

Photo-Induced Cascade Reactions of 2-Allylphenol Derivatives Towards the Production of 2,3-Dihydrobenzofurans

Vasco Corti [‡], Jacopo Dosso[‡], Maurizio Prato [‡, #,⊥], and Giacomo Filippini[‡*]

[‡] Department of Chemical and Pharmaceutical Sciences, CENMAT, Center of Excellence for Nanostructured Materials, INSTM UdR, Trieste, University of Trieste, 34127 Trieste, Italy

[#] Center for Cooperative Research in Biomaterials (CIC biomaGUNE), Basque Research and Technology Alliance (BRTA), 20014 Donostia, San Sebastián, Spain

[⊥] Basque Fdn Sci, Ikerbasque 48013 Bilbao, Spain

Email: gfilippini@units.it

TABLE OF CONTENTS

GENERAL INFORMATION S3

SYNTHESIS OF THE STARTING MATERIALS S4

OPTIMIZATION OF THE REACTION CONDITIONS S6

GENERAL PROCEDURE FOR THE PHOTOCHEMICAL PROCESS BETWEEN 2-ALLYLPHENOLS **1** AND THE RADICAL PRECURSORS. S7

CHARACTERIZATION DATA OF PRODUCTS **3.** S7

SYNTHETIC ELABORATIONS S15

DESULFONYLATION OF PRODUCT **3i.** S15

SUZUKI COUPLING S16

ALKYNE FORMATION S17

UV-VIS SPECTRA S18

STERN-VOLMER STUDIES S21

CYCLIC VOLTAMMETRY MEASUREMENTS S23

REDOX POTENTIAL OF THE EXCITED STATE OF PHENOLATE **Ia** S24

NMR SPECTRA S25

GENERAL INFORMATION

NMR spectra were recorded on Bruker 400 Avance III HD equipped with a BBI-z grad probe head 5mm and Bruker 500 Avance III equipped with a BBI-ATM-z grad probe head 5mm (^{1}H: 400 MHz, ^{13}C: 100.5 MHz, ^{19}F: 376 MHz, ^{1}H: 500 MHz, ^{13}C: 125 MHz). The chemical shifts (δ) for ^{1}H and ^{13}C are given in ppm relative to residual signals of the solvents ($CHCl_3$ @ 7.26 ppm for ^{1}H NMR, and @ 77.16 ppm for ^{13}C NMR; $CFCl_3$ @ 0.0 ppm for ^{19}F NMR spectra). Coupling constants are given in Hz. The following abbreviations are used to indicate the multiplicity: s, singlet; d, doublet; t, triplet; q, quartet; m, multiplet; br, broad signal. NMR yields were calculated by using trichloroethylene as internal standard.

Microwave synthesis was performed on a CEM Discover-SP, using 10 mL glass microwave tubes.

High-Resolution Mass Spectra (HRMS) were obtained using Bruker micrOTOF-Q (ESI-TOF).

Absorption spectroscopy studies have been performed on a Varian Cary 50 UV-Vis double beam spectrophotometer (more info at: www.varianinc.com). All the spectra were recorded at room temperature using a 10 mm path length Hellma Analytics quartz cuvettes.

All the cyclic voltammograms were recorded with a scan rate of 0.1 V/s. A typical three-electrode cell was employed, which was composed of a glassy carbon (GC) working electrode (3 mm diameter), a platinum wire as counter electrode and a saturated aqueous calomel electrode (SCE) as reference electrode. The glass electrochemical cell was kept closed with a stopper annexed to the potentiostat. Oxygen was removed by purging the solvent with high-purity Argon (Ar), introduced from a line into the cell by means of a plastic tube.

Light source at 456 nm: The Kessil lamp PR160L-456 (50W) was purchased from Kessil webpage: https://www.kessil.com/science/PR160L.php.

The photochemical reactions were carried out in borosilicate glass Schlenk tubes.

SYNTHESIS OF THE STARTING MATERIALS

Sulfones **2** were synthesized according to a reported literature procedure.[1]

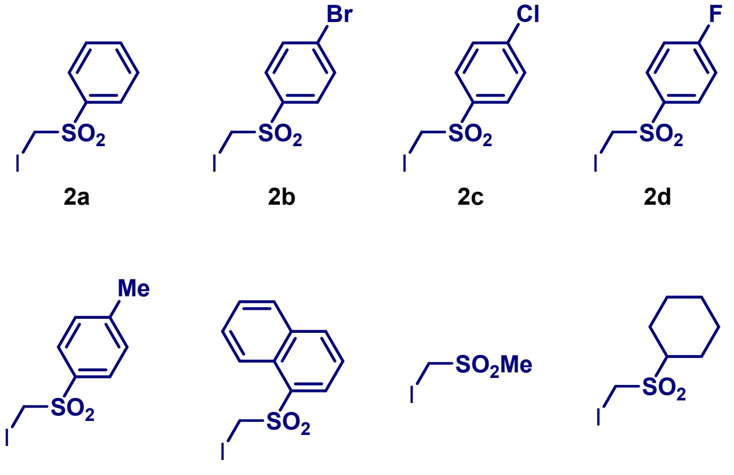

2-Allyl phenols **1** were synthesized according to a reported literature procedure.[2]

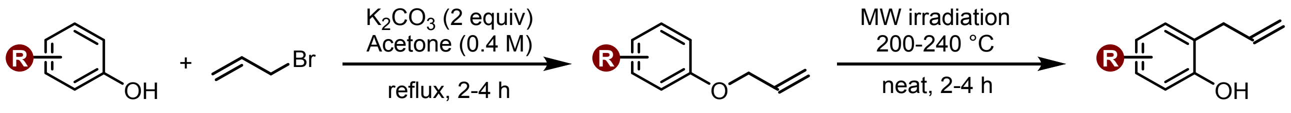

To a solution of phenols (5 mmol, 1 equiv) and potassium carbonate (10 mmol, 2 equiv) in acetone (12.5 mL) was added allyl bromide (6 mmol, 1.2 equiv). The resulting mixture was then stirred at reflux for 2-4 hours until full consumption of starting material (monitored by TLC analysis). Then, the reaction mixture was filtered through a pad of Celite and washed with ethyl acetate. The organic solvents were removed under reduced pressure to afford the crude material, which was used in the following step without furter purification.

In a microwave tube, equipped with a magnetic stir bar, were added the allyl phenyl ethers. The sealed tube was then heated at the corresponding temperature and time under microwave irradiation. for 15-30 min. After cooling to room temperature, the residue was diluted with CH_2Cl_2 and purified by FC chromatography on silica gel (EtOAc/cyclohexane mixtures) to afford the products **1.** The characterization data of the 2-allyl phenols match those reported in the literature (Table S1).

[1] Rosso, C.; Cuadros, S.; Barison, G.; Costa, P.; Kurbasic, M.; Bonchio, M.; Prato, M.; Dell'Amico, L.; Filippini, G. Unveiling the Synthetic Potential of Substituted Phenols as Fully Recyclable Organophotoredox Catalysts for the Iodosulfonylation of Olefins *ACS Catalysis* **2022**, *12*, 4290-4295.

[2] Chen, W.; Yang, X.-D.; Li, Y.; Yang, L.-J.; Wang, X.-Q.; Zhang, G.-L.; Zhang H.-B. *Org. Biomol. Chem.* **2011**, *9*, 4250-4255.

Table S1. Overview of the synthesized 2-allyl phenols **1**.

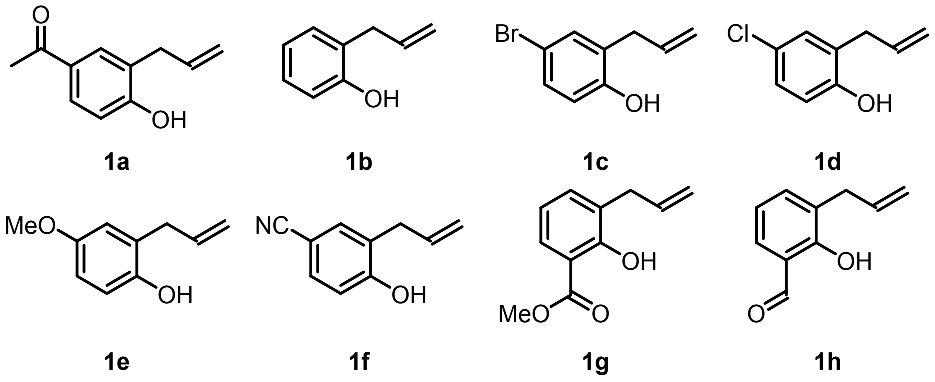

**1**	**Reference**
**1a**	Commercially available at Sigma-Aldrich
**1b**	Commercially available at Sigma-Aldrich
**1c**	*ChemCatChem* **2013**, *5*, 3309 – 3315.
**1d**	*Org. Biomol. Chem.* **2011**, *9*, 4250-4255.
**1e**	*Org. Biomol. Chem.* **2011**, *9*, 4250-4255.
**1f**	*ChemCatChem* **2013**, *5*, 3309 – 3315.
**1k**	Commercially available at Sigma-Aldrich

OPTIMIZATION OF THE REACTION CONDITIONS

Table S2.Additional results for the optimization of the reaction conditions.

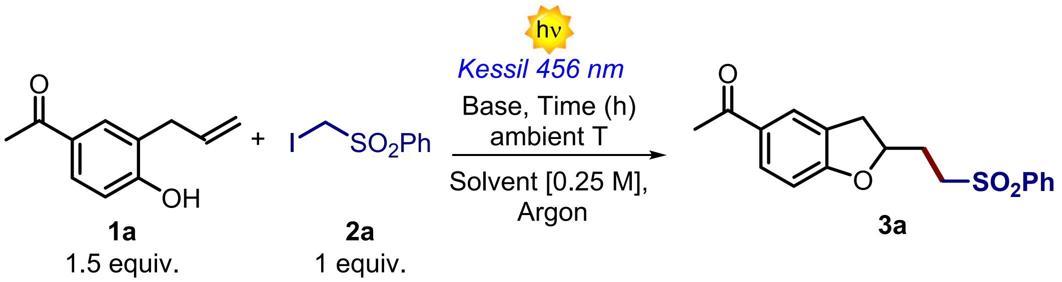

Entry	Solvent	time (h)	Base	3a (%)
1	DCM	17	TMG	57
2	DCM	2	TMG	56
3	MeCN	2	TMG	38
4	DMF	2	TMG	27
5	EtOAc	2	TMG	32
6	MeOH	2	TMG	52
7	$CHCl_3$	2	TMG	50
8	1,2-Cl_2-C_6H_4	2	TMG	64
9	PhMe	2	TMG	49
10	1,2-Cl_2-C_6H_4	2	2,6-lutidine	40
11	1,2-Cl_2-C_6H_4	2	TBD	49
12	1,2-Cl_2-C_6H_4	2	DABCO	7
13	1,2-Cl_2-C_6H_4	2	Cs_2CO_3	13

GENERAL PROCEDURE FOR THE PHOTOCHEMICAL PROCESS BETWEEN 2-ALLYLPHENOLS 1 AND THE RADICAL PRECURSORS.

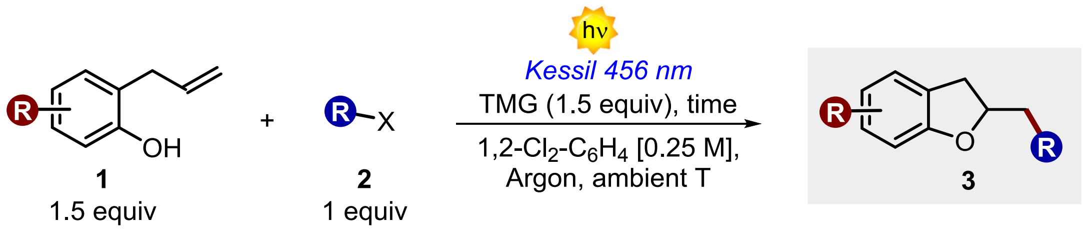

A 10 mL Schlenk tube was charged with the radical precursors **2** (0.15 mmol, 1.0 equiv), 2-allylphenols **1** (0.225 mmol, 1.5 equiv), N,N,N′,N′-tetramethylguanidine (TMG, 0.225 mmol, 1.5 equiv) and 1,2-dichlorobenzene (600 µL [**2**] = 0.25 M). The reaction mixture was thoroughly degassed via 3 cycles of freeze-pump-thaw, and the vessel was refilled with argon and placed at 4-5 cm from a Kessil lamp (λ = 456 nm). The temperature was kept at around 30°C by using a fan. Stirring was maintained for the indicated time (generally 30 min-24 hours) after which the irradiation was stopped. The reaction mixture was then quenched with an aqueous solution of HCl (5 mL, 1 M) and extracted with ethyl acetate (3 x 10 mL). The volatiles were removed in vacuo and the residue was purified by column chromatography (cyclohexane/EtOAc) to give the desired products **3**.

CHARACTERIZATION DATA OF PRODUCTS **3.**

1-(2-(2-(Phenylsulfonyl)ethyl)-2,3-dihydrobenzofuran-5-yl)ethan-1-one 3a.

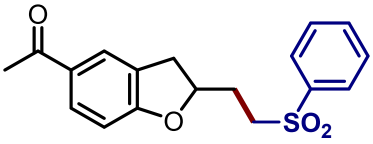

3a

Following the General Procedure applying phenol **1a** and sulfone **2a**, full conversion of **1a** was observed after 35 min. Purification by FC on silica gel (10 to 40% EtOAc/cyclohexane) afforded **3a** as an off-white solid (32.2 mg, 0.075 mmol, 65% yield).

^{1}H NMR ($CDCl_3$, 400 MHz): δ [ppm] 7.95 – 7.90 (m, 2H), 7.80 – 7.75 (m, 2H), 7.70 – 7.64 (m, 1H), 7.61 – 7.55 (m, 2H), 6.73 (d, *J*=8.2, 1H), 4.99 – 4.89 (m, 1H), 3.42 – 3.31 (m, 2H), 3.25 (ddd, *J*=14.1, 10.1, 5.7, 1H), 2.87 (dd, *J*=15.6, 6.9, 1H), 2.52 (s, 3H), 2.27 – 2.08 (m, 2H).

^{13}C{^{1}H} NMR (101 MHz, $CDCl_3$) δ 196.7, 163.2, 139.1, 134.1, 131.1, 130.7, 129.6, 128.1, 126.8, 125.8, 109.2, 82.1, 52.7, 34.7, 29.2, 26.5.

HRMS (ESI-TOF) m/z: $[M + Na]^+$ Calcd for $C_{18}H_{18}O_4SNa$ 353.0818; found: 353.0818.

1-(2-(2-((4-Bromophenyl)sulfonyl)ethyl)-2,3-dihydrobenzofuran-5-yl)ethan-1-one 3b.

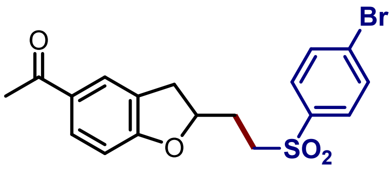

3b

Following the General Procedure applying phenol **1a** and sulfone **2b**, full conversion of **1a** was observed after 120 min. Purification by FC on silica gel (10 to 40% EtOAc/cyclohexane) afforded **3b** as an off-white solid (27.6 mg, 0.067 mmol, 45% yield).

^{1}H NMR ($CDCl_3$, 400 MHz): δ [ppm] 7.81 – 7.76 (m, 4H), 7.75 – 7.70 (m, 2H), 6.73 (d, *J*=8.2, 1H), 5.00 – 4.90 (m, 1H), 3.44 – 3.30 (m, 2H), 3.25 (ddd, *J*=14.0, 10.2, 5.6, 1H), 2.88 (dd, *J*=15.8, 7.0, 1H), 2.53 (s, 3H), 2.28 – 2.07 (m, 2H).

^{13}C{^{1}H} NMR (101 MHz, $CDCl_3$) δ 196.7, 163.1, 138.1, 132.9, 131.2, 130.7, 129.7, 129.5, 126.8, 125.8, 109.3, 82.0, 52.8, 34.8, 29.2, 26.6.

HRMS (ESI-TOF) m/z: $[M + Na]^+$ Calcd for $C_{18}H_{18}{}^{79}BrO_4SNa$ 430.9924; found: 430.9927; calcd. for $C_{18}H_{18}{}^{81}BrO_4SNa$ 432.9903; found: 432.9908.

1-(2-(2-((4-Chlorophenyl)sulfonyl)ethyl)-2,3-dihydrobenzofuran-5-yl)ethan-1-one 3c.

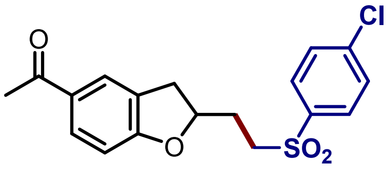

3c

Following the General Procedure applying phenol **1a** and sulfone **2c**, full conversion of **1a** was observed after 120 min. Purification by FC on silica gel (10 to 40% EtOAc/cyclohexane) afforded **3c** as an off-white solid (26.8 mg, 0.074 mmol, 49% yield).

^{1}H NMR ($CDCl_3$, 400 MHz): δ [ppm] 7.91 – 7.84 (m, 2H), 7.83 – 7.75 (m, 2H), 7.60 – 7.52 (m, 2H), 6.73 (d, *J*=8.2, 1H), 5.01 – 4.89 (m, 1H), 3.43 – 3.30 (m, 2H), 3.25 (ddd, *J*=14.1, 10.2, 5.6, 1H), 2.88 (dd, *J*=15.8, 7.0, 1H), 2.53 (s, 3H), 2.28 – 2.08 (m, 2H).

^{13}C{^{1}H} NMR (101 MHz, $CDCl_3$) δ 196.7, 163.1, 140.9, 137.5, 131.2, 130.7, 129.9, 129.7, 126.8, 125.8, 109.3, 82.0, 52.8, 34.8, 29.2, 26.6.

HRMS (ESI-TOF) m/z: $[M + Na]^+$ Calcd for $C_{18}H_{18}{}^{35}ClO_4SNa$ 387.0429; found: 387.0435; calcd. for $C_{18}H_{18}{}^{37}ClO_4SNa$ 389.0399; found: 389.0403.

1-(2-(2-((4-Fluorophenyl)sulfonyl)ethyl)-2,3-dihydrobenzofuran-5-yl)ethan-1-one 3d.

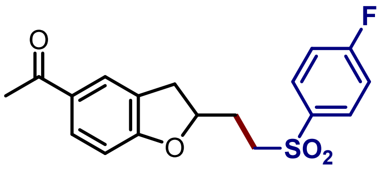

3d

Following the General Procedure applying phenol **1a** and sulfone **2d,** full conversion of **1a** was observed after 120 min. Purification by FC on silica gel (10 to 40% EtOAc/cyclohexane) afforded **3d** as an off-white solid (23.0 mg, 0.066 mmol, 44% yield).

^{1}H NMR ($CDCl_3$, 400 MHz): δ [ppm] 7.98 – 7.92 (m, 2H), 7.81 – 7.75 (m, 2H), 7.29 – 7.23 (m, 2H), 6.73 (d, *J*=8.9, 1H), 4.99 – 4.91 (m, 1H), 3.43 – 3.31 (m, 2H), 3.25 (ddd, *J*=14.0, 10.2, 5.6, 1H), 2.88 (dd, *J*=15.8, 7.0, 1H), 2.53 (s, 3H), 2.27 – 2.07 (m, 2H).

^{13}C{^{1}H} NMR (101 MHz, $CDCl_3$) δ 196.7, 167.4, 164.8, 163.2, 130.88 (d, *J*=30.1), 135.2, 135.1, 131.2, 131.1, 126.8, 125.8, 116.94 (d, *J*=22.7), 109.3, 82.0, 52.9, 34.8, 29.3, 26.5.

HRMS (ESI-TOF) m/z: $[M + Na]^+$ Calcd for $C_{18}H_{18}FO_4SNa$ 371.0724; found: 371.0724.

1-(2-(2-((4-Methylphenyl)sulfonyl)ethyl)-2,3-dihydrobenzofuran-5-yl)ethan-1-one 3e.

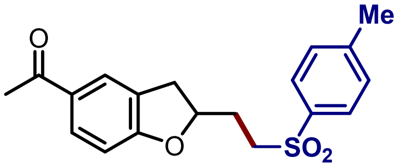

3e

Following the General Procedure applying phenol **1a** and sulfone **2e**, full conversion of **1a** was observed after 120 min. Purification by FC on silica gel (10 to 40% EtOAc/cyclohexane) afforded **3e** as an off-white solid (29.9 mg, 0.087 mmol, 58% yield).

^{1}H NMR ($CDCl_3$, 500 MHz): δ [ppm] 7.83 – 7.74 (m, 4H), 7.36 (d, *J*=8.1, 2H), 6.72 (d, *J*=8.2, 1H), 4.98 – 4.89 (m, 1H), 3.39 – 3.28 (m, 2H), 3.22 (ddd, *J*=13.9, 10.3, 5.5, 1H), 2.86 (dd, *J*=15.8, 7.1, 1H), 2.52 (s, 3H), 2.44 (s, 3H), 2.25 – 2.07 (m, 2H).

^{13}C{^{1}H} NMR (125 MHz, $CDCl_3$) δ 196.7, 163.2, 145.1, 136.1, 131.1, 130.6, 130.2, 128.2, 126.9, 125.8, 109.2, 82.2, 52.8, 34.7, 29.3, 26.5, 21.8.

HRMS (ESI-TOF) m/z: $[M + Na]^+$ Calcd for $C_{19}H_{20}O_4SNa$ 367.0975; found: 367.0976.

1-(2-(2-(Naphthalen-1-ylsulfonyl)ethyl)-2,3-dihydrobenzofuran-5-yl)ethan-1-one 3f.

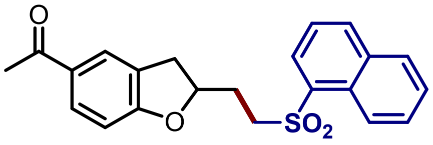

3f

Following the General Procedure applying phenol **1a** and sulfone **2f**, full conversion of **1a** was observed after 60 min. Purification by FC on silica gel (10 to 40% EtOAc/cyclohexane) afforded **3f** as an off-white solid (17.1 mg, 0.045 mmol, 30% yield).

^{1}H NMR ($CDCl_3$, 500 MHz): δ [ppm] 8.74 (d, *J*=8.7, 1H), 8.32 (dd, *J*=7.3, 1.3, 1H), 8.15 (d, *J*=8.2, 1H), 7.99 (d, *J*=7.4, 1H), 7.79 – 7.69 (m, 3H), 7.67 – 7.58 (m, 2H), 6.67 (d, *J*=8.3, 1H), 4.97 – 4.89 (m, 1H), 3.55 (ddd, *J*=14.0, 10.0, 5.5, 1H), 3.47 (ddd, *J*=14.1, 9.8, 5.8, 1H), 3.33 (dd, *J*=15.7, 9.1, 1H), 2.83 (dd, *J*=15.7, 7.0, 1H), 2.51 (s, 3H), 2.26 – 2.10 (m, 2H).

^{13}C{^{1}H} NMR (125 MHz, $CDCl_3$) δ 196.7, 163.2, 135.6, 134.4, 134.0, 131.1, 130.9, 130.7, 129.5, 129.03, 128.99, 127.3, 126.9, 125.8, 124.6, 124.1, 109.2, 82.1, 52.3, 34.7, 29.3, 26.5.

HRMS (ESI-TOF) m/z: $[M + Na]^+$ Calcd for $C_{22}H_{20}O_4SNa$ 403.0975; found: 403.0976.

1-(2-(2-(Methylsulfonyl)ethyl)-2,3-dihydrobenzofuran-5-yl)ethan-1-one 3g.

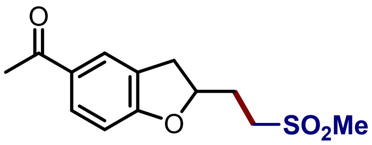

3g

Following the General Procedure applying phenol **1a** and sulfone **2g**, full conversion of **1a** was observed after 120 min. Purification by FC on silica gel (10 to 40% EtOAc/cyclohexane) afforded **3g** as an off-white solid (24.9 mg, 0.093 mmol, 62% yield).

^{1}H NMR ($CDCl_3$, 500 MHz): δ [ppm] 7.84 – 7.78 (m, 2H), 6.78 (d, *J*=8.3, 1H), 5.06 – 4.98 (m, 1H), 3.43 (dd, *J*=15.8, 9.2, 1H), 3.30 (ddd, *J*=13.8, 10.2, 5.2, 1H), 3.20 (ddd, *J*=13.8, 10.1, 5.7, 1H), 2.97 – 2.90 (m, 4H), 2.53 (s, 3H), 2.33 (dddd, *J*=14.0, 10.0, 5.7, 3.8, 1H), 2.24 (dddd, *J*=14.0, 10.0, 8.9, 5.2, 1H).

^{13}C{^{1}H} NMR (125 MHz, $CDCl_3$) δ 196.7, 163.2, 131.2, 130.7, 126.8, 125.9, 109.3, 82.0, 51.1, 41.1, 34.8, 28.8, 26.5.

HRMS (ESI-TOF) m/z: $[M + Na]^+$ Calcd for $C_{13}H_{16}O_4SNa$ 291.0662; found: 291.0661.

1-(2-(2-(Cyclohexylsulfonyl)ethyl)-2,3-dihydrobenzofuran-5-yl)ethan-1-one 3h.

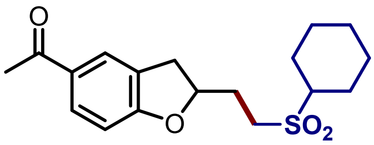

3h

Following the General Procedure applying phenol **1a** and sulfone **2h**, full conversion of **1a** was observed after 60 min. Purification by FC on silica gel (10 to 40% EtOAc/cyclohexane) afforded **3h** as an off-white solid (31.8 mg, 0.095 mmol, 63% yield).

^{1}H NMR ($CDCl_3$, 500 MHz): δ [ppm] 7.85 – 7.75 (m, 2H), 6.76 (d, *J*=8.3, 1H), 5.04 – 4.97 (m, 1H), 3.41 (dd, *J*=15.8, 9.2, 1H), 3.18 (ddd, *J*=13.4, 10.3, 5.2, 1H), 3.07 (ddd, *J*=13.4, 10.1, 5.6, 1H), 2.93 (dd, *J*=15.8, 7.0, 1H), 2.89 – 2.82 (m, 1H), 2.53 (s, 3H), 2.31 (dddd, *J*=14.1, 9.9, 5.6, 3.8, 1H), 2.27 – 2.14 (m, 3H), 1.98 – 1.89 (m, 2H), 1.79 – 1.68 (m, 1H), 1.61 – 1.50 (m, 2H), 1.36 – 1.16 (m, 3H).

^{13}C{^{1}H} NMR (125 MHz, $CDCl_3$) δ 196.7, 163.2, 131.1, 130.6, 126.9, 125.8, 109.2, 82.5, 61.6, 45.6, 34.8, 27.8, 26.5, 25.3, 25.2, 25.14, 25.12.

HRMS (ESI-TOF) m/z: $[M + Na]^+$ Calcd for $C_{18}H_{24}O_4SNa$ 359.1288; found: 359.1283.

2-(2-(Phenylsulfonyl)ethyl)-2,3-dihydrobenzofuran 3i.

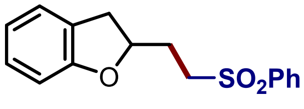

3i

This reaction was carried out on a 0.5 mmol scale of sulfone **2a**. Following the General Procedure applying phenol **1b** and sulfone **2a**, full conversion of **1b** was observed after 120 min. Purification by FC on silica gel (10 to 40% EtOAc/cyclohexane) afforded **3i** as a colorless oil (70.5 mg, 0.245 mmol, 49% yield).

^{1}H NMR ($CDCl_3$, 500 MHz): δ [ppm] = 7.70 – 7.64 (m, 1H), 7.62 – 7.56 (m, 2H), 7.15 – 7.12 (m, 1H), 7.11 – 7.06 (m, 1H), 6.85 – 6.81 (m, 1H), 6.71 (d, *J*=7.9, 1H), 4.90 – 4.79 (m, 1H), 3.41 – 3.30 (m, 3H), 3.26 (ddd, *J*=14.0, 10.5, 5.3, 1H), 2.85 (dd, *J*=15.6, 7.2, 1H), 2.24 – 2.08 (m, 3H).

^{13}C{^{1}H} NMR (125 MHz, $CDCl_3$) δ 159.0, 139.2, 134.0, 129.5, 128.3, 128.1, 126.0, 125.1, 120.8, 109.6, 80.6, 52.9, 35.4, 29.2.

HRMS (ESI-TOF) m/z: $[M + Na]^+$ Calcd for $C_{16}H_{16}O_3SNa$ 311.0712; found: 311.0713.

2-(2-(Methylsulfonyl)ethyl)-2,3-dihydrobenzofuran 3j.

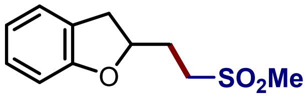

3j

Following the General Procedure applying phenol **1b** and sulfone **2g**, full conversion of **1b** was observed after 120 min. Purification by FC on silica gel (10 to 40% EtOAc/cyclohexane) afforded **3j** as an off-white solid (20.0 mg, 0.089 mmol, 59% yield).

^{1}H NMR ($CDCl_3$, 500 MHz): δ [ppm] 7.17 (d, *J*=7.4, 1H), 7.15 – 7.09 (m, 1H), 6.89 – 6.82 (m, 1H), 6.76 (d, *J*=7.7, 1H), 4.95 – 4.86 (m, 1H), 3.39 (dd, *J*=15.7, 9.1, 1H), 3.31 (ddd, *J*=13.8, 10.4, 5.3, 1H), 3.19 (ddd, *J*=14.0, 10.2, 5.7, 1H), 2.96 – 2.86 (m, 4H), 2.35 – 2.16 (m, 2H).

^{13}C{^{1}H} NMR (125 MHz, $CDCl_3$) δ 158.9, 128.4, 126.0, 125.2, 120.9, 109.6, 80.5, 51.3, 41.0, 35.4, 28.8.

HRMS (ESI-TOF) m/z: $[M + Na]^+$ Calcd for $C_{11}H_{14}O_3SNa$ 249.0556; found: 249.0560.

5-Bromo-2-(2-(methylsulfonyl)ethyl)-2,3-dihydrobenzofuran 3k.

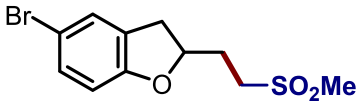

3k

Following the General Procedure applying phenol **1c** and sulfone **2g**, full conversion of **1c** was observed after 120 min. Purification by FC on silica gel (10 to 40% EtOAc/cyclohexane) afforded **3k** as an off-white solid (22.4 mg, 0.074 mmol, 49% yield).

^{1}H NMR ($CDCl_3$, 500 MHz): δ [ppm] 7.30 – 7.24 (m, 1H), 7.24 – 7.18 (m, 1H), 6.63 (d, *J*=8.4, 1H), 4.97 – 4.87 (m, 1H), 3.38 (dd, *J*=15.9, 9.4, 1H), 3.28 (ddd, *J*=13.8, 10.3, 5.2, 1H), 3.18 (ddd, *J*=13.9, 10.2, 5.7, 1H), 2.94 (s, 3H), 2.89 (dd, *J*=15.9, 7.0, 1H), 2.32 – 2.16 (m, 2H).

^{13}C{^{1}H} NMR (125 MHz, $CDCl_3$) δ 158.2, 131.2, 128.5, 128.2, 112.7, 111.2, 81.3, 51.2, 41.1, 35.3, 28.7.

HRMS (ESI-TOF) m/z: $[M + Na]^+$ Calcd for $C_{11}H_{13}{}^{79}BrO_3SNa$ 326.9661; found: 326.9660; calcd. for $C_{11}H_{13}{}^{81}BrO_3S$+Na 328.9641; found: 328.9650.

5-Chloro-2-(2-(methylsulfonyl)ethyl)-2,3-dihydrobenzofuran 3l.

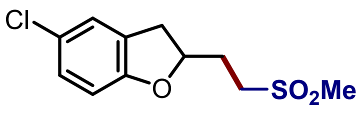

3l

Following the General Procedure applying phenol **1d** and sulfone **2g**, full conversion of **1d** was observed after 120 min. Purification by FC on silica gel (10 to 40% EtOAc/cyclohexane) afforded **3l** as an off-white solid (22.4 mg, 0.074 mmol, 49% yield).

^{1}H NMR ($CDCl_3$, 500 MHz): δ [ppm] 7.14 – 7.11 (m, 1H), 7.09 – 7.05 (m, 1H), 6.66 (d, *J*=8.5, 1H), 4.98 – 4.89 (m, 1H), 3.37 (dd, *J*=15.9, 9.2, 1H), 3.29 (ddd, *J*=13.8, 10.3, 5.2, 1H), 3.18 (ddd, *J*=13.9, 10.2, 5.7, 1H), 2.94 (s, 3H), 2.89 (dd, *J*=15.9, 7.0, 1H), 2.34 – 2.23 (m, 1H), 2.24 – 2.16 (m, 1H).

^{13}C{^{1}H} NMR (125 MHz, $CDCl_3$) δ 157.6, 128.3, 128.0, 125.6, 125.3, 110.5, 81.3, 51.2, 41.1, 35.4, 28.7.

HRMS (ESI-TOF) m/z: [M + Na]$^+$ Calcd for $C_{11}H_{13}{}^{35}ClO_3SNa$ 283.0167; found: 283.0166; calcd. for $C_{11}H_{13}{}^{37}ClO_4S$+Na 285.0137; found: 285.0135.

5-Methoxy-2-(2-(methylsulfonyl)ethyl)-2,3-dihydrobenzofuran 3m.

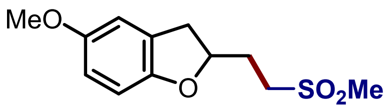

3m

Following the General Procedure applying phenol **1e** and sulfone **2g**, full conversion of **1e** was observed after 360 min. Purification by FC on silica gel (10 to 40% EtOAc/cyclohexane) afforded **3m** as an off-white solid (11.9 mg, 0.047 mmol, 31% yield).

^{1}H NMR ($CDCl_3$, 500 MHz): δ [ppm] 6.77 – 6.74 (m, 1H), 6.66 – 6.65 (m, 2H), 4.92 – 4.84 (m, 1H), 3.75 (s, 3H), 3.36 (dd, *J*=15.7, 9.0, 1H), 3.30 (ddd, *J*=13.8, 10.4, 5.2, 1H), 3.22 – 3.14 (m, 1H), 2.94 (s, 3H), 2.88 (dd, *J*=15.8, 7.0, 1H), 2.33 – 2.16 (m, 2H).

^{13}C{^{1}H} NMR (125 MHz, $CDCl_3$) δ 154.5, 153.1, 127.0, 113.3, 111.5, 109.5, 80.7, 56.2, 51.3, 41.0, 35.9, 28.8.

HRMS (ESI-TOF) m/z: [M + Na]$^+$ Calcd for $C_{12}H_{16}O_4SNa$ 279.0662; found: 279.0663.

2-(2-(Methylsulfonyl)ethyl)-2,3-dihydrobenzofuran-5-carbonitrile 3n.

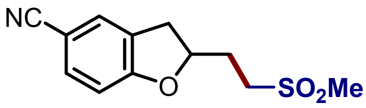

3n

Following the General Procedure applying phenol **1f** and sulfone **2g**, full conversion of **1f** was observed after 120 min. Purification by FC on silica gel (10 to 40% EtOAc/cyclohexane) afforded **3n** as an off-white solid (26.0 mg, 0.10 mmol, 69% yield).

^{1}H NMR ($CDCl_3$, 500 MHz): δ [ppm] 7.47 – 7.43 (m, 2H), 6.83 – 6.79 (m, 1H), 5.08 – 4.99 (m, 1H), 3.43 (dd, *J*=16.0, 9.2, 1H), 3.29 (ddd, *J*=13.9, 10.1, 5.3, 1H), 3.20 (ddd, *J*=13.8, 9.9, 5.9, 1H), 2.97 – 2.89 (m, 4H), 2.33 (dddd, *J*=13.9, 9.9, 5.9, 3.8, 1H), 2.28 – 2.18 (m, 1H).

^{13}C{^{1}H} NMR (125 MHz, $CDCl_3$) δ 162.5, 133.9, 129.2, 127.8, 119.4, 110.6, 104.3, 82.1, 51.0, 41.2, 34.7, 28.6.

HRMS (ESI-TOF) m/z: [M + Na]$^+$ Calcd for $C_{12}H_{13}NO_3SNa$ 274.0509; found: 274.0507.

Methyl 2-(2-(methylsulfonyl)ethyl)-2,3-dihydrobenzofuran-7-carboxylate 3o.

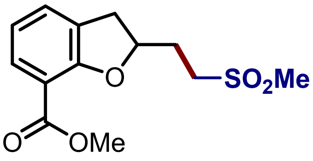

3o

Following the General Procedure applying phenol **1g** and sulfone **2g**, full conversion of **1g** was observed after 120 min. Purification by FC on silica gel (10 to 50% EtOAc/cyclohexane and then re-purified 10 to 20% EtOAc/CH_2Cl_2) afforded **3o** as an off-white solid (17.6 mg, 0.062 mmol, 41% yield).

^{1}H NMR ($CDCl_3$, 500 MHz): δ [ppm] 7.73 (d, *J*=7.9, 1H), 7.33 (dd, *J*=7.3, 1.3, 1H), 6.92 – 6.87 (m, 1H), 5.11 – 5.03 (m, 1H), 3.89 (s, 3H), 3.42 (dd, *J*=15.8, 9.2, 1H), 3.35 (ddd, *J*=13.9, 10.4, 5.2, 1H), 3.24 (ddd, *J*=13.9, 10.2, 5.6, 1H), 2.96 (s, 3H), 2.92 (ddt, *J*=15.8, 6.5, 1.1, 1H), 2.38 – 2.20 (m, 2H).

^{13}C{^{1}H} NMR (125 MHz, $CDCl_3$) δ 165.6, 159.4, 130.2, 129.7, 128.4, 120.7, 113.4, 81.8, 52.0, 51.2, 41.1, 34.7, 28.8.

HRMS (ESI-TOF) m/z: $[M + Na]^+$ Calcd for $C_{13}H_{16}O_5SNa$ 307.0611; found: 3070612.

2-(2-(Methylsulfonyl)ethyl)-2,3-dihydrobenzofuran-7-carbaldehyde 3p.

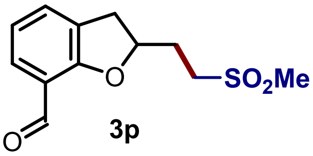

3p

Following the General Procedure applying phenol **1h** and sulfone **2g**, full conversion of **1h** was observed after 30 min. Purification by FC on silica gel (10 to 40% EtOAc/cyclohexane) afforded **3p** as an off-white solid (11.1 mg, 0.044 mmol, 29% yield).

^{1}H NMR ($CDCl_3$, 500 MHz): δ [ppm] 10.18 (s, 1H), 7.60 (d, *J*=7.8, 1H), 7.43 – 7.35 (m, 1H), 6.96 (t, *J*=7.5, 1H), 5.16 – 5.07 (m, 1H), 3.43 (dd, *J*=15.9, 9.2, 1H), 3.34 (ddd, *J*=13.8, 10.2, 5.3, 1H), 3.25 (ddd, *J*=13.8, 10.0, 5.7, 1H), 3.00 – 2.91 (m, 4H), 2.37 (dddd, *J*=14.1, 9.9, 5.7, 3.9, 1H), 2.33 – 2.24 (m, 1H).

^{13}C{^{1}H} NMR (125 MHz, $CDCl_3$) δ 188.7, 160.9, 131.2, 128.5, 127.8, 121.3, 119.9, 82.8, 51.1, 41.2, 34.4, 28.6.

HRMS (ESI-TOF) m/z: $[M + Na]^+$ Calcd for $C_{12}H_{14}O_4SNa$ 277.0505; found: 277.0504.

1-(2-(7,7,7,7,7,7,7,7,7,7,7,7,7-Tridecafluoro-7l16-hepta-2,4,6-triyn-1-yl)-2,3-dihydrobenzofuran-5-yl)ethan-1-one 3q.

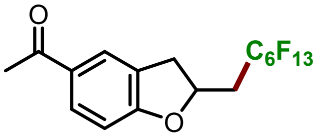

Following the General Procedure applying phenol **1a** and perfluorohexyl iodide, the reaction was stirred for 16 h. Purification by FC on silica gel (10% EtOAc/cyclohexane) afforded **3q** as a white solid (30.0 mg, 0.060 mmol, 40% yield).

3q

^{1}H NMR ($CDCl_3$, 500 MHz): δ [ppm] 7.86 – 7.80 (m, 2H), 6.83 (d, *J*=8.3, 1H), 5.29 – 5.21 (m, 1H), 3.52 (dd, *J*=15.9, 9.1, 1H), 3.05 (dd, *J*=15.9, 7.5, 1H), 2.80 – 2.66 (m, 1H), 2.56 – 2.41 (m, 4H).

^{19}F NMR ($CDCl_3$, 376 MHz): δ [ppm] 80.73 – -80.82 (m, 3F), -112.37 – -112.79 (m, 2F), -121.61 – -121.97 (m, 2F), -122.72 – -122.96 (m, 2F), -123.37 – -123.66 (m, 2F), -125.99 – -126.22 (m, 2F).

^{13}C{^{1}H} NMR (125 MHz, $CDCl_3$) δ 196.7, 163.0, 131.5, 130.9, 126.5, 125.7, 109.5, 37.2 (t, *J*=21.3), 35.7, 26.6.

HRMS (ESI-TOF) m/z: $[M + Na]^+$ Calcd for $C_{17}H_{11}F_{13}O_2Na$ 517.0444; found: 517.0444.

2-(7,7,7,7,7,7,7,7,7,7,7,7,7-Tridecafluoro-7l16-hepta-2,4,6-triyn-1-yl)-2,3-dihydrobenzofuran-5-carbonitrile 3r.

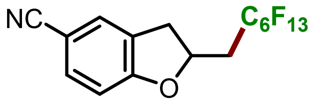

3r

Following the General Procedure applying phenol **1a** and perfluorohexyl iodide, the reaction was stirred for 240 min. Purification by FC on silica gel (10% EtOAc/cyclohexane) afforded **3r** as a white solid (26.5 mg, 0.056 mmol, 37% yield).

^{1}H NMR ($CDCl_3$, 500 MHz): δ [ppm] 7.52 – 7.45 (m, 2H), 6.86 (d, *J*=7.9, 1H), 5.30 – 5.22 (m, 1H), 3.52 (dd, *J*=16.1, 9.1, 1H), 3.06 (dd, *J*=16.1, 7.7, 1H), 2.83 – 2.65 (m, 1H), 2.59 – 2.38 (m, 1H).

^{19}F NMR ($CDCl_3$, 376 MHz): δ [ppm] -80.64 – -80.81 (m, 3F), -111.21 – -111.50 (m, 1F), -112.25 – -112.67 (m, 1F), -121.52 – -121.89 (m, 2F), -122.53 – -123.01 (m, 2F), -123.24 – -123.57 (m, 2F), -125.98 – -126.14 (m, 2F).

^{13}C{^{1}H} NMR (125 MHz, $CDCl_3$) δ 162.4, 134.1, 129.1, 127.4, 119.4, 110.8, 104.6, 77.3, 37.1 (t, *J*=21.0), 35.6.

HRMS (ESI-TOF) m/z: $[M + Na]^+$ Calcd for $C_{16}H_8F_{13}NONa$ 500.0291; found: 500.0290.

1-(2-(2,2-Dibromovinyl)-2,3-dihydrobenzofuran-5-yl)ethan-1-one 3s.

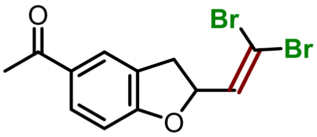

3s

Following the General Procedure applying phenol **1a** and tetrabromomethane, the reaction was stirred for 240 min. Purification by FC on silica gel (10% EtOAc/cyclohexane) afforded **3s** as a white solid (18.2 mg, 0.053 mmol, 35% yield).

^{1}H NMR ($CDCl_3$, 500 MHz): 7.85 – 7.79 (m, 2H), 6.82 (d, *J*=8.3, 1H), 6.71 (d, *J*=8.0, 1H), 5.48 (ddd, *J*=9.4, 7.9, 7.0, 1H), 3.55 (dd, *J*=15.8, 9.4, 1H), 3.05 (dd, *J*=15.8, 7.0, 1H), 2.54 (s, 3H).

13C{1H} NMR (125 MHz, $CDCl_3$) δ 196.7, 163.1, 137.4, 131.4, 130.8, 126.7, 125.7, 109.4, 93.7, 83.4, 34.8, 26.6.

HRMS (ESI-TOF) m/z: $[M + Na]^+$ Calcd for $C_{12}H_{10}{}^{79}Br^{79}BrO_2Na$ 366.8940; found: 366.8943; calcd. for $C_{12}H_{10}{}^{79}Br^{81}BrO_2Na$ 368.8920; found: 368.8924; calcd. for $C_{12}H_{10}{}^{81}Br^{81}BrO_2Na$ 370.8899; found: 370.8906

1-(2-(2,2-Dichlorovinyl)-2,3-dihydrobenzofuran-5-yl)ethan-1-one 3t.

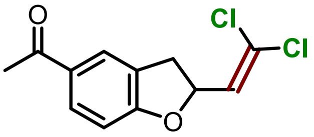

3t

Following the General Procedure applying phenol **1a** and bromotrichloromethane, the reaction was stirred for 240 min. Purification by FC on silica gel (10% EtOAc/cyclohexane) afforded **3t** as a white solid (15.4 mg, 0.06 mmol, 40% yield).

^{1}H NMR ($CDCl_3$, 500 MHz): δ [ppm] 7.87 – 7.77 (m, 2H), 6.82 (d, *J*=8.3, 1H), 6.15 (d, *J*=8.3, 1H), 5.60 (ddd, *J*=9.3, 8.3, 7.2, 1H), 3.54 (dd, *J*=15.9, 9.4, 1H), 3.04 (dd, *J*=16.0, 7.2, 1H), 2.54 (s, 3H).

^{13}C{^{1}H} NMR (125 MHz, $CDCl_3$) δ 196.7, 163.1, 131.4, 130.8, 128.9, 126.8, 125.7, 125.3, 109.4, 80.8, 35.1, 26.6.

HRMS (ESI-TOF) m/z: $[M + Na]^+$ Calcd for $C_{12}H_{10}{}^{35}Cl^{35}ClO_2Na$ 278.9951; found: 278.9951; calcd. for $C_{12}H_{10}{}^{35}Cl^{37}ClO_2Na$ 280.9921; found: 280.9919; calcd. for $C_{12}H_{10}{}^{37}Cl^{37}ClO_2Na$ 282.9892; found: 282.9888.

1-(3-((1,1-Dioxidothiochroman-4-yl)methyl)-4-hydroxyphenyl)ethan-1-one 3aa.

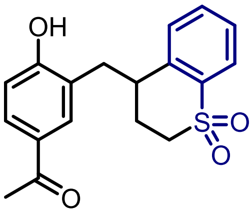

Purification by FC on silica gel (10 to 40% EtOAc/cyclohexane) afforded the side product as a white solid in less than 10% yield

^{1}H NMR (DMSO-d_6, 500 MHz): δ [ppm] 10.56 (br s, 1H), 7.85 (d, *J*=2.2, 1H), 7.80 (dd, *J*=7.9, 1.3, 1H), 7.75 (dd, *J*=8.4, 2.3, 1H), 7.64 – 7.59 (m, 1H), 7.56 (d, *J*=7.3, 1H), 7.52 – 7.48 (m, 1H), 6.94 (d, *J*=8.5, 1H), 3.75 (ddd, *J*=14.6, 12.0, 2.8, 1H), 3.48 – 3.35 (m, 2H), 3.04 (dd, *J*=13.4, 4.5, 1H), 2.89 (dd, *J*=13.4, 10.9, 1H), 2.48 (s, 3H), 2.34 – 2.24 (m, 1H), 2.04 – 1.96 (m, 1H).

^{13}C{^{1}H} NMR (DMSO-d_6, 125 MHz) δ 196.2, 160.2, 140.5, 138.2, 132.4, 131.9, 129.9, 129.0, 128.6, 127.7, 125.5, 122.8, 114.9, 46.1, 36.2, 35.7, 26.3, 23.5.

HRMS (ESI-TOF) m/z: [M + H]$^+$ Calcd for $C_{18}H_{18}O_4SH$ 331.1004; found: 331.0992.

SYNTHETIC ELABORATIONS

DESULFONYLATION OF PRODUCT **3i**.

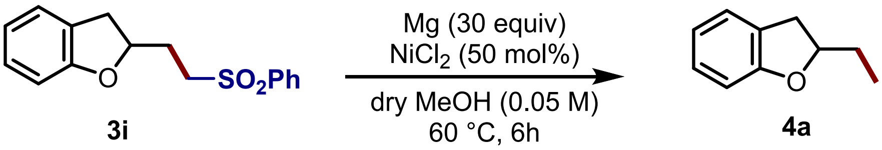

According to a modified literature procedure:[3] To a round-bottom flask containing freshly activated* Mg (30 equiv, 11.25 mmol, 273 mg) under Ar, it was added a solution of **3i** (1 equiv, 0.375 mmol, 108 mg) in dry MeOH (6.0 mL) followed by anhydrous $NiCl_2$ (0.5 equiv, 0.188 mmol, 24 mg). The mixture was stirred vigorously at 60 °C for 6 h, in an oil bath. The reaction was quenched by adding an aqueous solution of HCl (1 M). The crude mixture was then transferred to a separatory funnel and extracted with CH_2Cl_2 (3 times). The organic phases were combined and dried over Mg_2SO_4 before concentration in vacuo. The residue was purified by flash chromatography (50% CH_2Cl_2 in petroleum ether) to give **4a**.

*Activated by grinding it with mortar and pestle.

2-Ethyl-2,3-dihydrobenzofuran 4a.

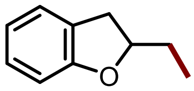

4a

Following the procedure for the desulfonylation applying product **3i** (0.375 mmol), the reaction was stirred for 5 h. After work up, **4a** was obtained as a colorless oil (22.7 mg, 0.154 mmol, 41% yield).

^{1}H NMR ($CDCl_3$, 400 MHz): δ [ppm] 7.16 (d, *J*=7.3, 1H), 7.13 – 7.07 (m, 1H), 6.85 – 6.79 (m, 1H), 6.76 (d, *J*=8.0, 1H), 4.77 – 4.66 (m, 1H), 3.27 (dd, *J*=15.5, 8.9, 1H), 2.87 (dd, *J*=15.6, 7.8, 1H), 1.92 – 1.80 (m, 1H), 1.79 – 1.66 (m, 1H), 1.04 (t, *J*=7.4, 3H).

^{13}C{^{1}H} NMR (101 MHz, $CDCl_3$) δ 159.8, 128.0, 127.1, 125.0, 120.2, 109.3, 84.7, 35.1, 29.1, 9.8.

HRMS (ESI-TOF) m/z: $[M + Na]^+$ Calcd for $C_{10}H_{12}ONa$ 149.0961; found: 149.0961.

[3] Das, I.; Pathak, T. Desulfonylation with Mg-MeOH-$NiBr_2$: An Expedient Reagent System for the Synthesis of 2-Amino-2,3-Dideoxy Furanosides. *Org. Lett.* **2006**, *8*, 1303–1306.

SUZUKI COUPLING

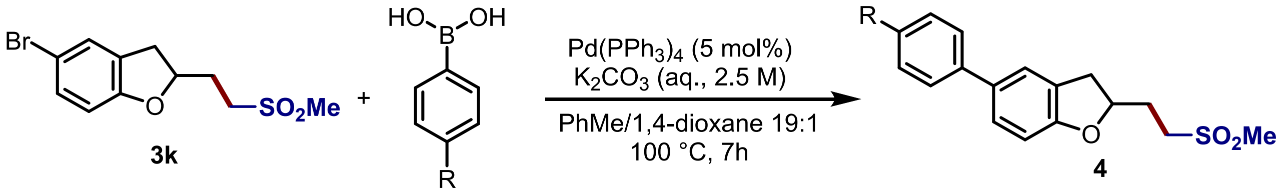

In a small Schlenk tube equipped with a magnetic stirring bar, compound **3k** (38 mg, 1.0 equiv, 0.12 mmol), toluene (450 μL), 1,4-dioxane (50 μL), the corresponding arylboronic acid (2.5 equiv, 0.30 mmol) and K_2CO_3 (2M aqueous solution, 150 μL, 2.5 equiv, 0.30 mmol) were added in this order. The whole reaction mixture was then degassed using the freezing-pump method (3 times) and $Pd(PPh_3)_4$ (7.0 mg, 5 mol%) was added. The resulting biphasic mixture was vigorously stirred under argon atmosphere at 100 °C, in an oil bath, for 7 h and then cooled to room temperature. The crude mixture was passed through a short plug of SiO_2 eluted with CH_2Cl_2 (10 mL) and EtOAc (3x5 mL), evaporated in vacuo and purified by column chromatography on silica gel (EtOAc/cyclohexane mixtures) to afford the desired products **4**.

2-(2-(Methylsulfonyl)ethyl)-5-phenyl-2,3-dihydrobenzofuran 4b.

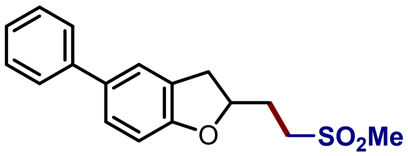

4b

Substrate **3k** and phenylboronic acid were reacted for 7 h following the procedure for the Suzuki-Miyaura coupling. Purification by FC on silica gel (10 to 50% EtOAc/cyclohexane) followed by another FC on silica gel (1 to 5% Et_2O/CH_2Cl_2) afforded **4b** as a white solid (19.6 mg, 0.065 mmol, 54% yield).

^{1}H NMR ($CDCl_3$, 500 MHz): δ [ppm] 7.53 – 7.49 (m, 2H), 7.45 – 7.39 (m, 3H), 7.38 – 7.34 (m, 1H), 7.33 – 7.27 (m, 1H), 6.82 (d, *J*=8.3, 1H), 5.03 – 4.92 (m, 1H), 3.46 (dd, *J*=15.7, 9.1, 1H), 3.38 – 3.29 (m, 1H), 3.27 – 3.18 (m, 1H), 3.01 – 2.93 (m, 4H), 2.40 – 2.19 (m, 2H).

^{13}C{^{1}H} NMR (125 MHz, $CDCl_3$) δ 158.6, 141.2, 134.7, 128.9, 127.6, 126.9, 126.8, 126.7, 124.1, 109.8, 81.1, 51.3, 41.1, 35.5, 28.9.

HRMS (ESI-TOF) m/z: $[M + Na]^+$ Calcd for $C_{17}H_{18}O_3SNa$ 325.0869; found: 325.0867.

2-(2-(Methylsulfonyl)ethyl)-5-phenyl-2,3-dihydrobenzofuran 4c.

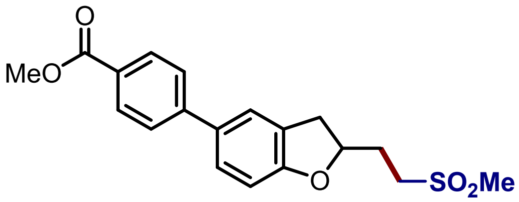

4c

Substrate **3k** and 4-methoxycarbonylphenylboronic acid were reacted for 7 h following the procedure for the Suzuki-Miyaura coupling. Purification by FC on silica gel (10 to 50% EtOAc/cyclohexane) followed by another FC on silica gel (1 to 5% Et_2O/CH_2Cl_2) afforded **4c** as a white solid (20.7 mg, 0.06 mmol, 48% yield).

^{1}H NMR ($CDCl_3$, 500 MHz): δ [ppm] 8.10 – 8.05 (m, 2H), 7.61 – 7.56 (m, 2H), 7.46 – 7.43 (m, 1H), 7.42 – 7.38 (m, 1H), 6.84 (d, J=8.3, 1H), 5.04 – 4.95 (m, 1H), 3.93 (s, 3H), 3.47 (dd, J=15.8, 9.1, 1H), 3.38 – 3.29 (m, 1H), 3.27 – 3.18 (m, 1H), 3.03 – 2.93 (m, 4H), 2.39 – 2.21 (m, 2H).

^{13}C{^{1}H} NMR (125 MHz, $CDCl_3$) δ 167.2, 159.4, 145.6, 133.3, 130.3, 128.4, 127.9, 127.1, 126.7, 124.2, 110.0, 81.3, 52.2, 51.3, 41.1, 35.4, 28.8.

HRMS (ESI-TOF) m/z: $[M + Na]^+$ Calcd for $C_{19}H_{20}O_5SNa$ 383.0924; found: 383.0921.

ALKYNE FORMATION

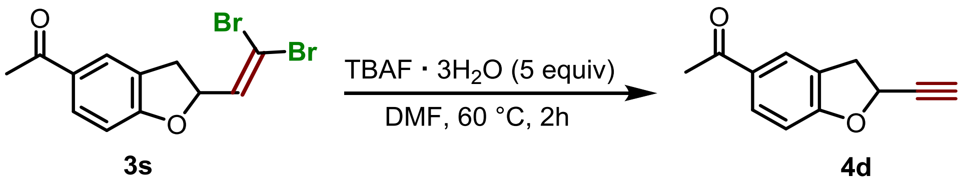

According to a reported literature procedure:[4] Bromoalkene (**3s**, 64 mg, 0.185 mmol) was dissolved in 0.925 mL of DMF (0.2 M). TBAF·$3H_2O$ (0.291 g, 0.925 mmol) was added to the solution and the reaction mixture was heated at 60 °C, in an oil bath, for 2 h (TLC). The reaction mixture was cooled to room temperature and diluted with diethyl ether (10 mL). The organic phase was washed with water and brine, dried over anhydrous $MgSO_4$, filtered, and concentrated under reduced pressure. The residue was purified by flash chromatography (5 to 10% ethyl acetate in cyclohexane) to give **4d** (15.8 mg, 46%) as a colorless oil.

^{1}H NMR ($CDCl_3$, 500 MHz): δ [ppm] 7.86 – 7.80 (m, 2H), 6.85 (d, *J*=8.1, 1H), 5.44 (ddd, *J*=9.6, 7.0, 2.2, 1H), 3.56 (dd, *J*=15.5, 9.6, 1H), 3.35 (dd, *J*=15.6, 7.0, 1H), 2.64 (d, *J*=2.2, 1H), 2.54 (s, 3H).

^{13}C{^{1}H} NMR (125 MHz, $CDCl_3$) δ 196.7, 162.7, 131.5, 130.8, 126.4, 125.6, 109.6, 81.6, 75.3, 72.5, 37.0, 26.6.

HRMS (ESI-TOF) m/z: $[M + Na]^+$ Calcd for $C_{12}H_{10}O_2Na$ 209.0573; found: 209.0574.

[4] Conversion of Bromoalkenes into Alkynes by Wet Tetra-n-butylammonium Fluoride Okutani, M.; Mori Y. *J. Org. Chem.* **2009**, *74*, 442-444

UV-VIS SPECTRA

As upon mixing of phenolate **Ia** and CBr_4 an intense color change was observed, we decided to investigate the possible formation of an EDA complex in the reaction mixture. The photo-active aggregate, formed by complexation of **Ia** and CBr_4, initiates the photochemical cascade reaction.

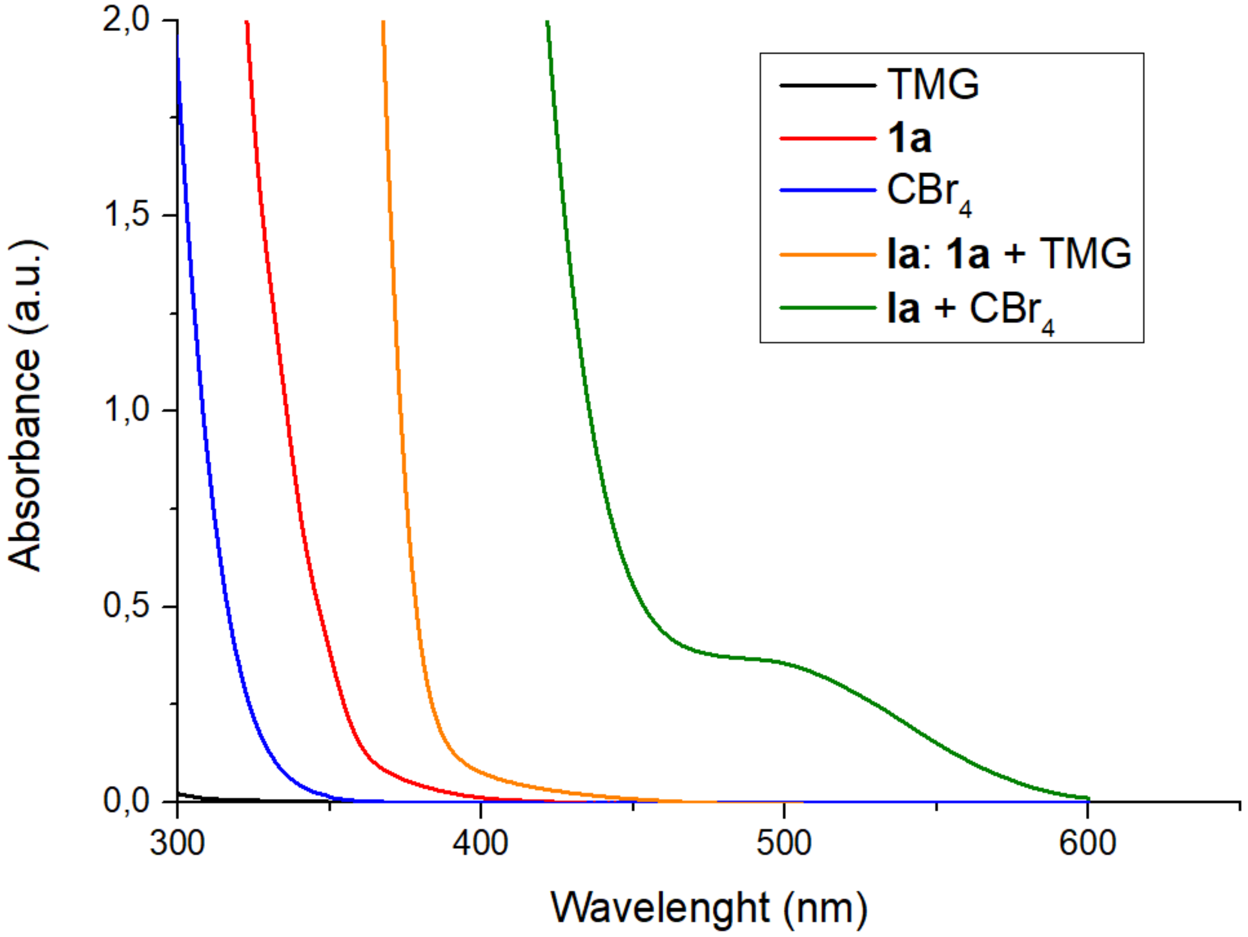

Figure S1. Optical absorption spectra recorded in 1,2-dichlorobenzene in quartz cuvettes (1 cm path): [**1a**] = 0.01 M (red line); [CBr_4] = 0.01 M (blue line); [TMG] = 0.01 M (black line); [**Ia**] = 0.01 M (orange line); reaction mixture [**Ia** + CBr_4]: 0.01 M (green line).

As upon mixing of phenolate **Ia** and $CBrCl_3$ an intense color change was observed, we decided to investigate the possible formation of an EDA complex in the reaction mixture. The photo-active aggregate, formed by complexation of **Ia** and CBr_3Cl, initiates the photochemical cascade reaction.

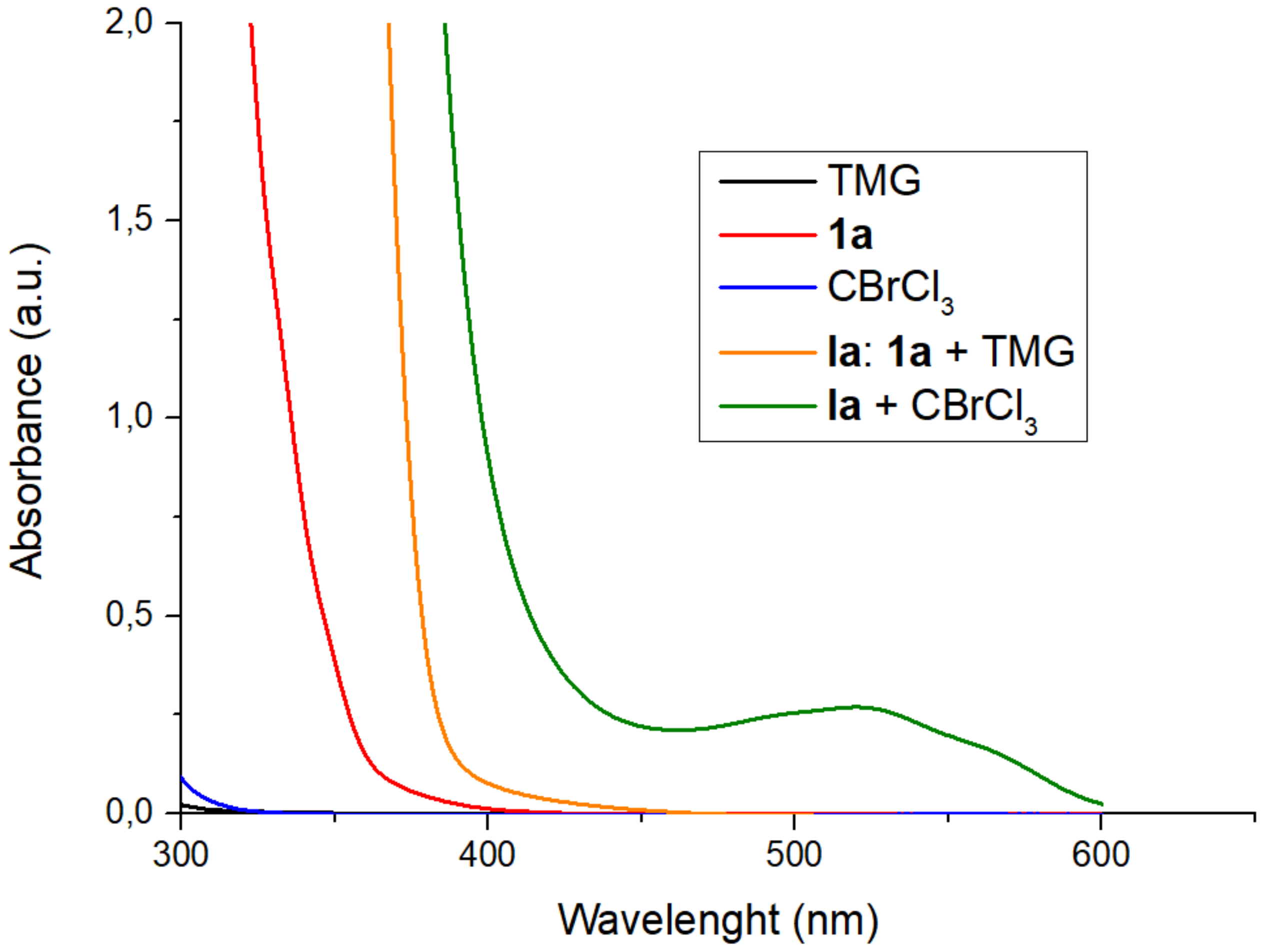

Figure S2. Optical absorption spectra recorded in 1,2-dichlorobenzene in quartz cuvettes (1 cm path): [**1a**] = 0.01 M (red line); [$CBrCl_3$] = 0.01 M (blue line); [TMG] = 0.01 M (black line); [**Ia**] = 0.01 M (orange line); reaction mixture [**Ia** + $CBrCl_3$]: 0.01 M (green line).

We decided to investigate the possible formation of an EDA complex in the reaction mixture between perfluorohexyl iodide **2i** and phenolate **Ia**. The photo-active aggregate, formed by complexation of **Ia** and **2i**, initiates the photochemical cascade reaction.

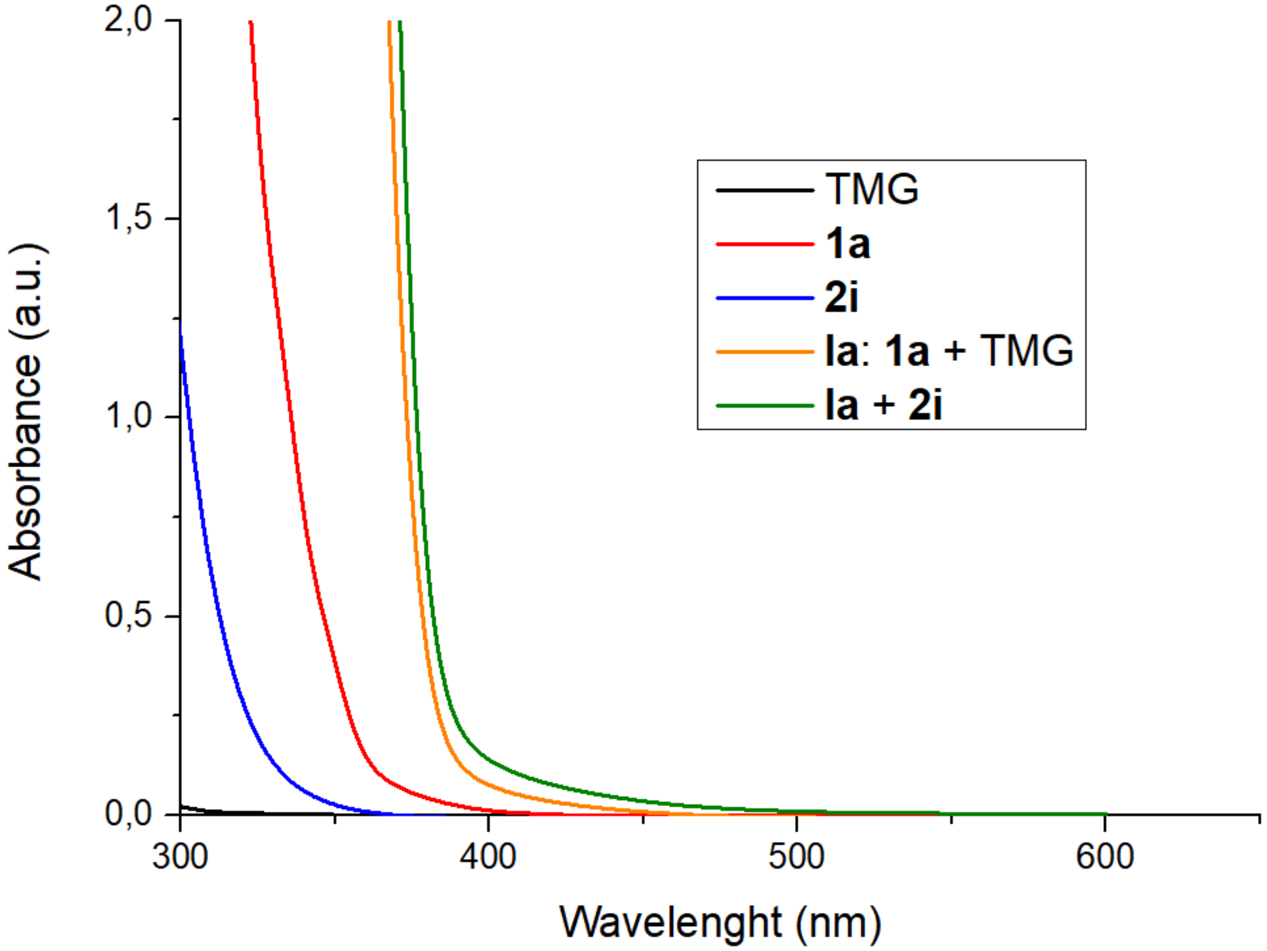

Figure S3. Optical absorption spectra recorded in 1,2-dichlorobenzene in quartz cuvettes (1 cm path): [**1a**] = 0.01 M (red line); [**2i**] = 0.01 M (blue line); [TMG] = 0.01 M (black line); [**Ia**] = 0.01 M (orange line); reaction mixture [**Ia** + **2i**]: 0.01 M (green line).

STERN-VOLMER STUDIES

To demonstrate the feasibility of a SET between phenolate **Ia** and α-iodo sulfone **2a**, a series of Stern-Volmer quenching studies were performed in 1,2-dichlorobenzene. The emission spectrum, obtained upon excitation at 400 nm, showed a decreased intensity when **2a** was added and this fluorescence quenching turned out to be linear in the range 5 ÷ 104 x 10^{-3} M, with a Stern-Volmer constant of 3.43 x 10^{-3} M^{-1} (Figure S4 and Figure S5).

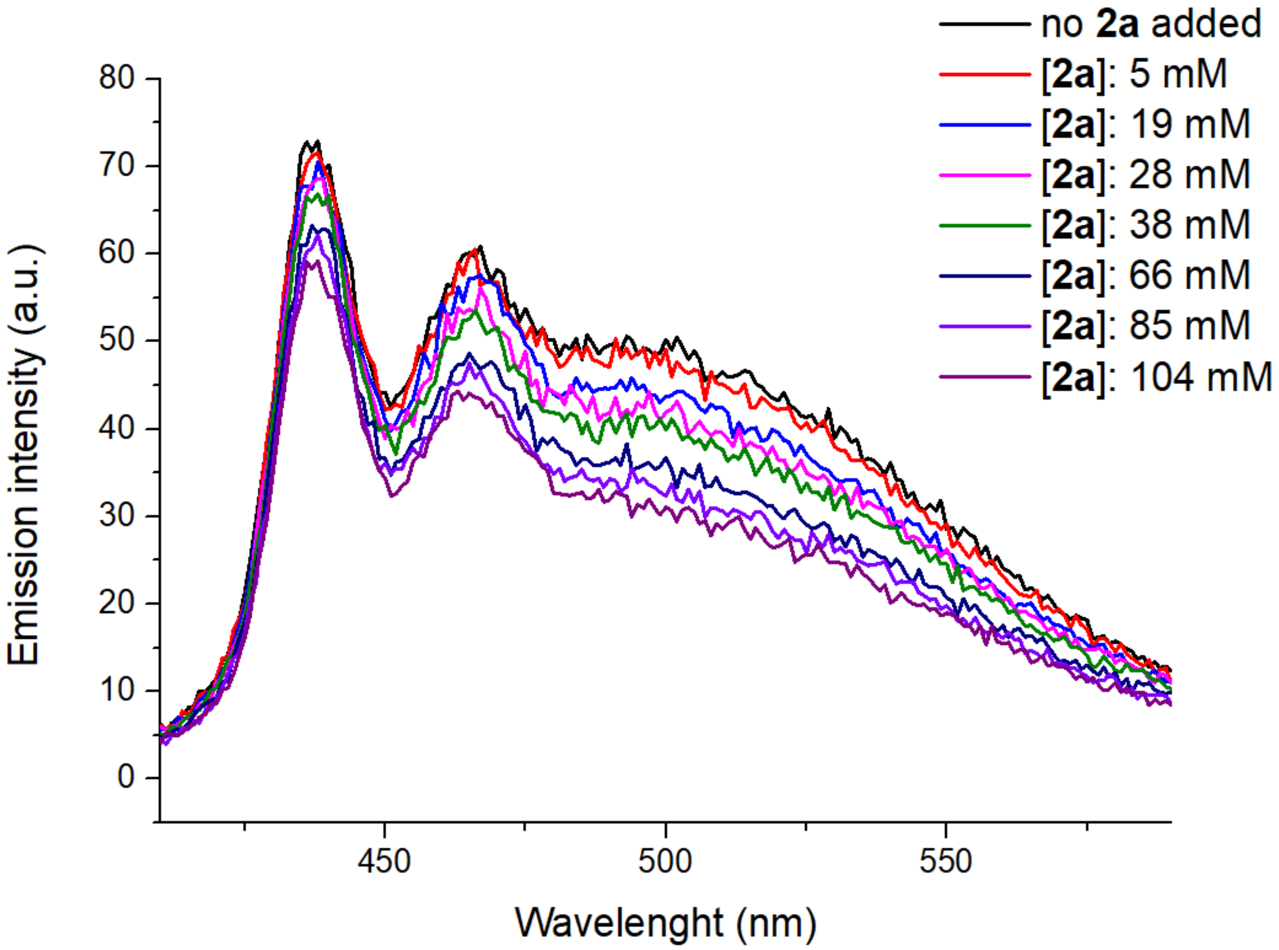

Figure S4. Emission spectra of phenolate **Ia** (black line), obtained by mixing phenol **1a** and TMG, and various mixtures of **Ia** and **2a** at increasing concentration of **2a** (values in brackets within the legend). Recorded in 1,2-dichlorobenzene in quartz cuvettes (1 cm path). [**1a**] = [TMG] = 0.01 M. [**2a**] = 5 ÷ 104 x 10^{-3} M.

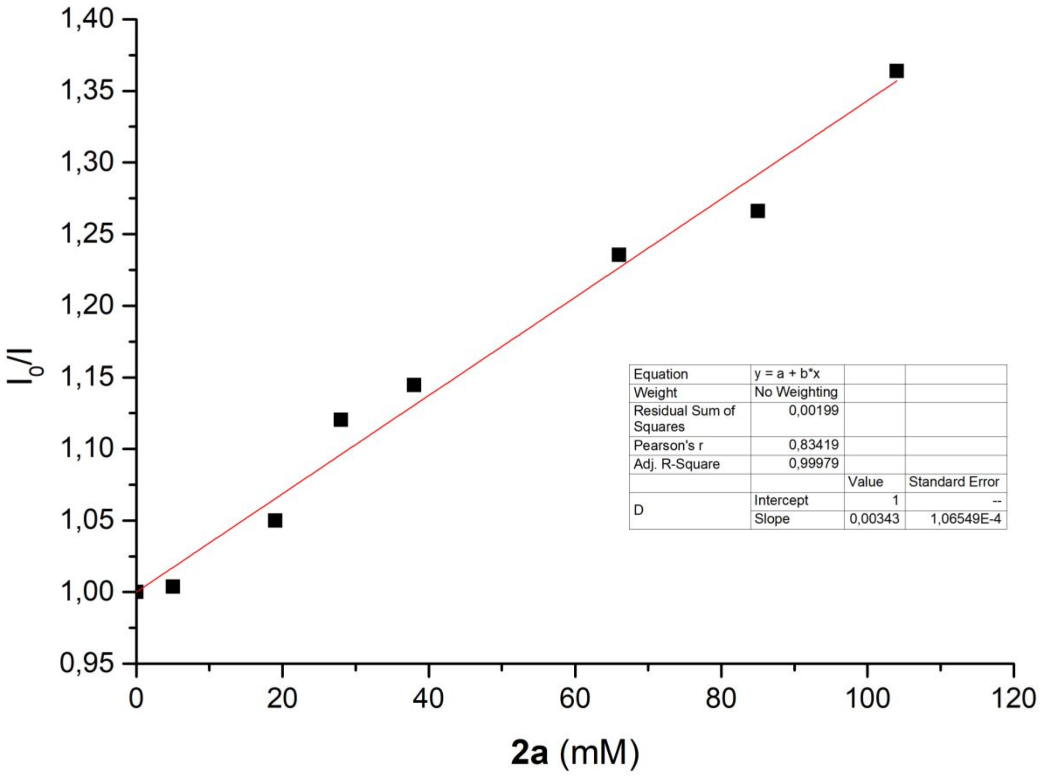

Figure S5. Stern-Volmer quenching study.

CYCLIC VOLTAMMETRY MEASUREMENTS

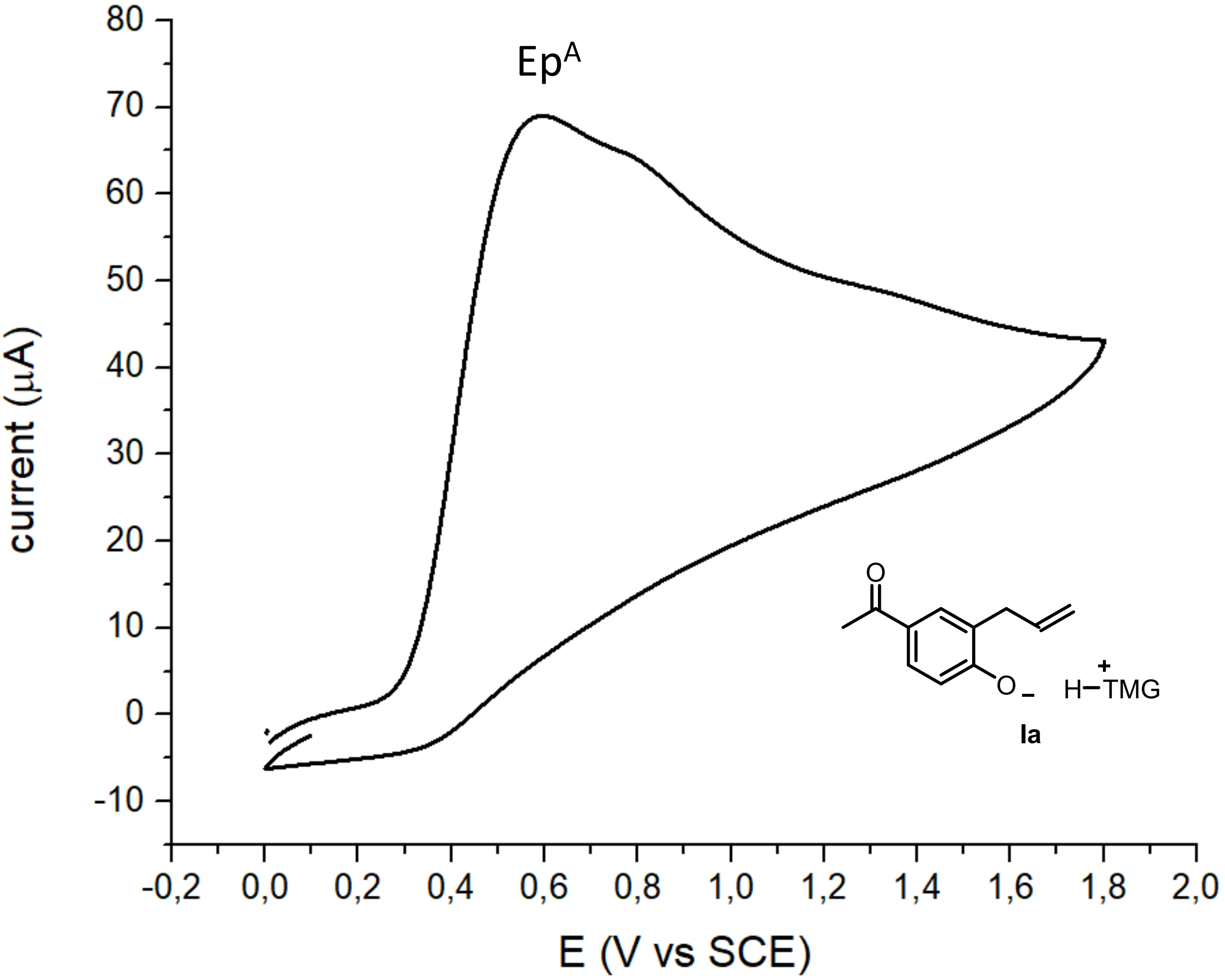

Figure S6. Cyclic voltammogram (IUPAC convention) at room temperature for the phenolate **1a** (5 mM), formed upon deprotonation of the phenol **1a** (5 mM) with TMG (5 mM); in CH_3CN using $TBAPF_6$ (0.1 M) as electrolyte. Scan rate: 0.1 V/s, start potential: 0.0 V, scan direction: from 0.0 V to 1.8 V. Working electrode: glassy carbon; reference electrode: standard calomel electrode (SCE); counter electrode: platinum wire. Irreversible oxidation, $Ep^A \approx E_{ox}$ (**Ia**$^{\bullet+}$/**Ia**) = +0.55 V *vs* SCE. Ep^A refers to the anodic peak potential, while the E_{ox} value describes the electrochemical properties of **Ia**.

REDOX POTENTIAL OF THE EXCITED STATE OF PHENOLATE Ia

The redox potential of the excited phenolate $E^0_{\boldsymbol{Ia}^*}$ was estimated by means of the Rehm-Weller equation:[5]

$$E^0_{\boldsymbol{Ia}^*} = E^0_{\boldsymbol{Ia}} - E^{00}$$

where the redox potential of the ground state phenolate ($E^0_{\boldsymbol{Ia}}$) was determined by cyclic voltammetry measurements (+0.55 V *vs* SCE, Figure S6). The excitation energy (E^{00}) of phenolate **Ia** was determined from the cross point between the absorption and the emission profile (362 nm, that corresponds to 3.42 eV, Figure S7).[6] As a result, $E^0_{\boldsymbol{Ia}^*}$ turned out to be -2.87 V *vs* SCE. Thus, a single electron transfer (SET) between the excited phenolate **Ia** and iodo sulfone **2a** could take place, according to the redox potential of this radical source (-1.4 V *vs* SCE).[7]

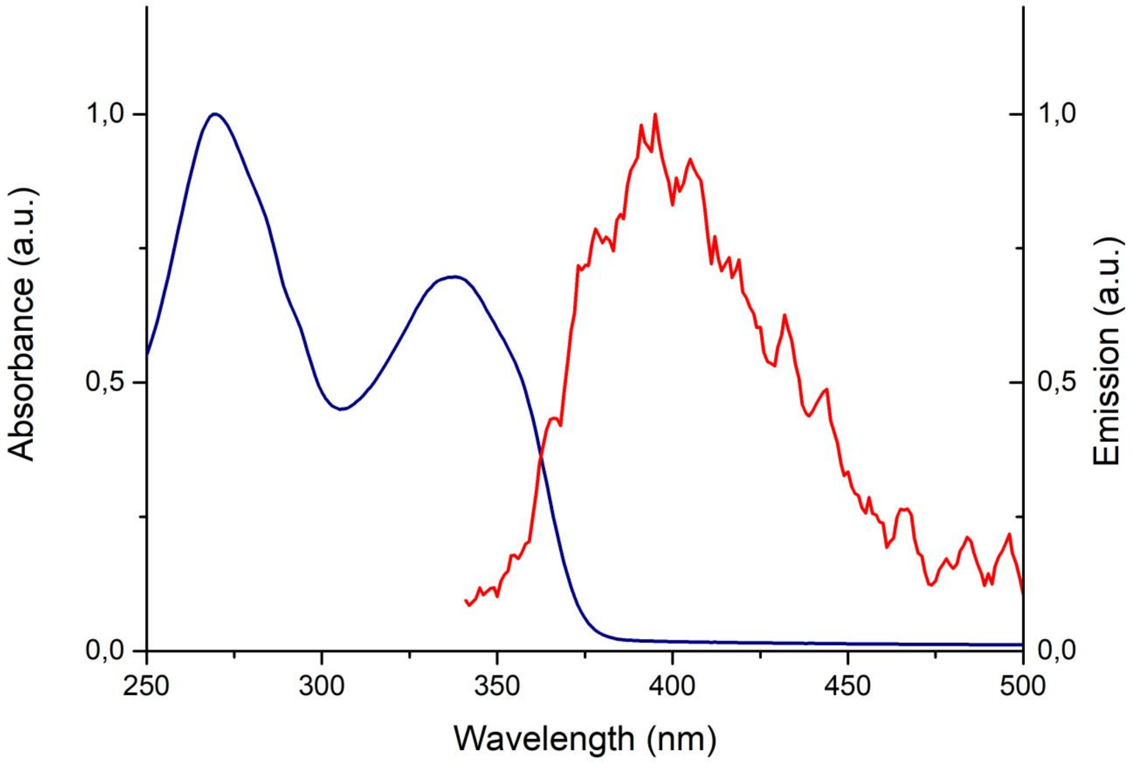

Figure S7. Normalized absorption and emission spectra.

[5] Rehm, D.; Weller, A. Kinetics of Fluorescence Quenching by Electron and H-Atom Transfer. *Isr. J. Chem.* **1970**, *8*, 259–271.

[6] Buzzetti, L.; Crisenza, G. E. M.; Melchiorre, P. Mechanistic Studies in Photocatalysis. *Angew. Chem. Int. Ed.* **2019**, *58*, 3730–3747.

[7] Filippini, G.; Silvi, M.; Melchiorre, P. Enantioselective Formal α-Methylation and α-Benzylation of Aldehydes by Means of Photo-Organocatalysis. *Angew. Chem. Int. Ed.* **2017**, *56*, 4447–4451.

NMR SPECTRA

3a - ^{1}H NMR ($CDCl_3$).

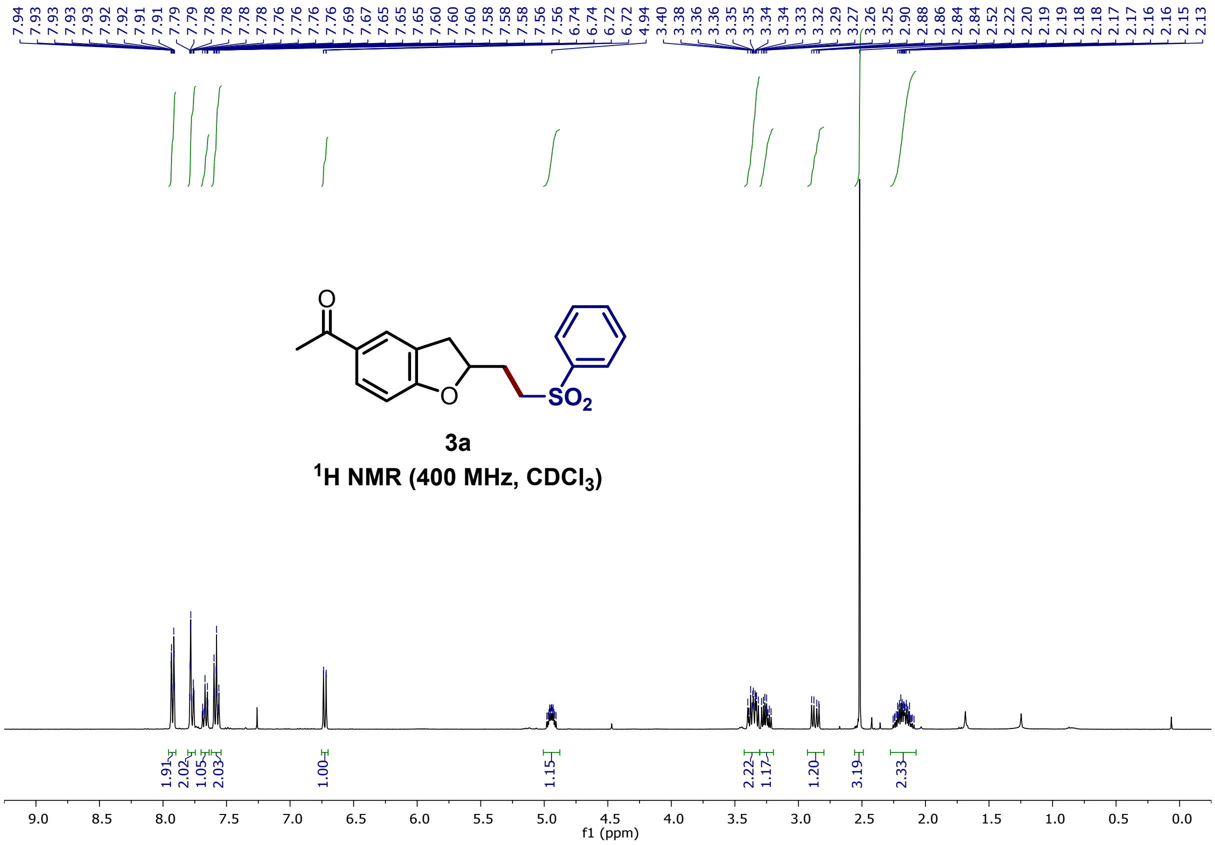

3a - $^{13}C\{^1H\}$ NMR ($CDCl_3$).

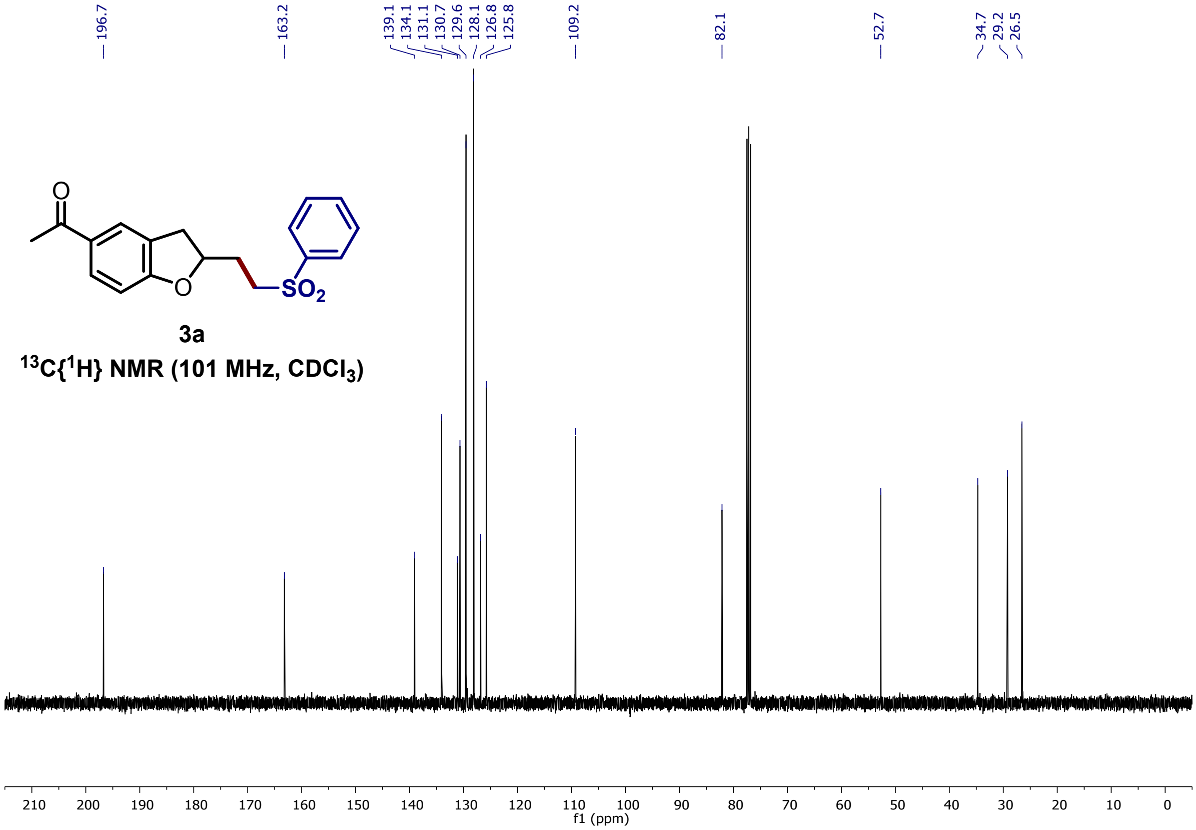

3b - ^{1}H NMR ($CDCl_3$).

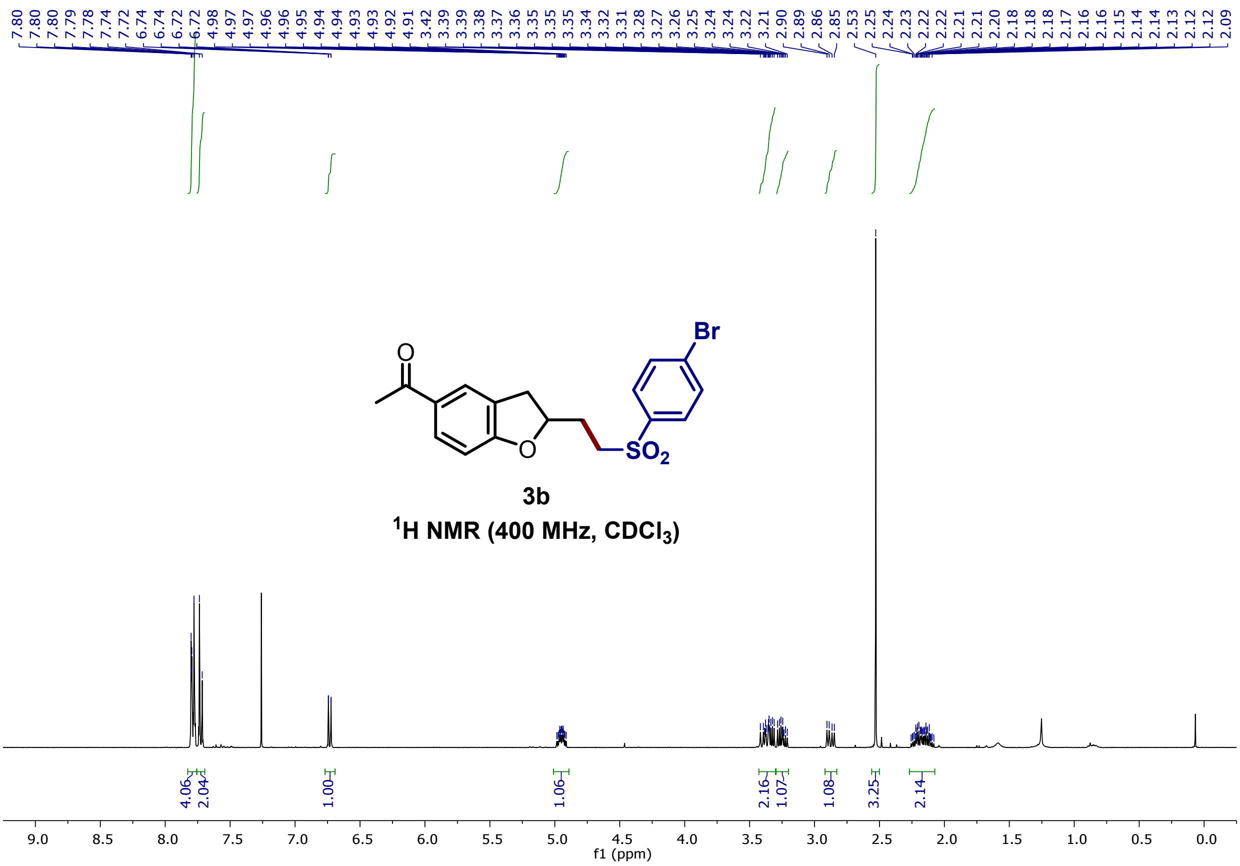

3b - $^{13}C\{^{1}H\}$ NMR ($CDCl_3$).

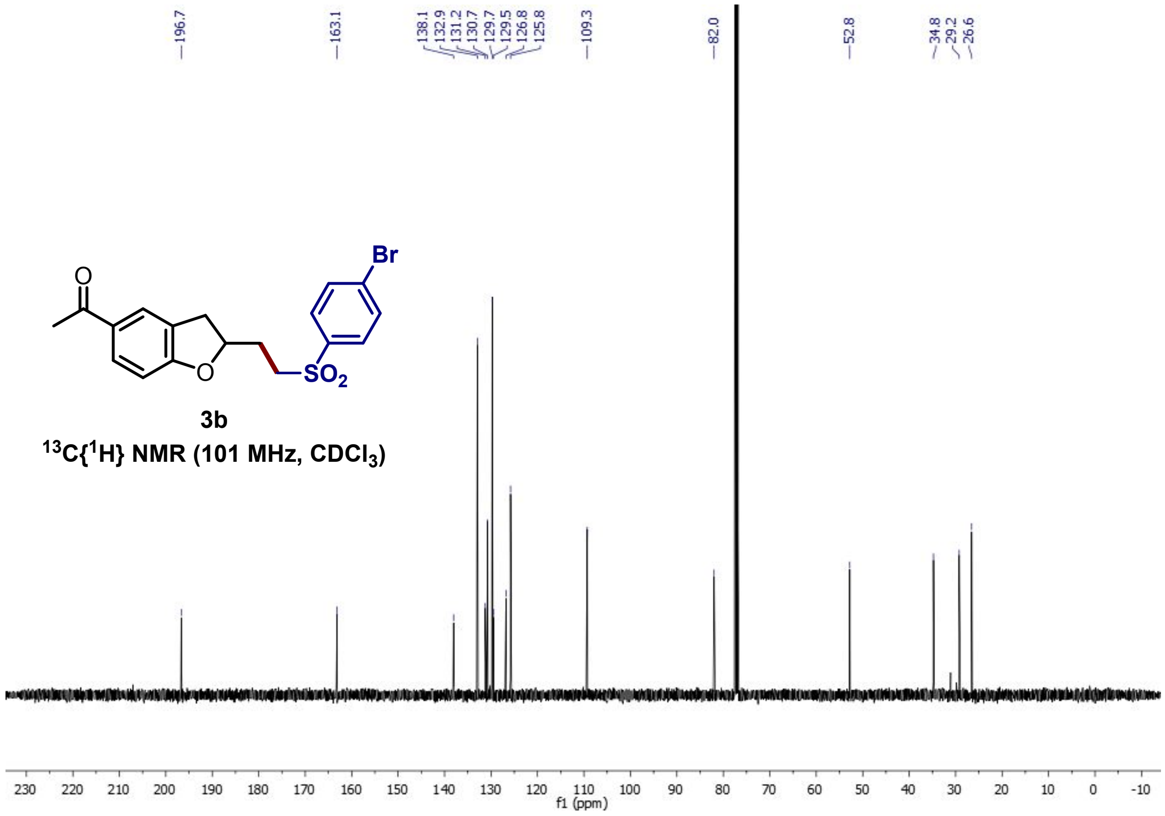

3c - ^{1}H NMR ($CDCl_3$).

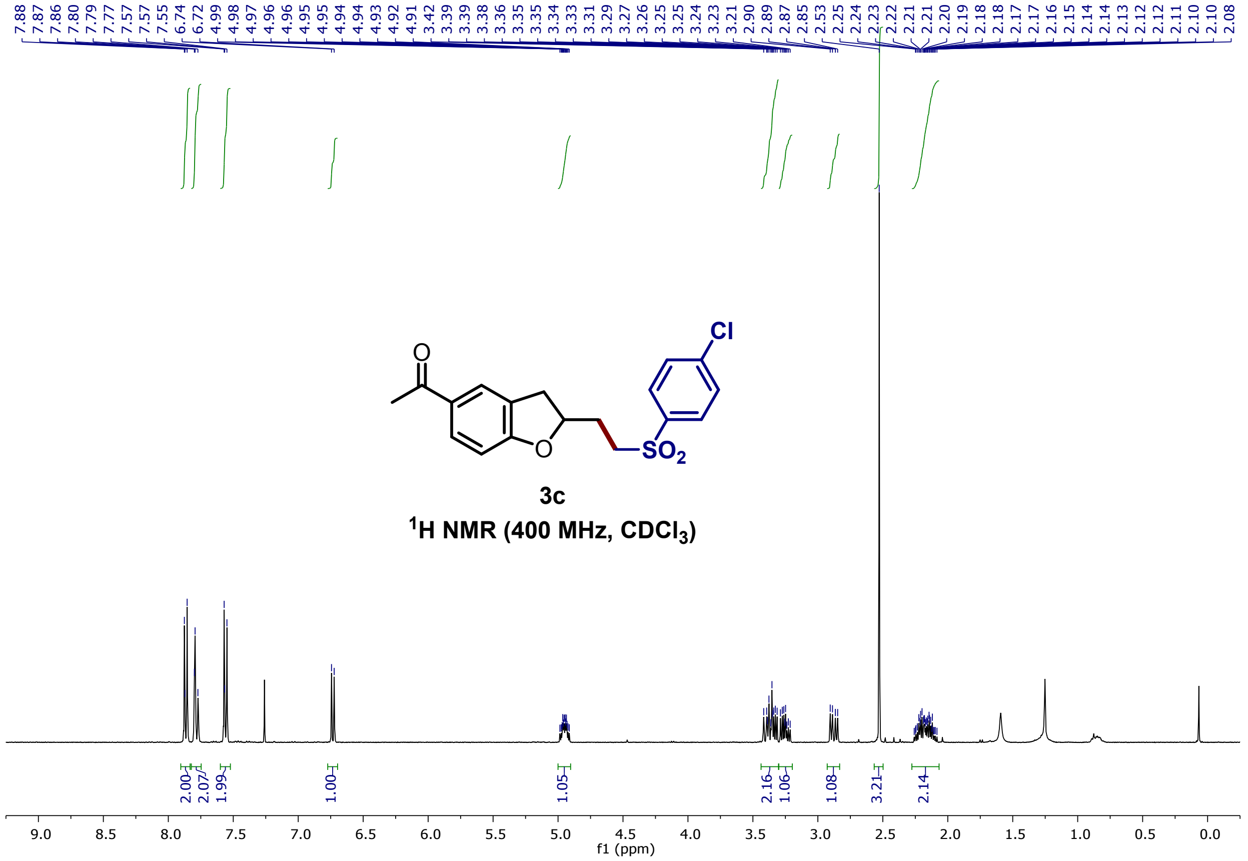

3c - $^{13}C\{^{1}H\}$ NMR ($CDCl_3$).

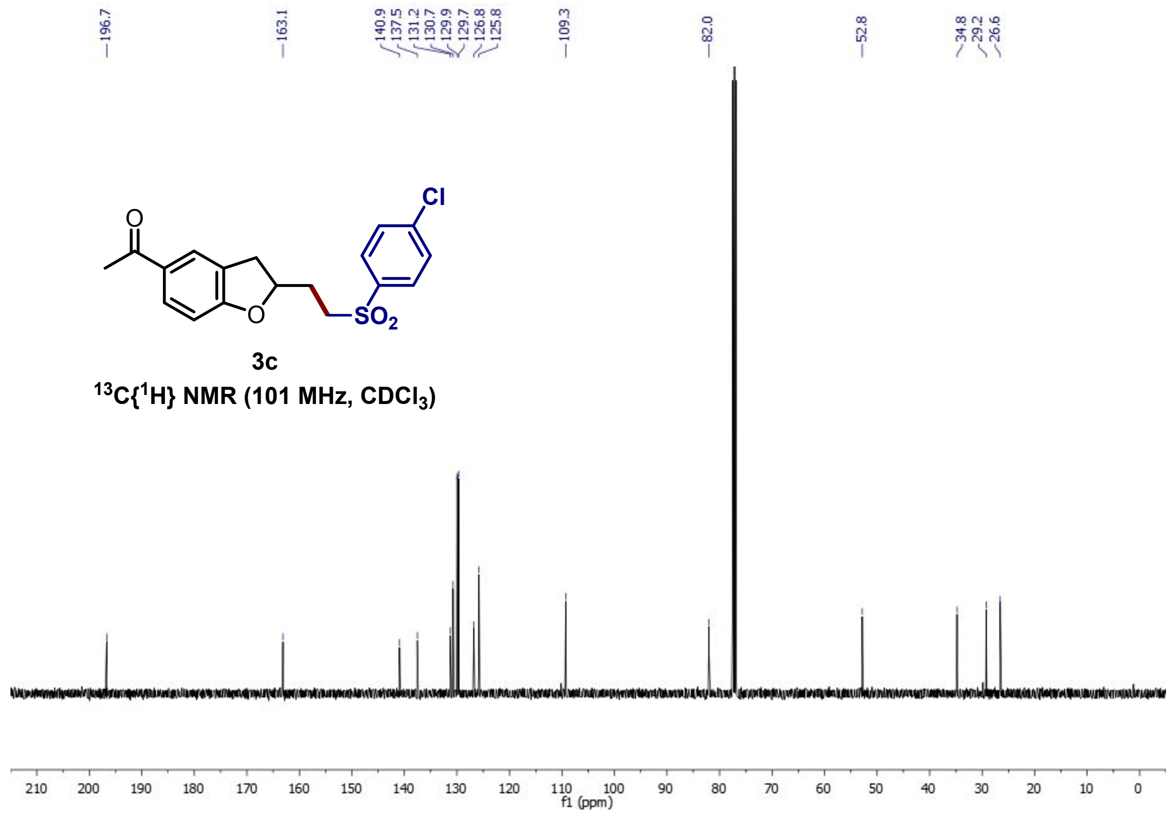

3d - ^{1}H NMR ($CDCl_3$).

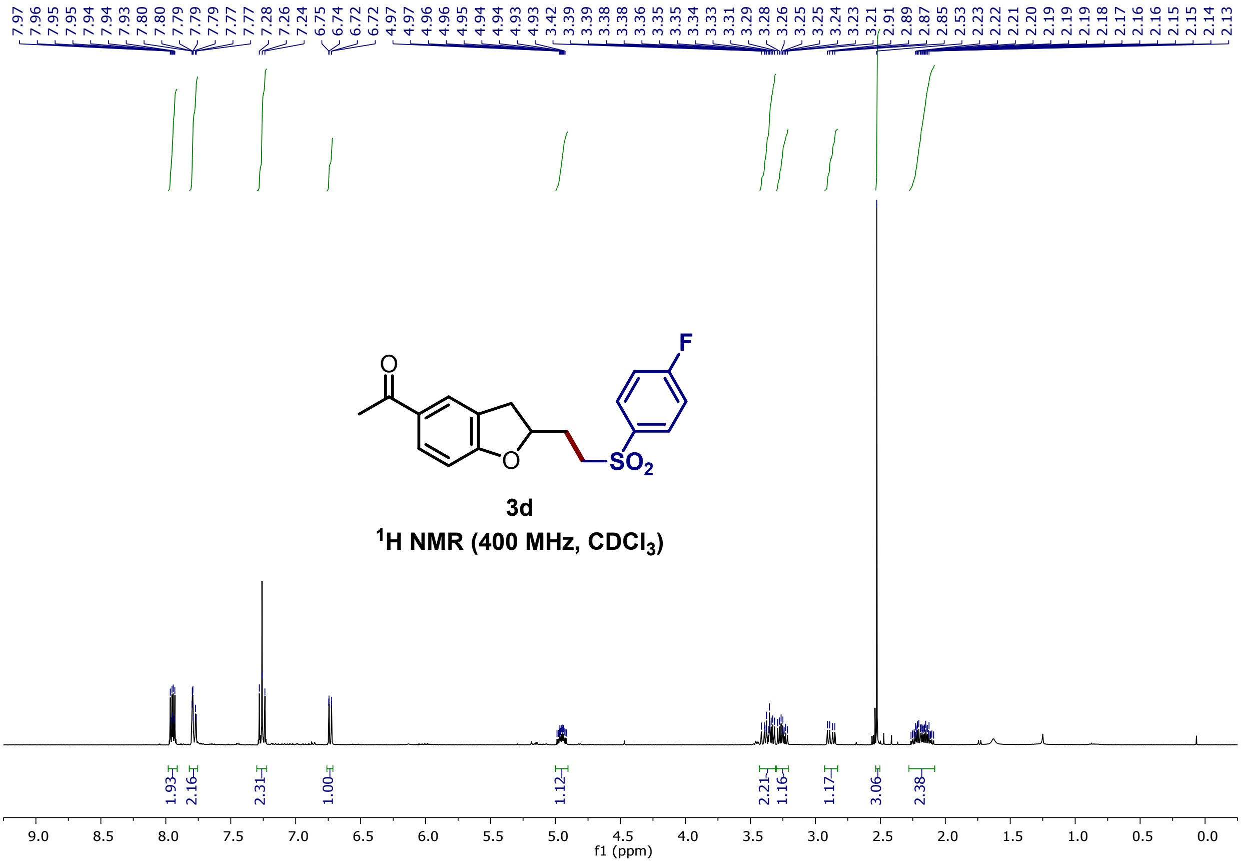

3d - $^{13}C\{^{1}H\}$ NMR ($CDCl_3$).

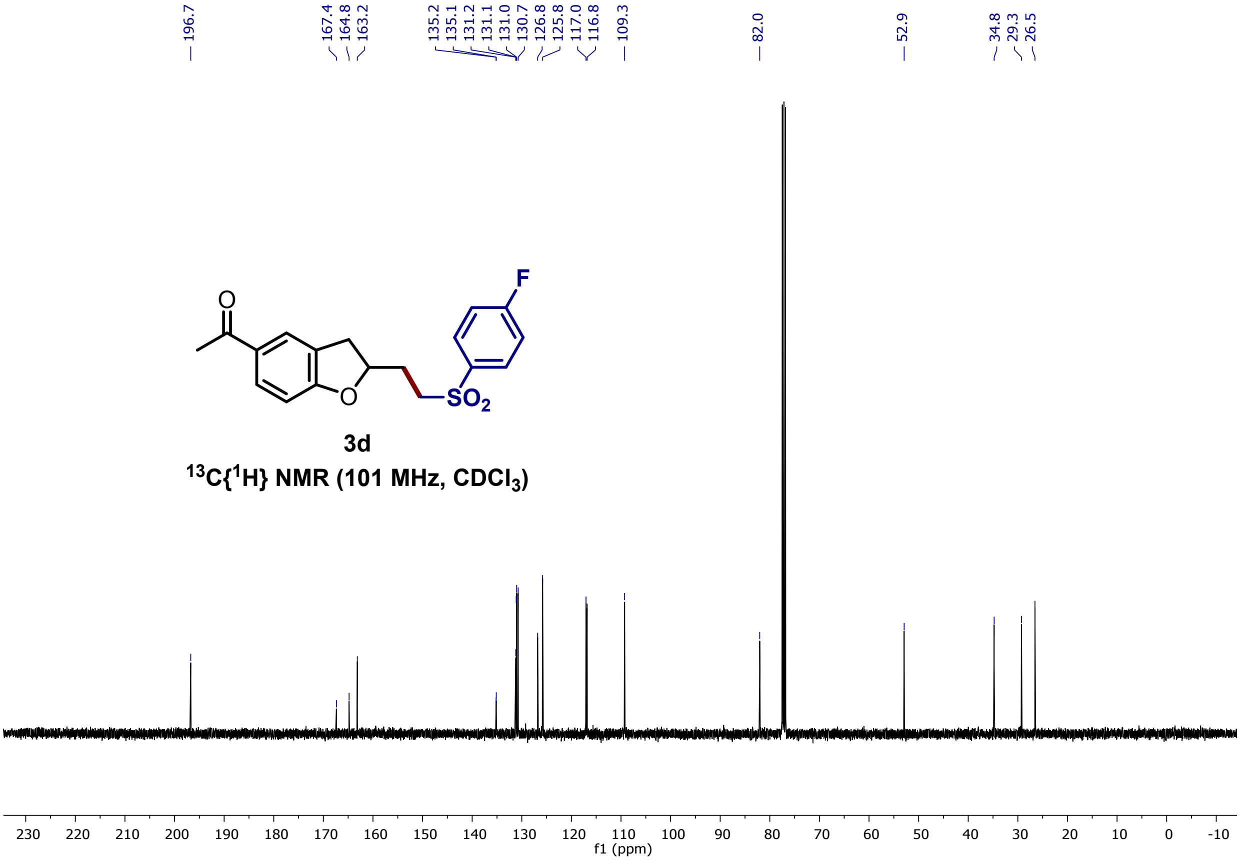

3e - ^{1}H NMR ($CDCl_3$).

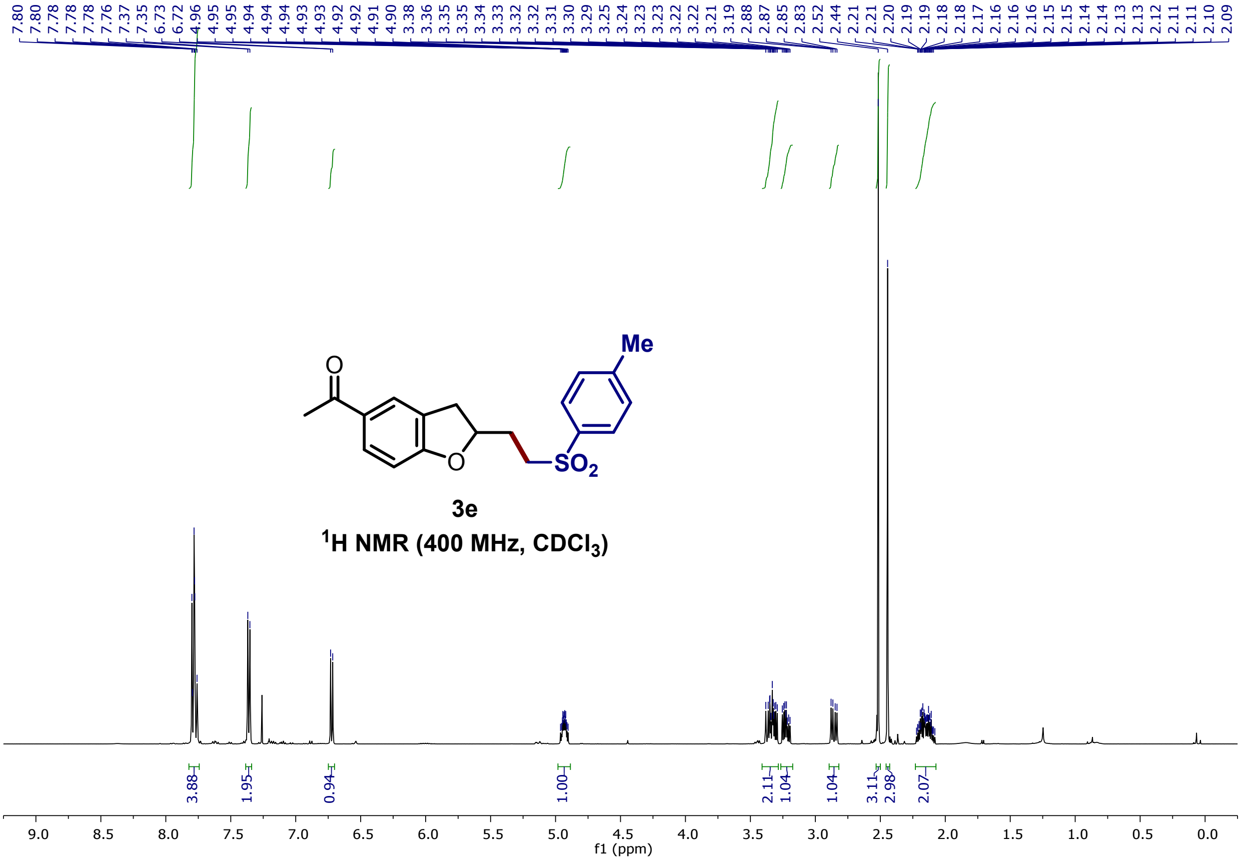

3e - ^{13}C{^{1}H} NMR ($CDCl_3$).

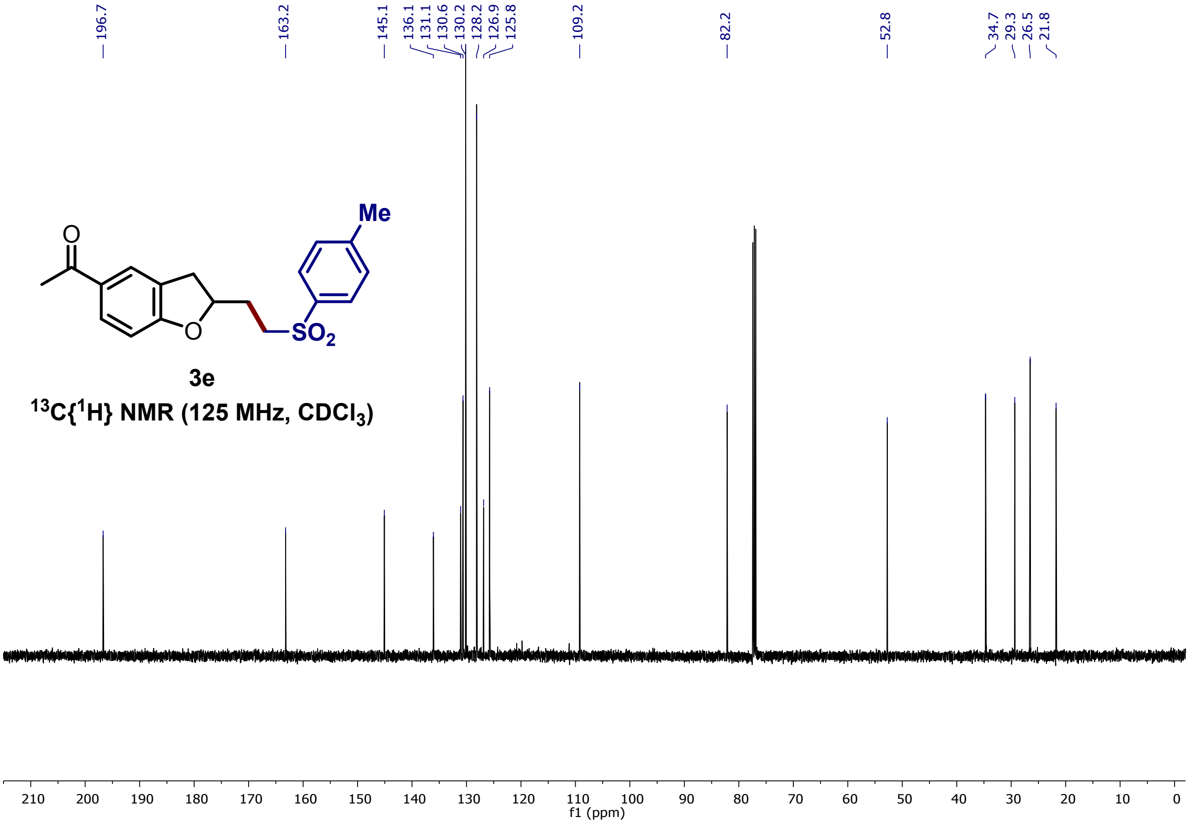

3f - ^{1}H NMR ($CDCl_3$).

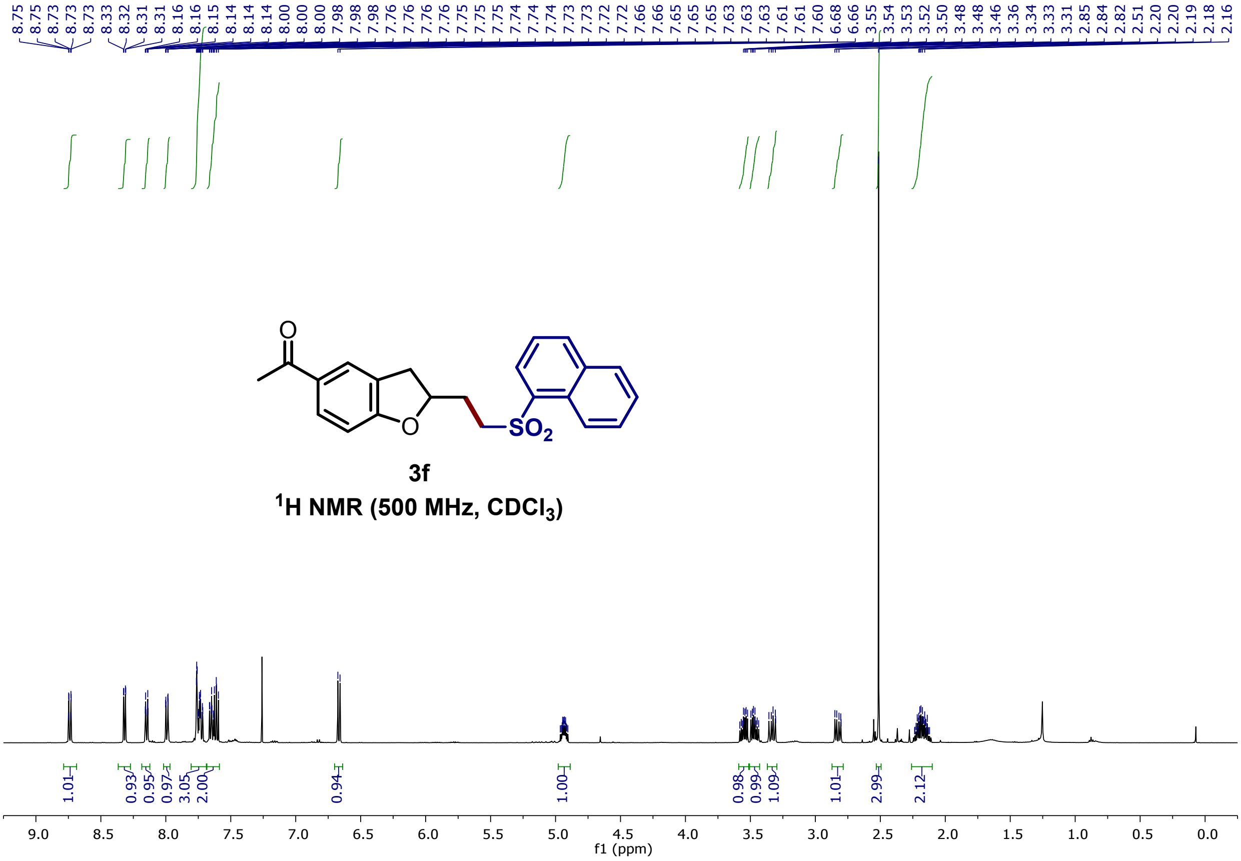

3e - $^{13}C\{^1H\}$ NMR ($CDCl_3$).

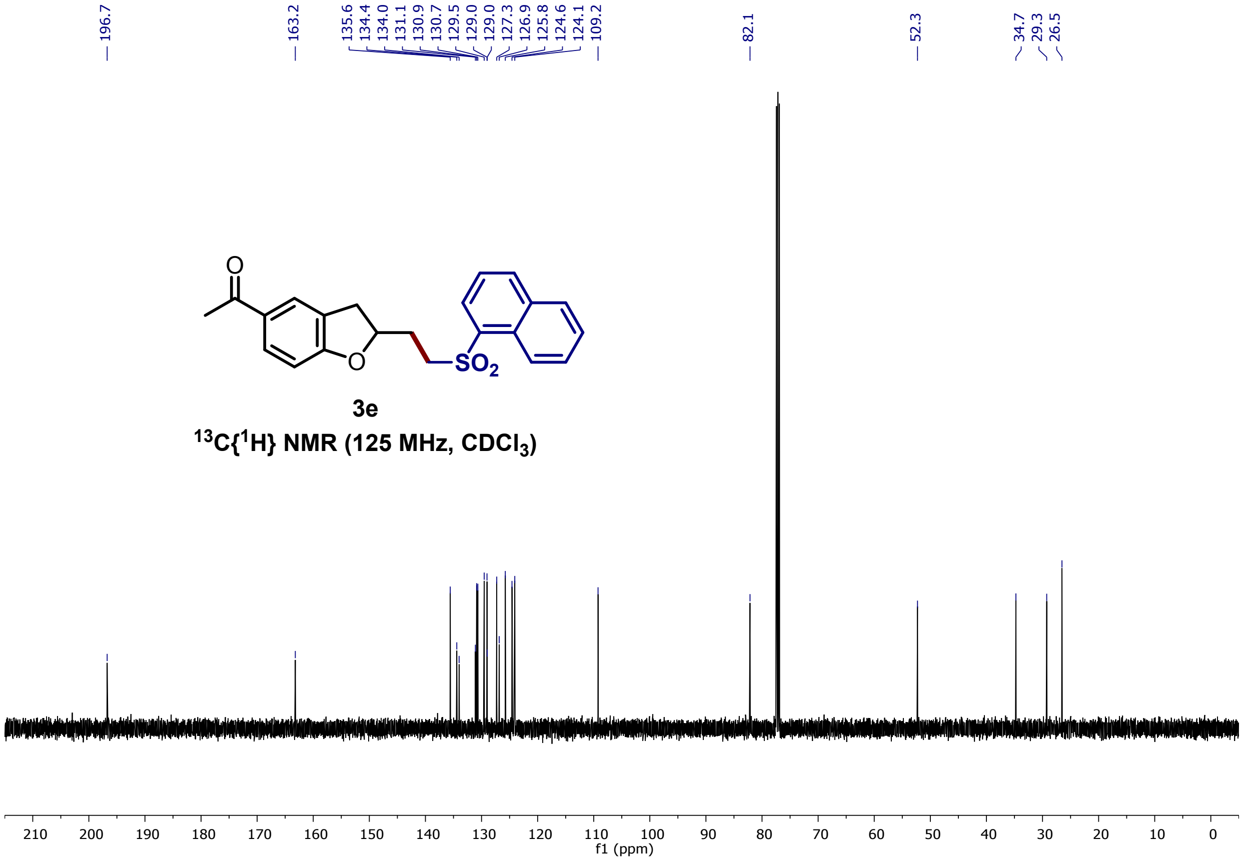

3g - ^{1}H NMR ($CDCl_3$).

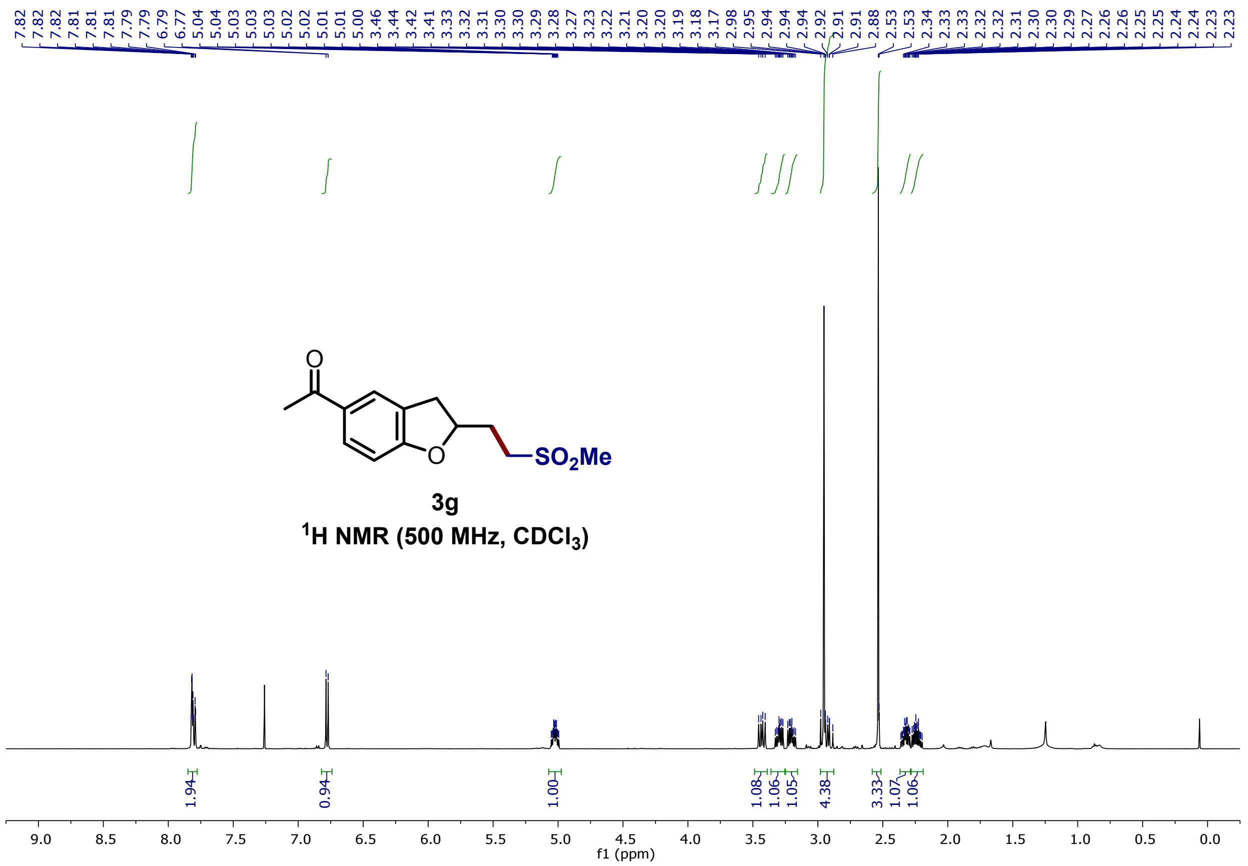

3g - $^{13}C\{^1H\}$ NMR ($CDCl_3$).

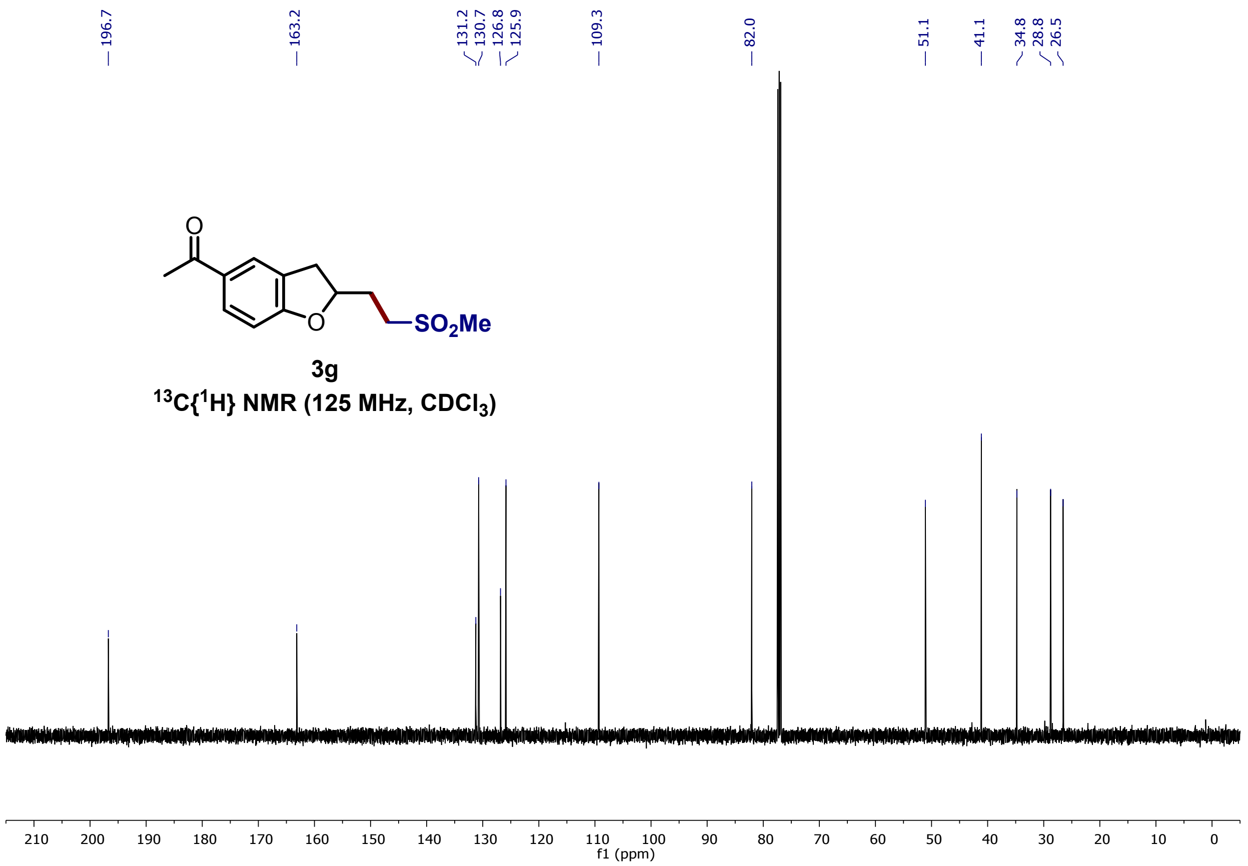

3h - ^{1}H NMR ($CDCl_3$).

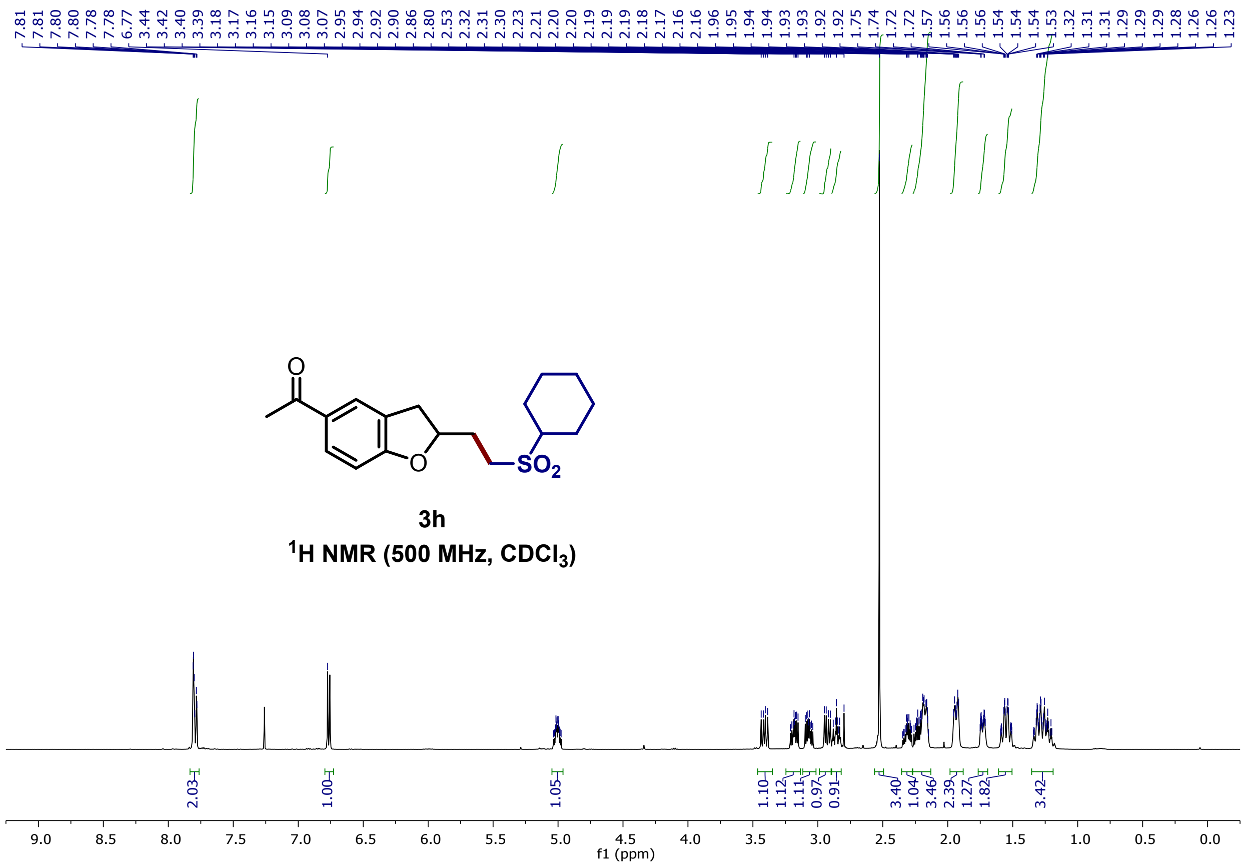

3g - $^{13}C\{^1H\}$ NMR ($CDCl_3$).

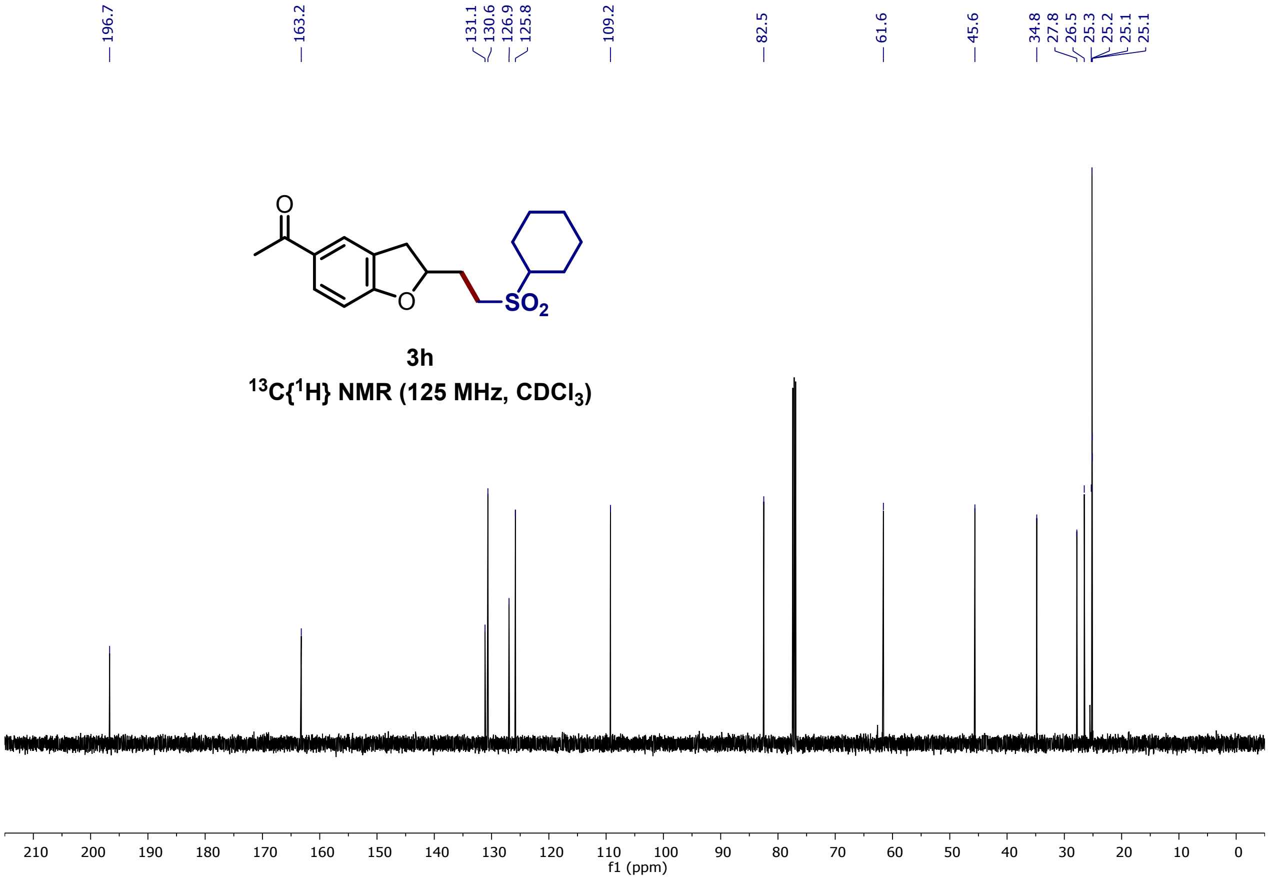

3i - ^{1}H NMR ($CDCl_3$).

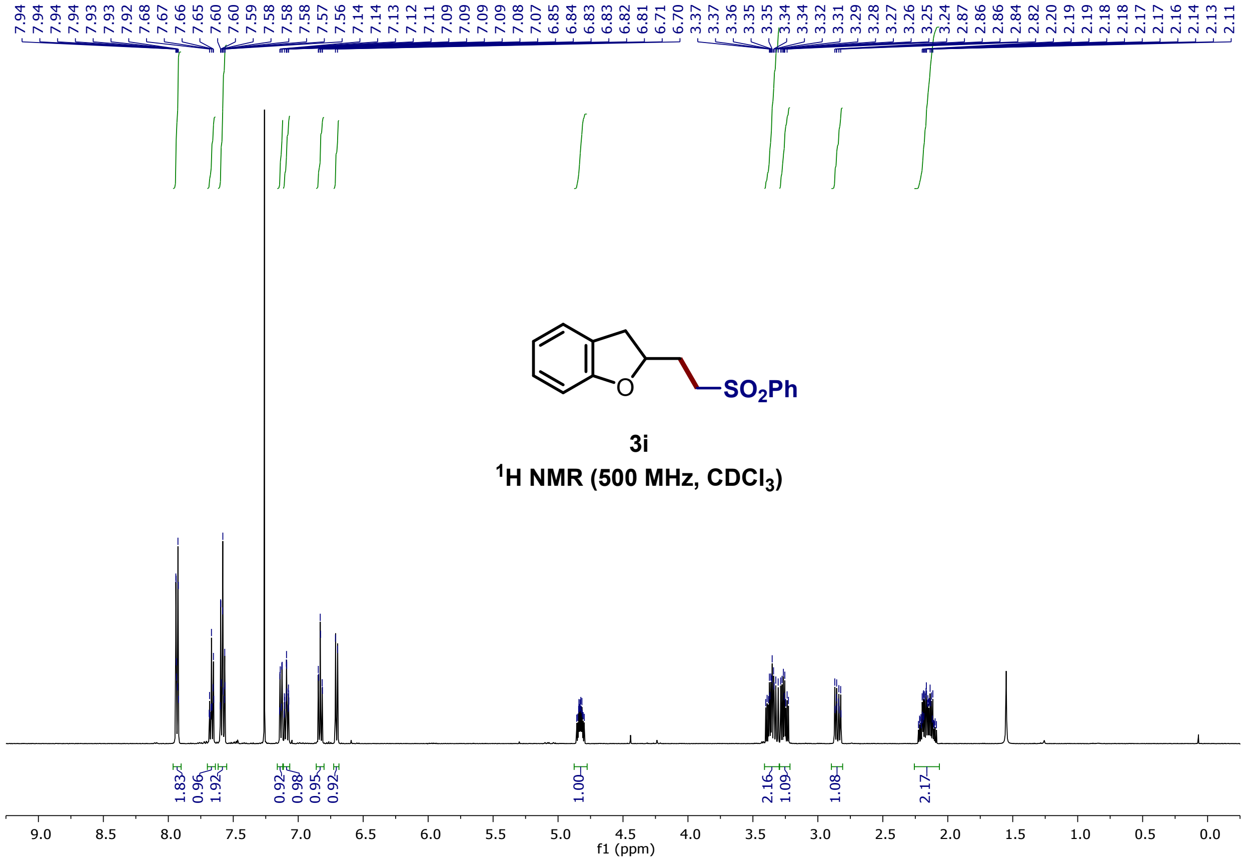

3i - ^{13}C{^{1}H} NMR ($CDCl_3$).

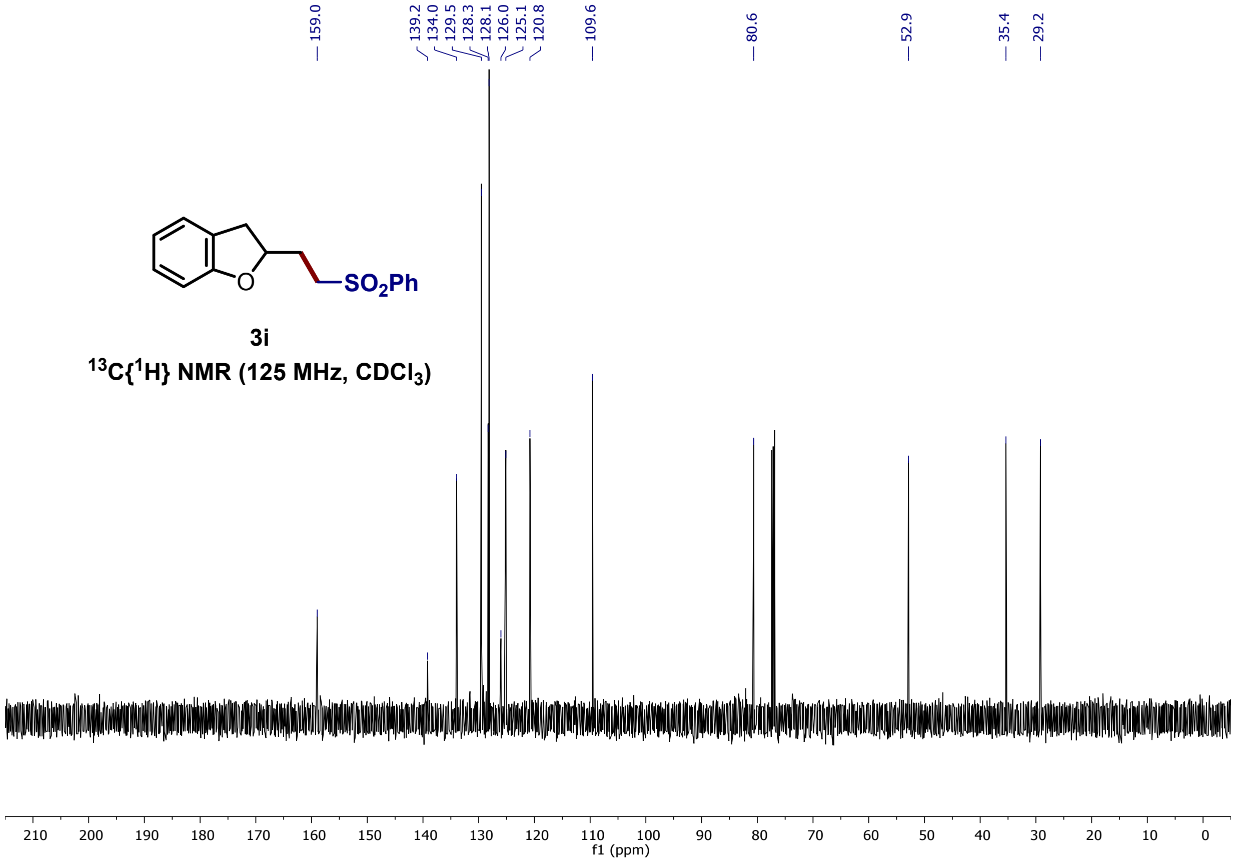

3j - ^{1}H NMR ($CDCl_3$).

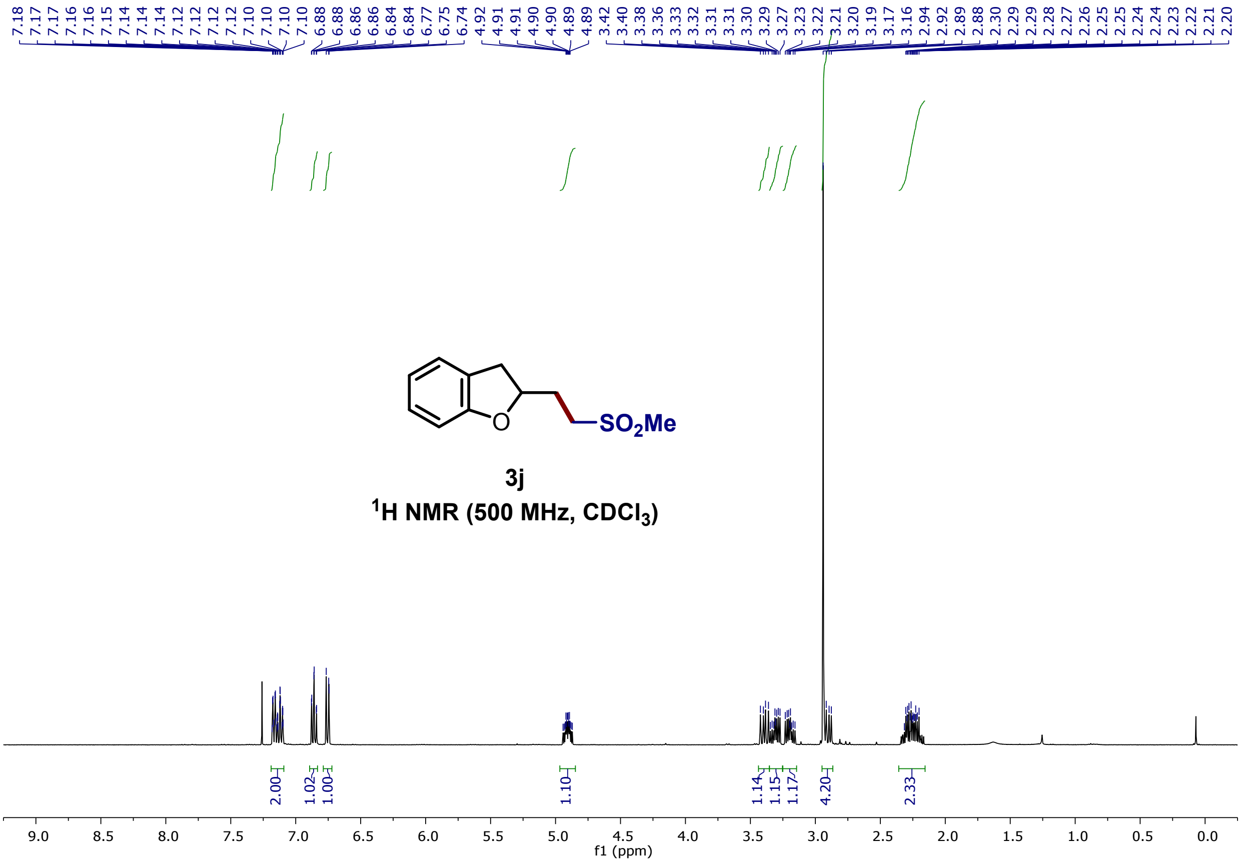

3j - $^{13}C\{^{1}H\}$ NMR ($CDCl_3$).

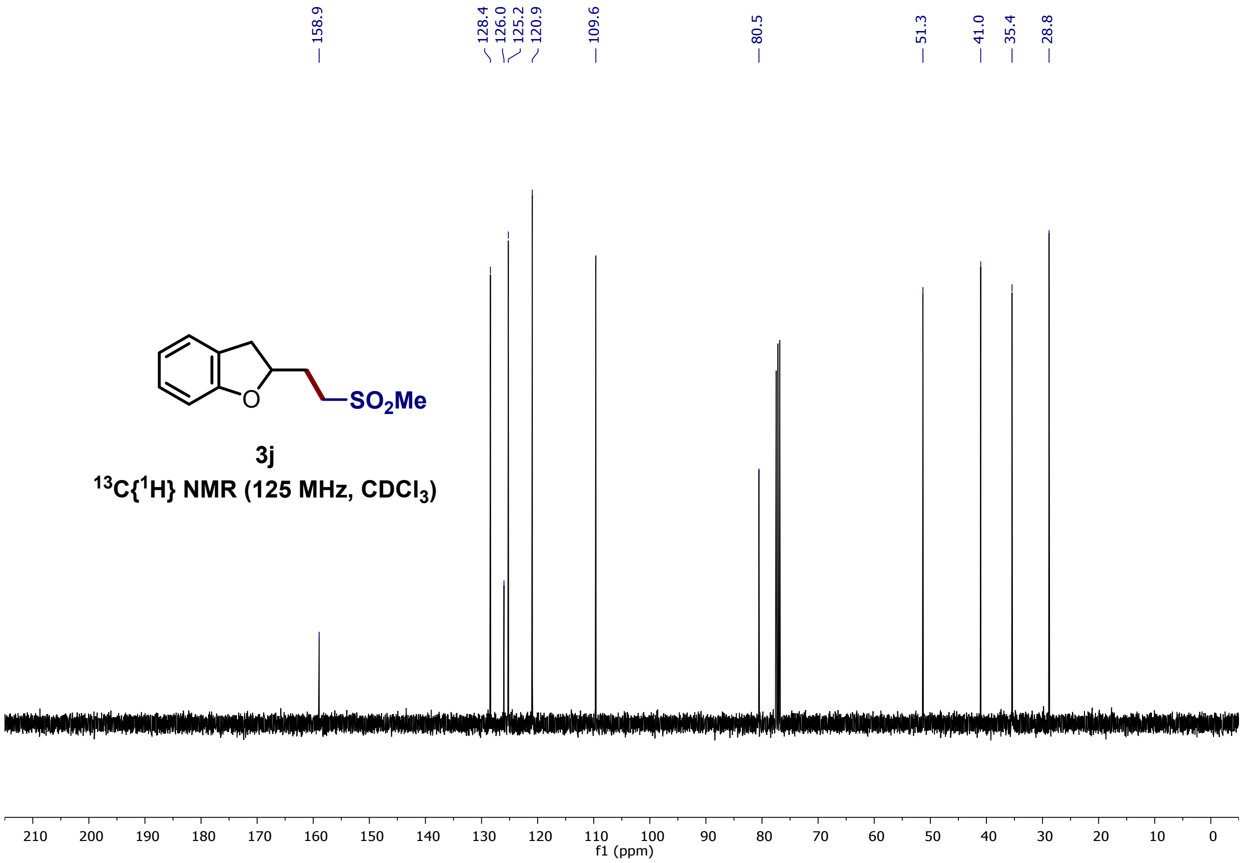

3k - ^{1}H NMR ($CDCl_3$).

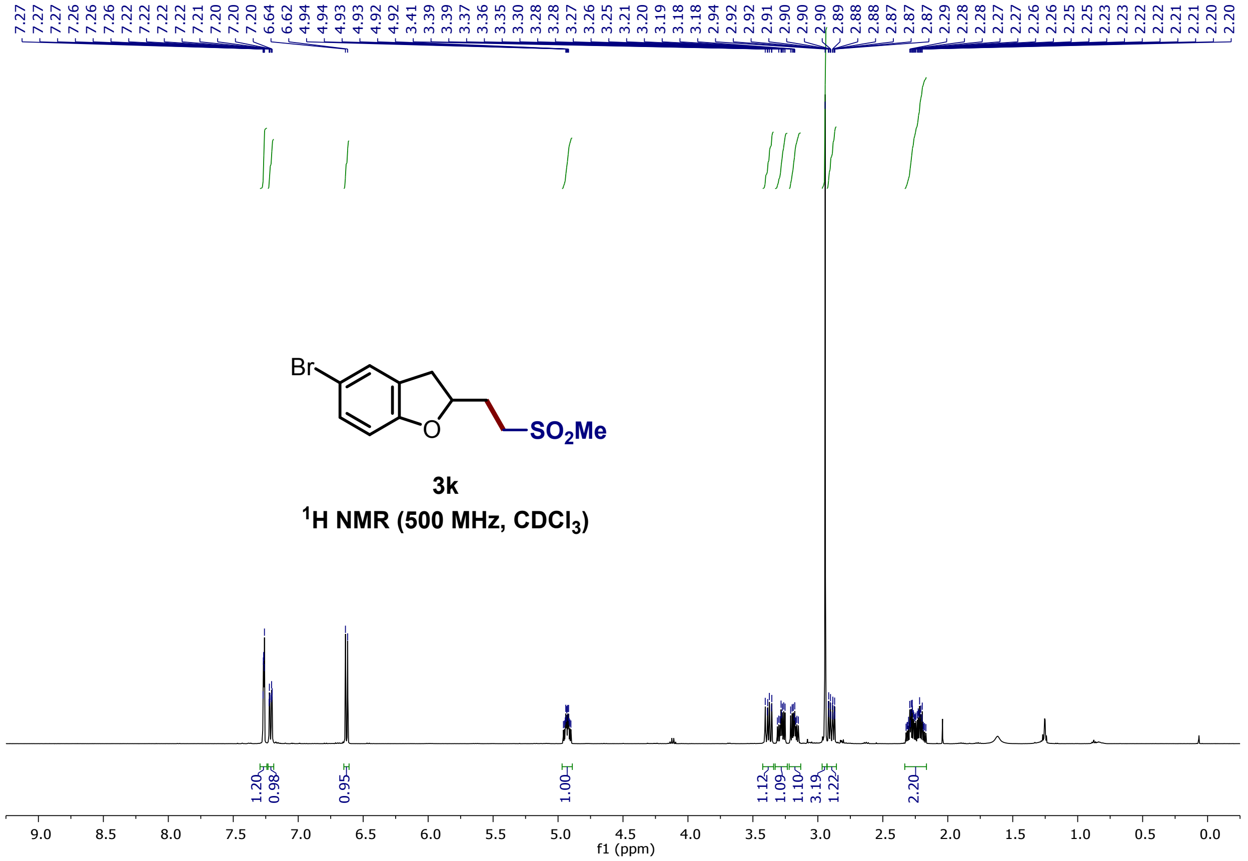

3k - $^{13}C\{^{1}H\}$ NMR ($CDCl_3$).

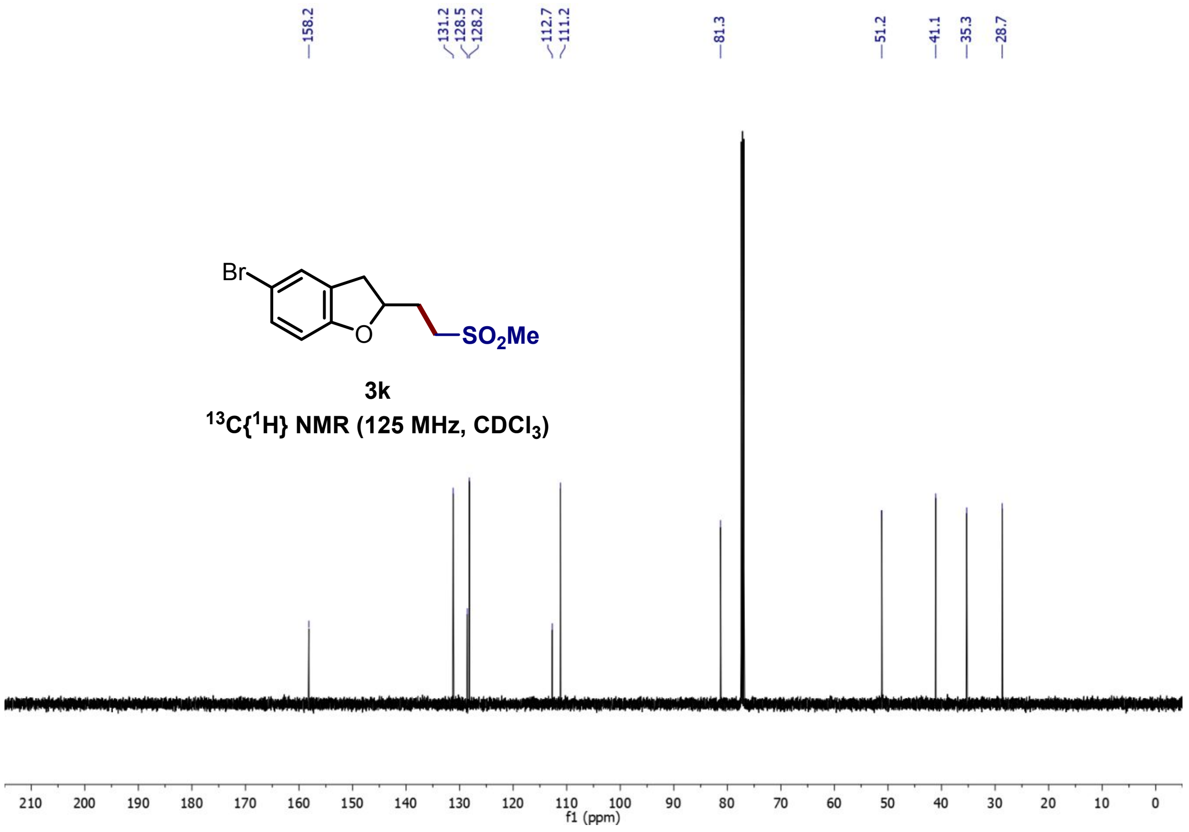

3l - ^{1}H NMR (CDCl$_3$).

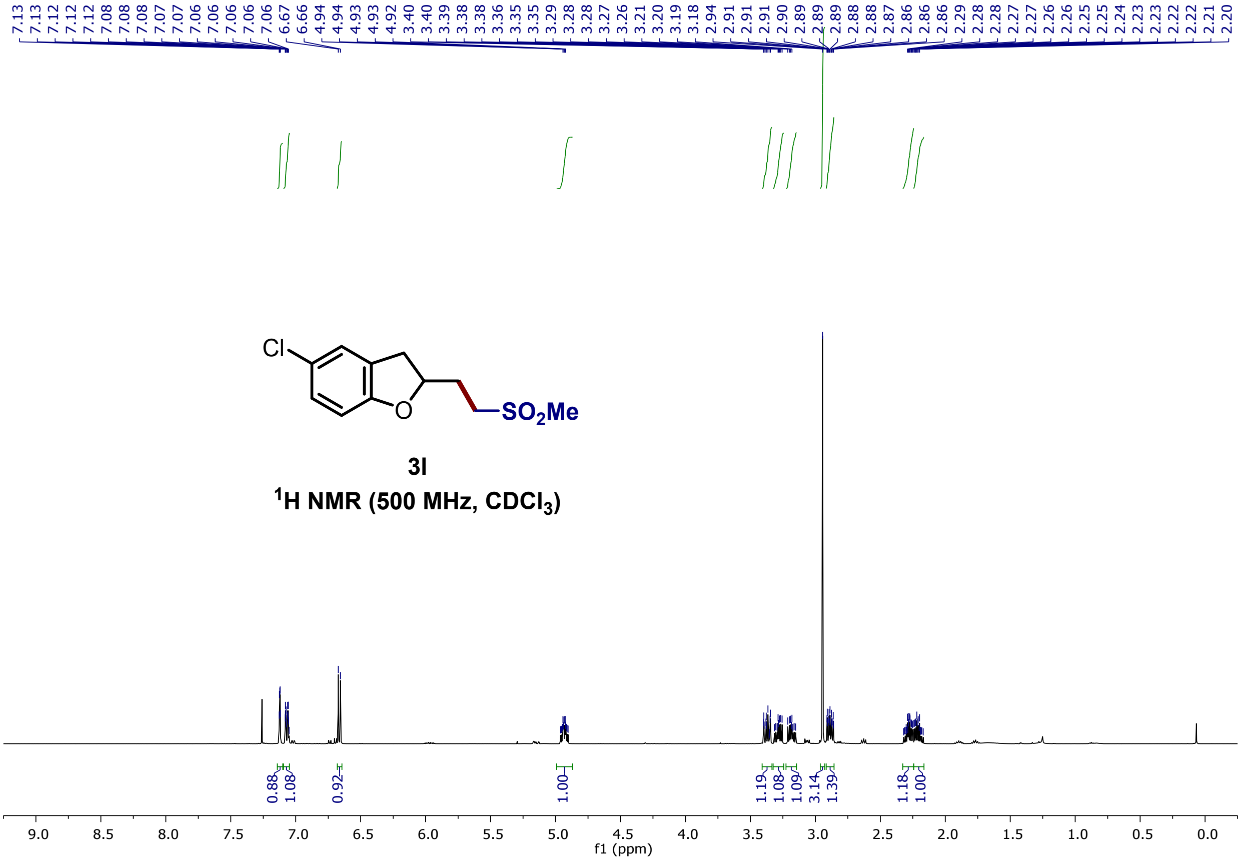

3l - ^{13}C{^{1}H} NMR (CDCl$_3$).

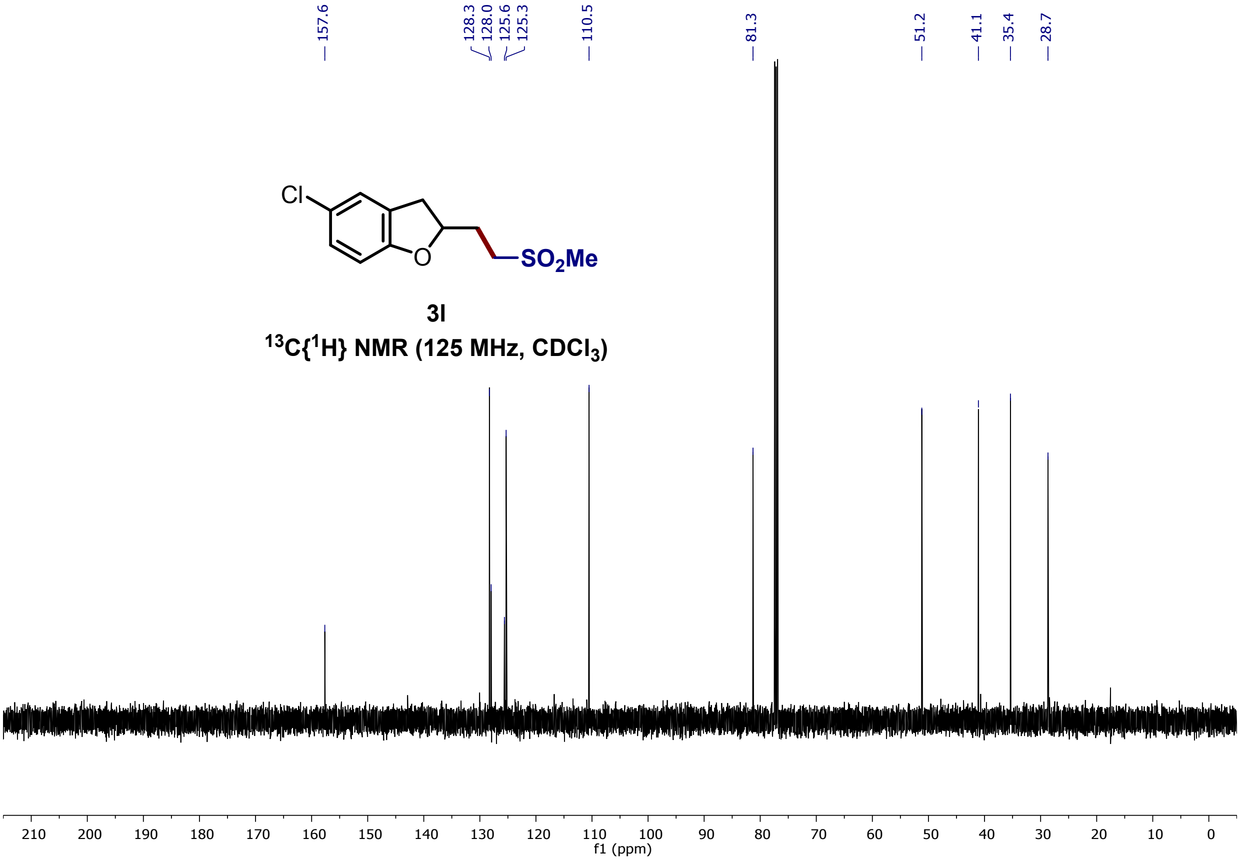

3m - ^{1}H NMR ($CDCl_3$).

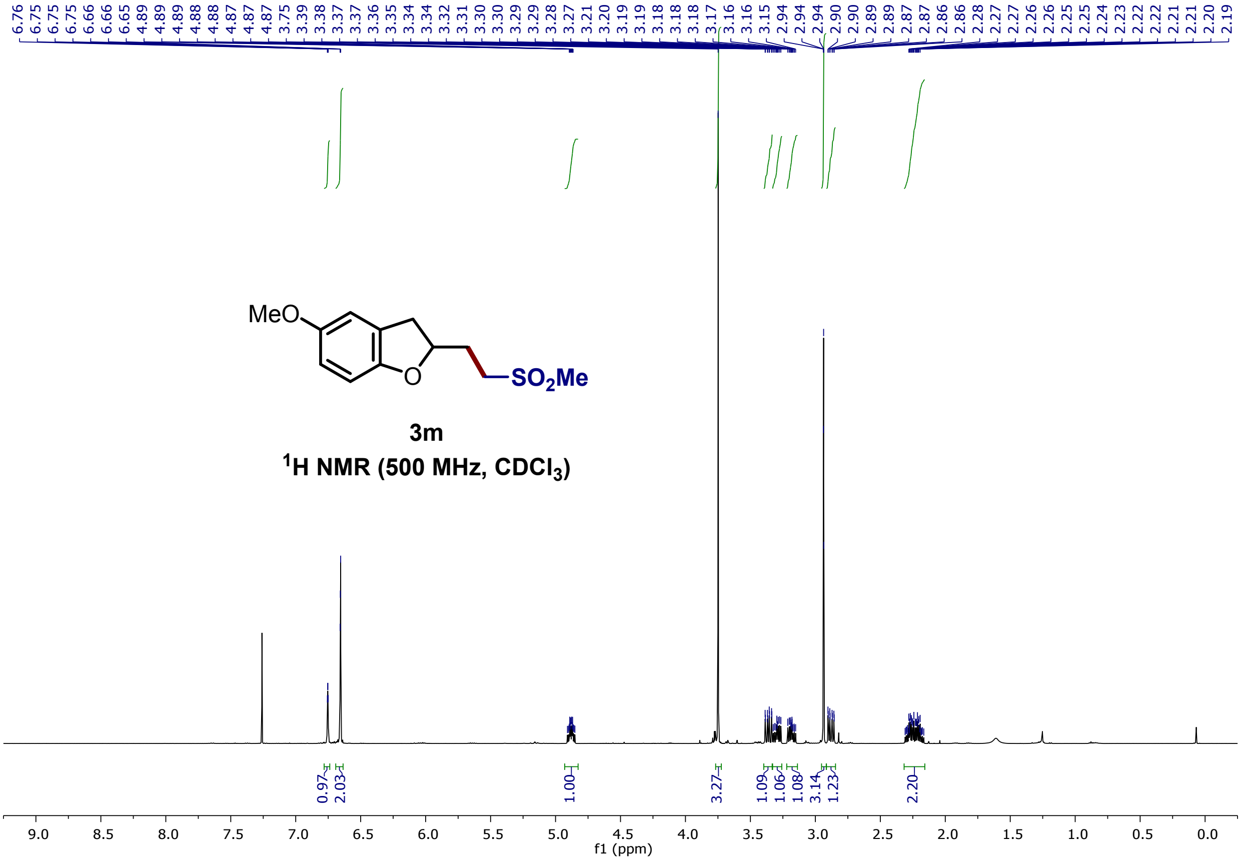

3m - $^{13}C\{^1H\}$ NMR ($CDCl_3$).

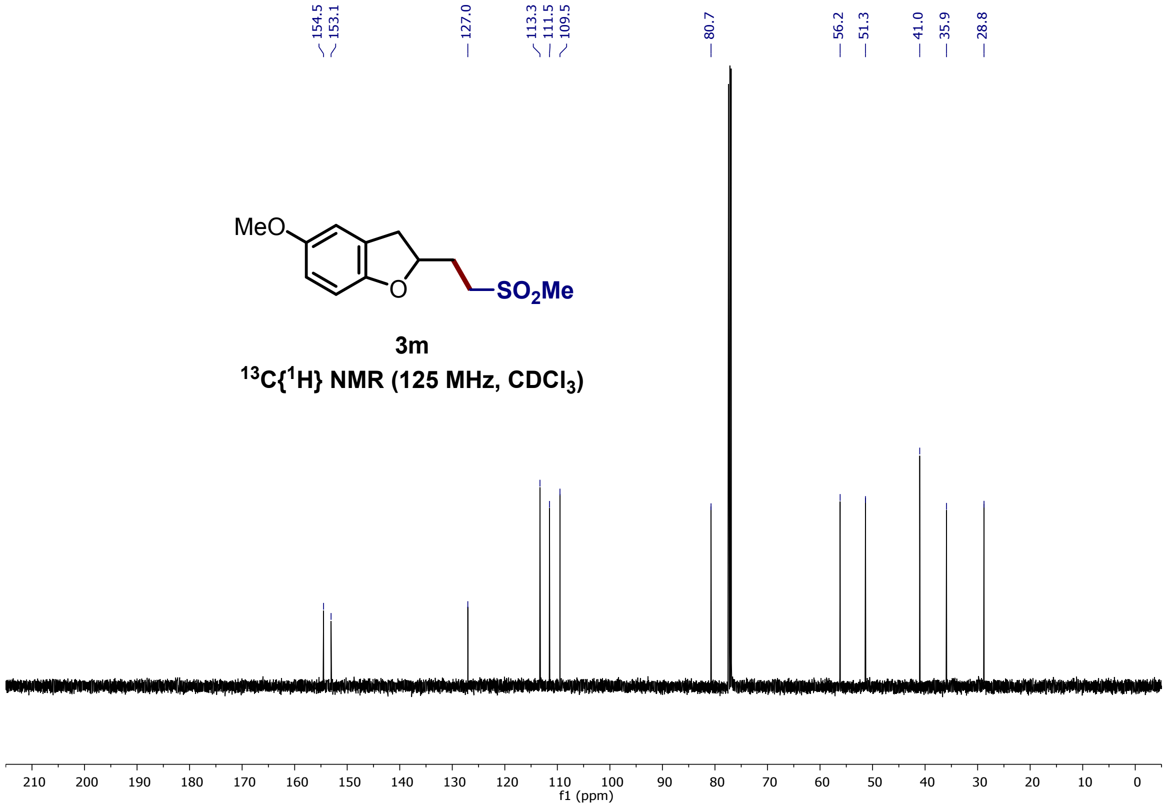

3n - ^1H NMR ($CDCl_3$).

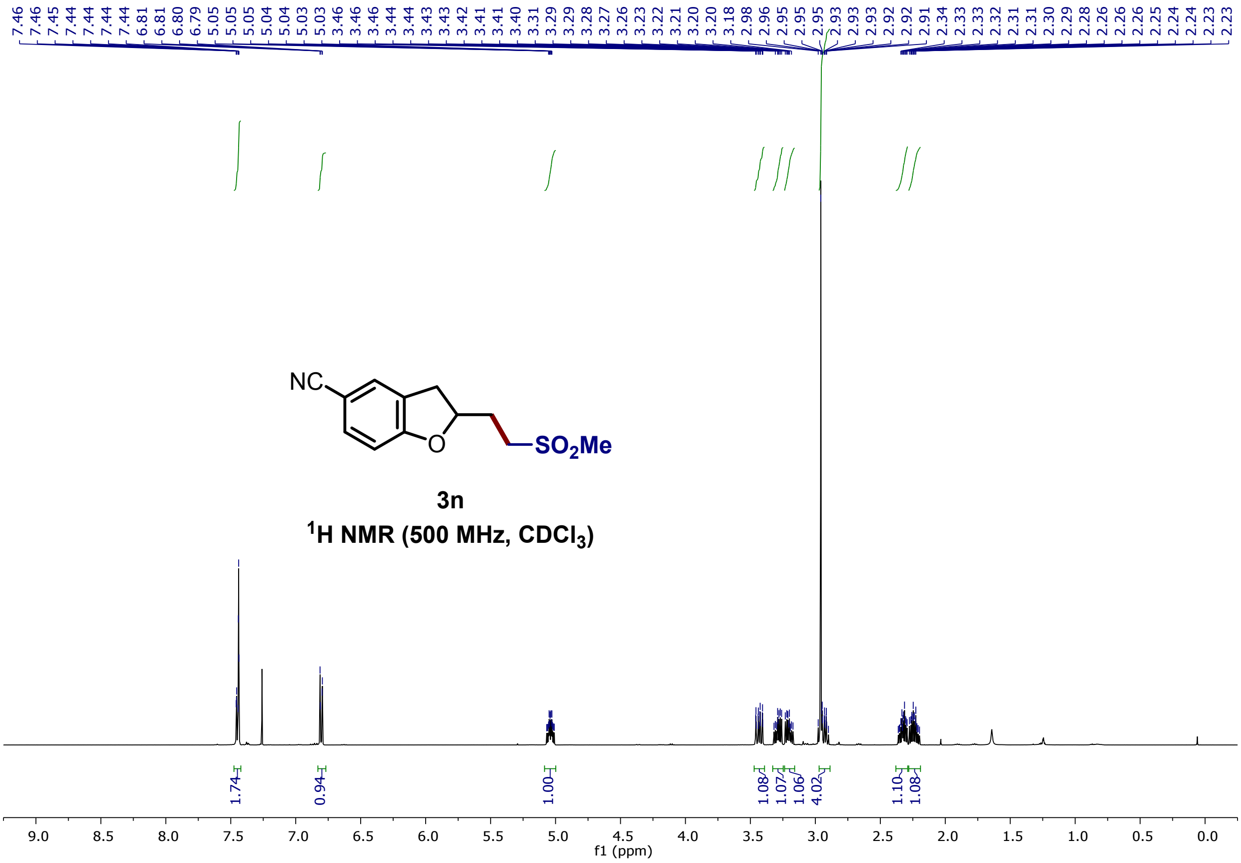

3n - $^{13}C\{^1H\}$ NMR ($CDCl_3$).

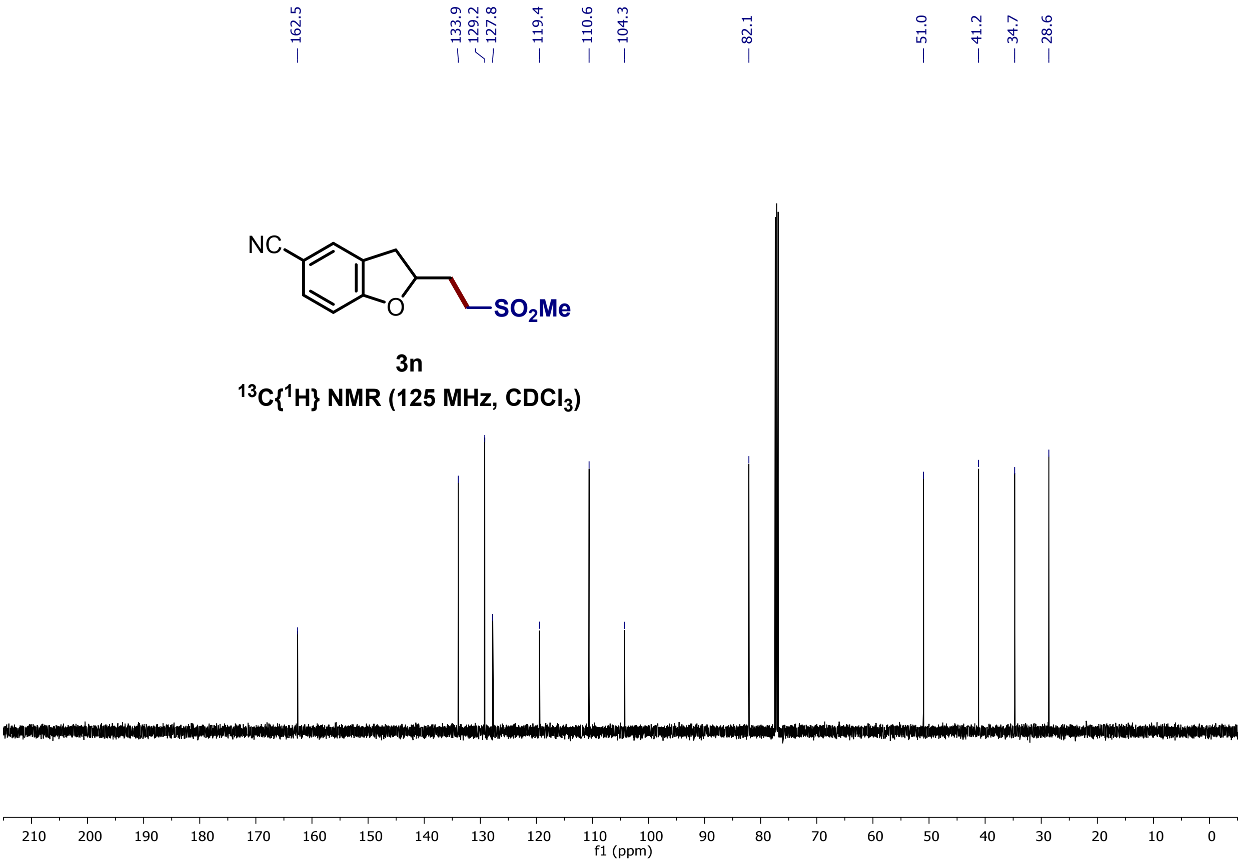

3o - ^{1}H NMR ($CDCl_3$).

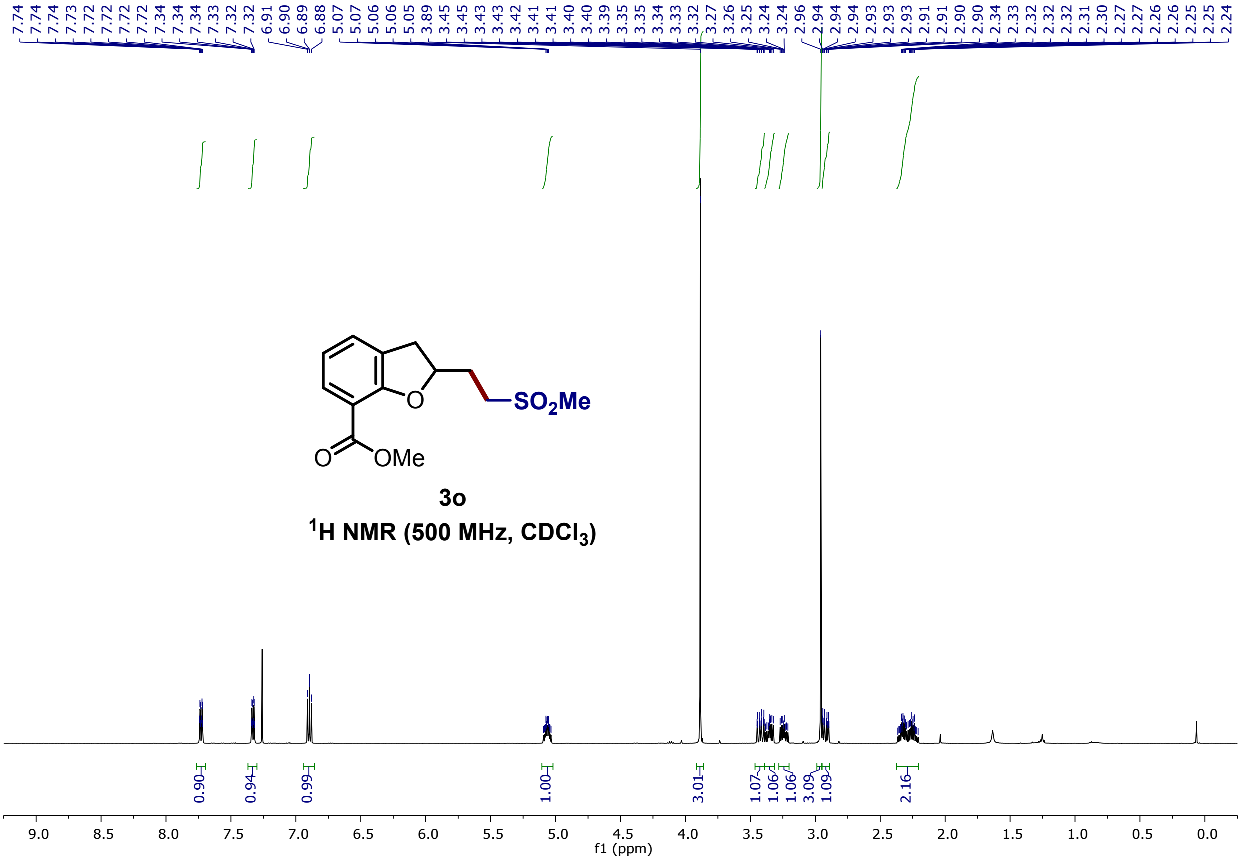

3o - ^{13}C{^{1}H} NMR ($CDCl_3$).

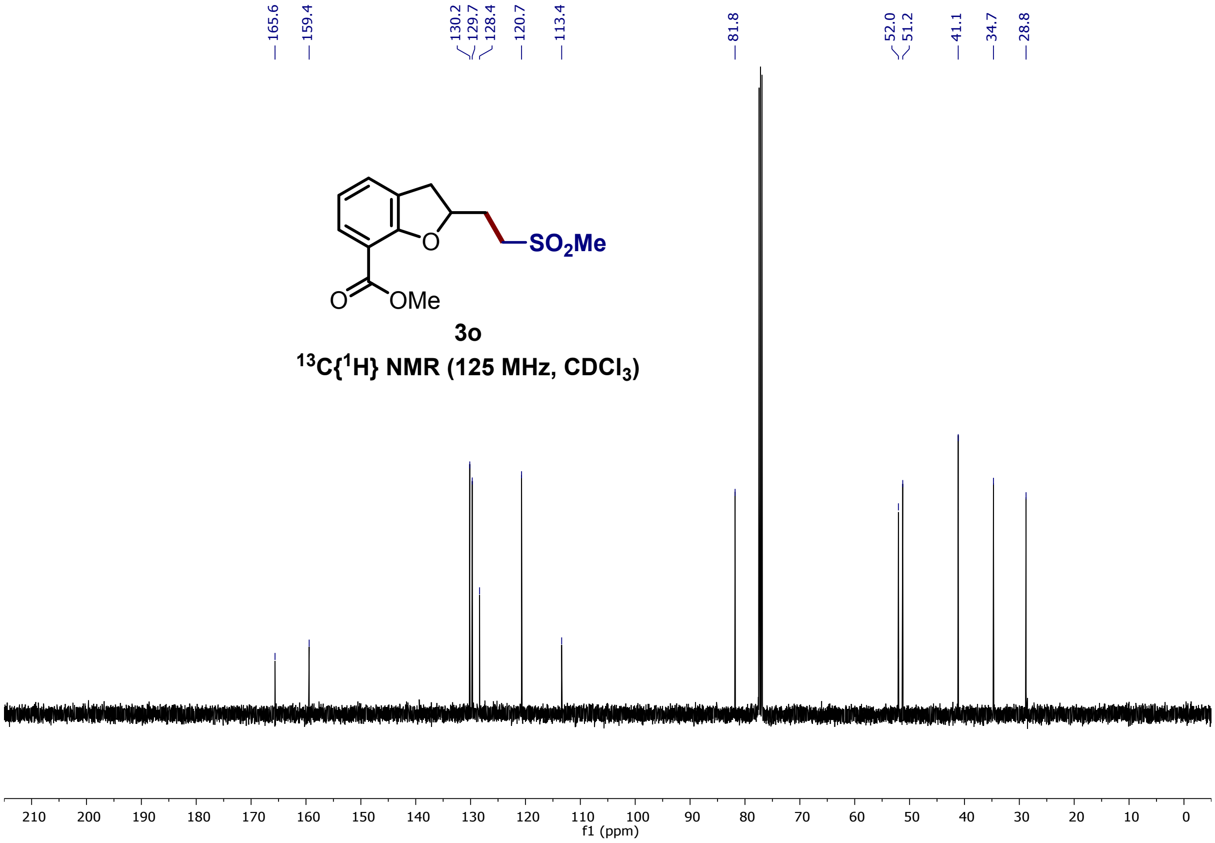

3p - ^{1}H NMR ($CDCl_3$).

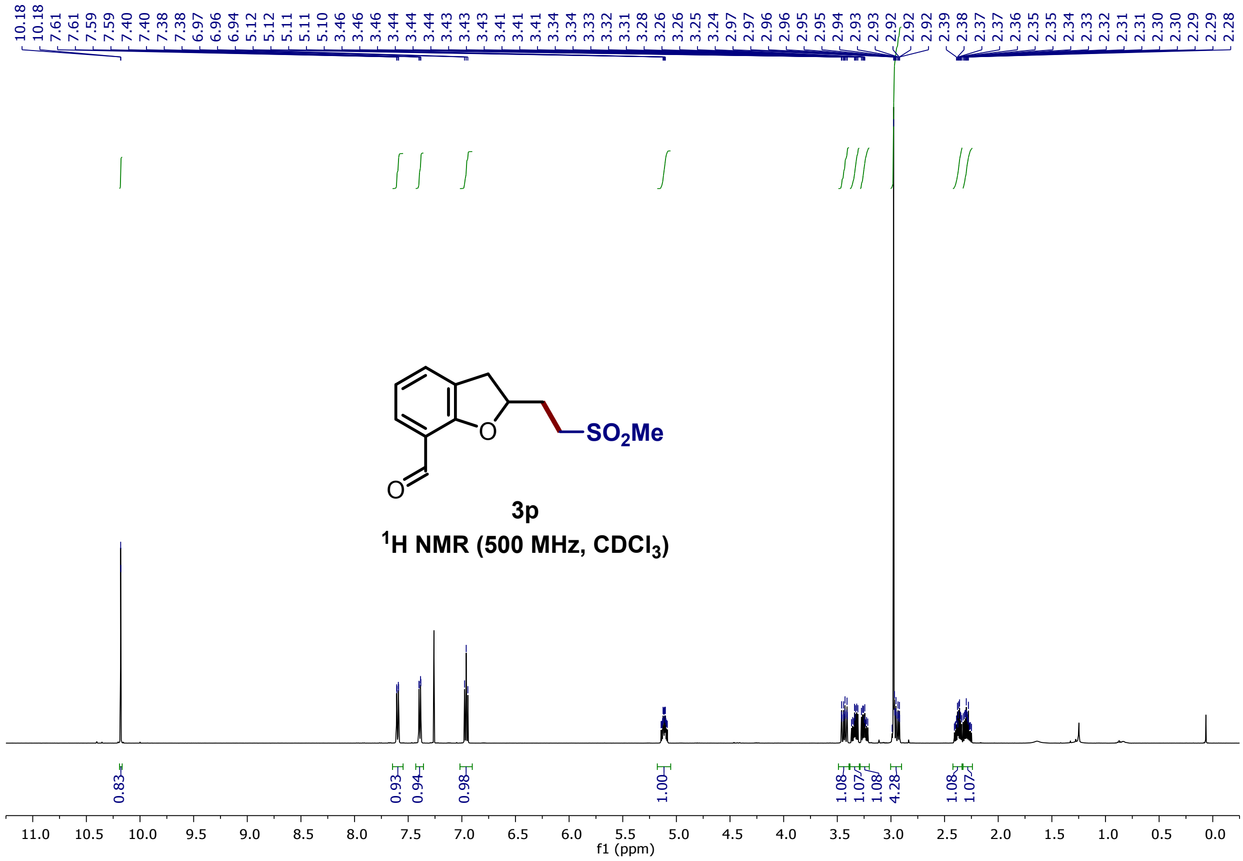

3p - $^{13}C\{^{1}H\}$ NMR ($CDCl_3$).

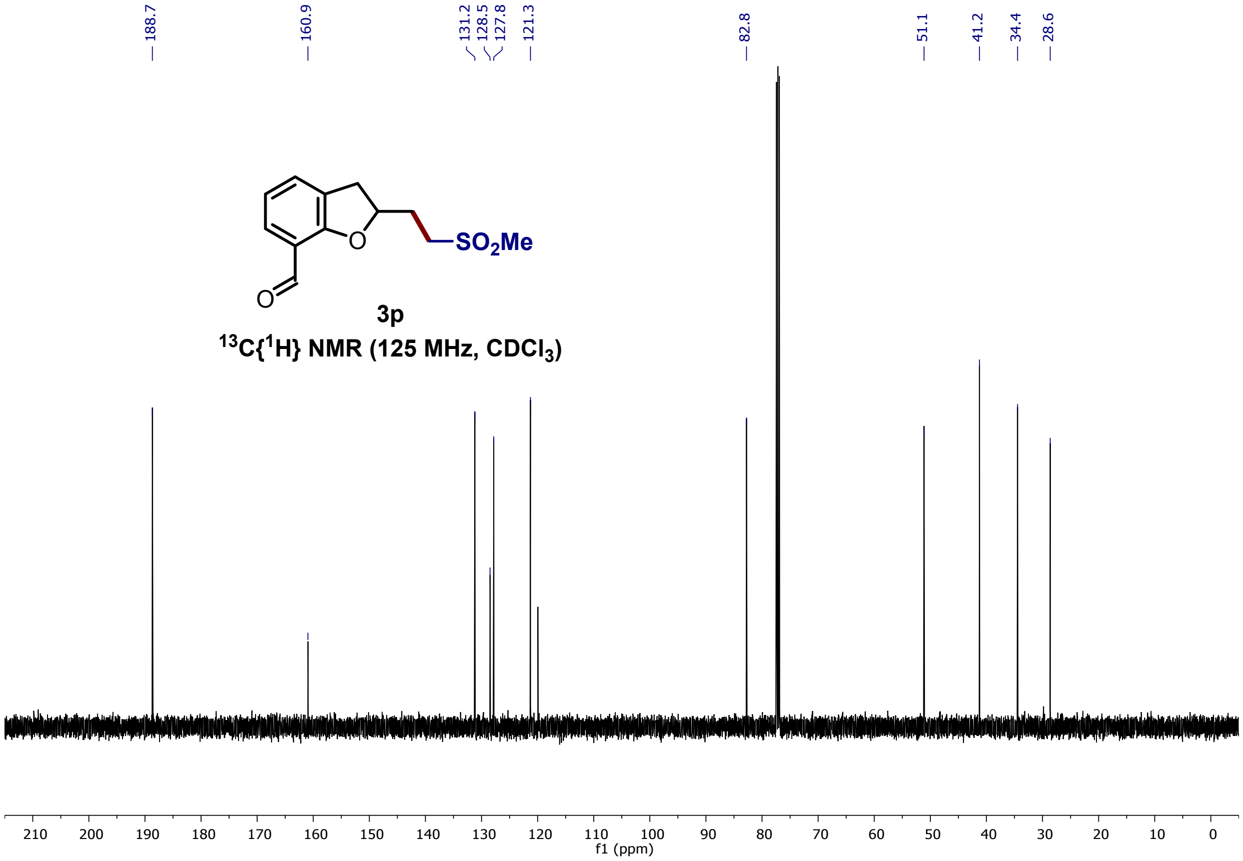

3q - ^{1}H NMR ($CDCl_3$).

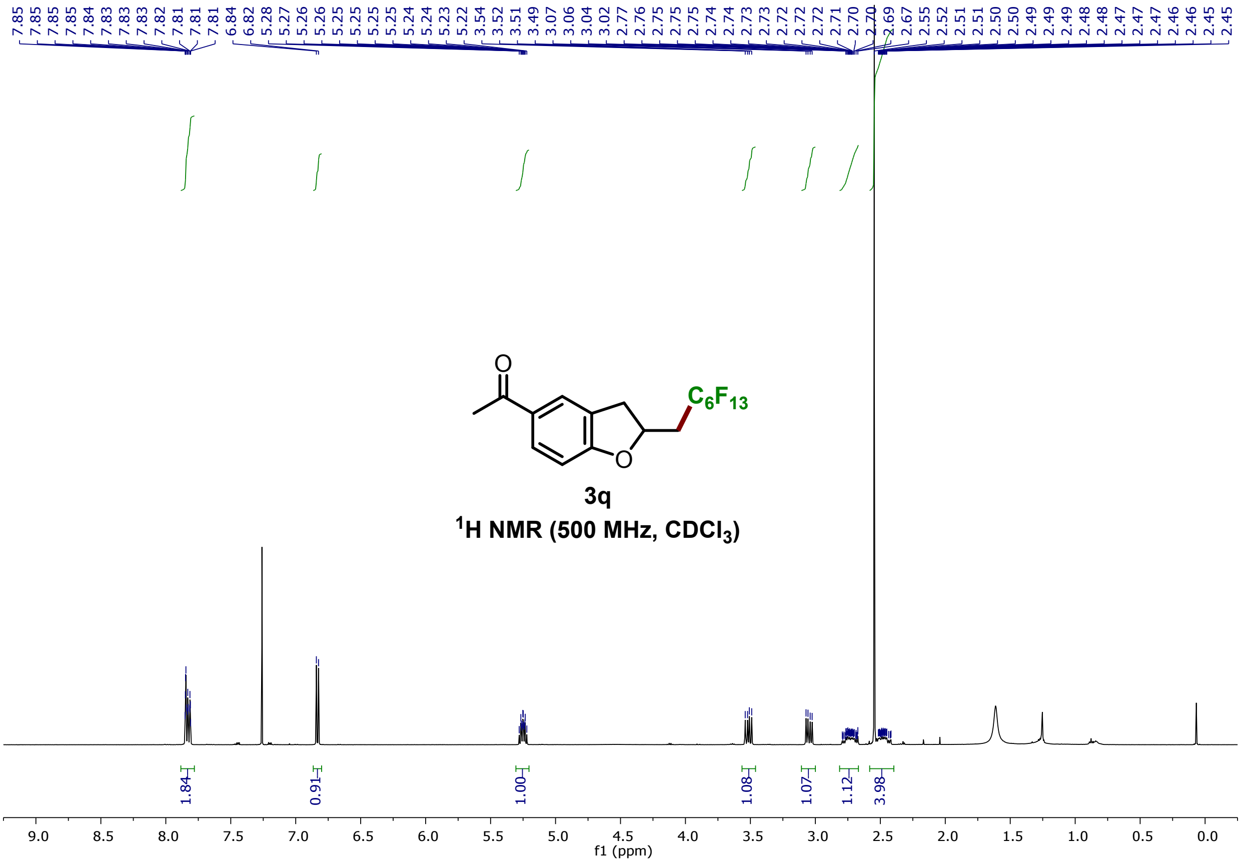

3q – ^{19}F NMR ($CDCl_3$).

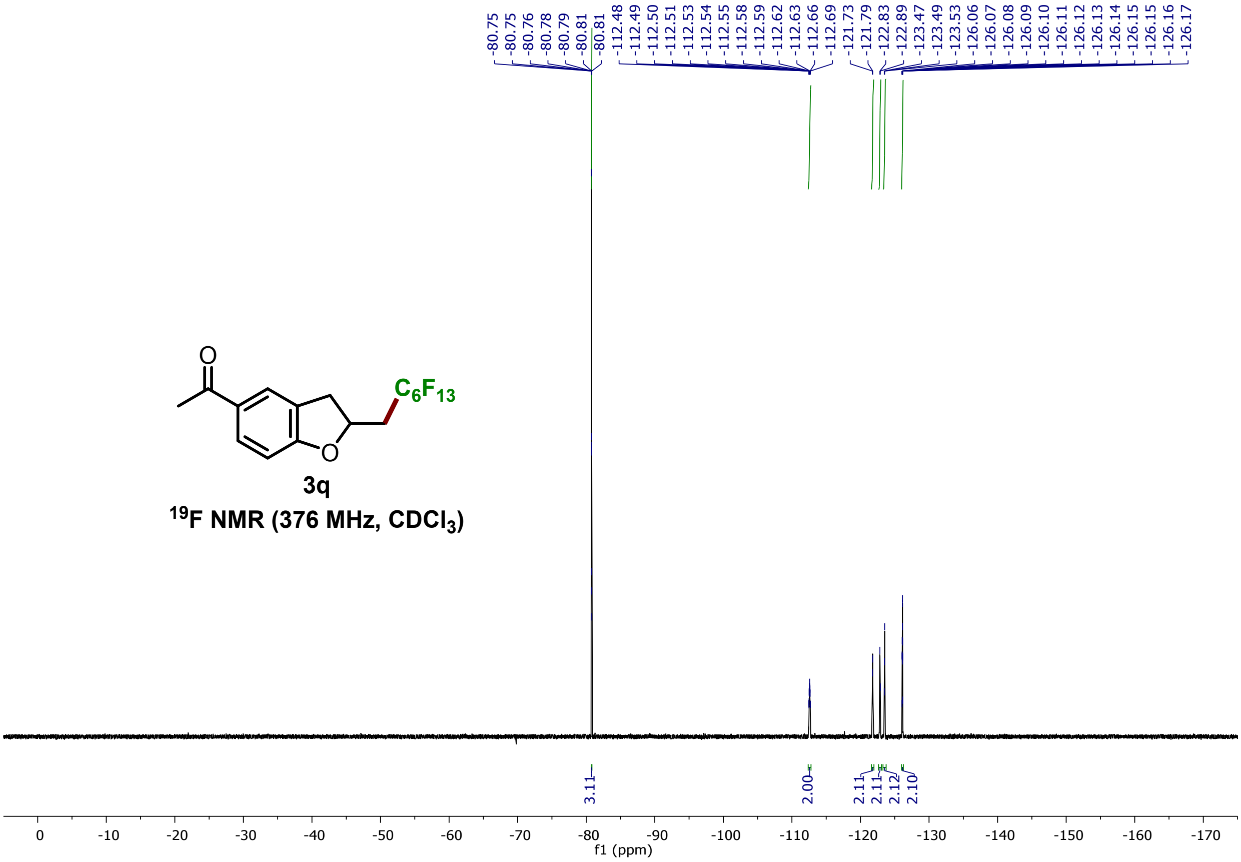

3q - $^{13}C\{^1H\}$ NMR ($CDCl_3$).

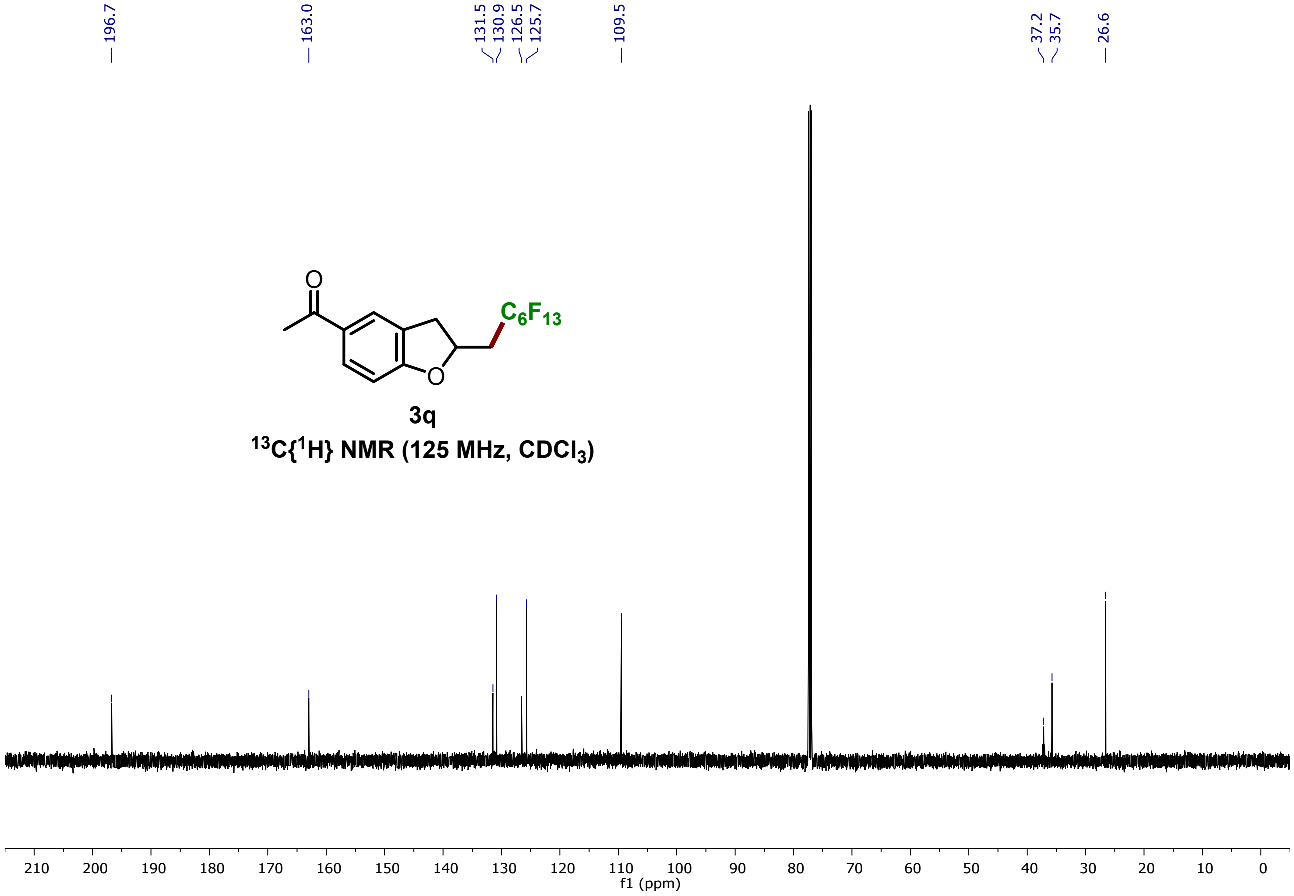

3r - ^{1}H NMR ($CDCl_3$).

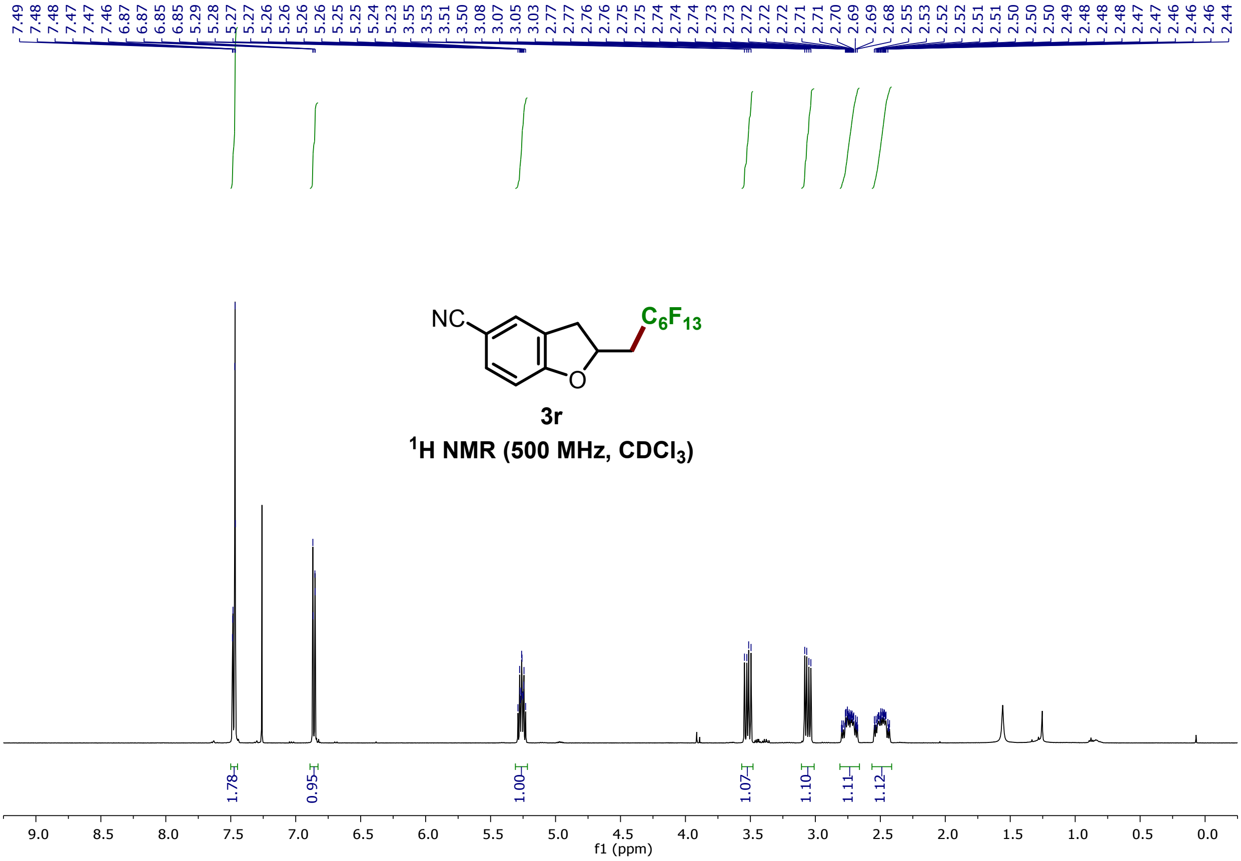

3q – ^{19}F NMR ($CDCl_3$).

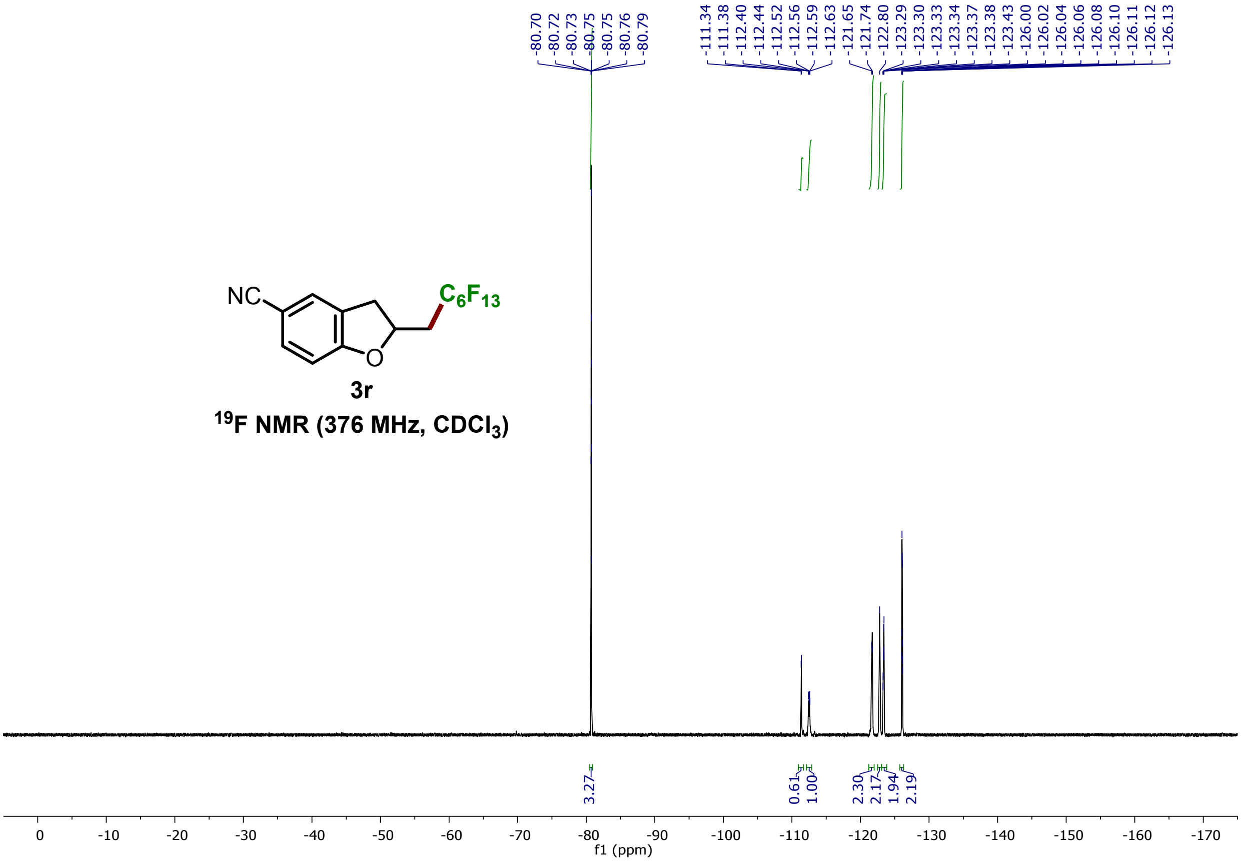

3r - $^{13}C\{^1H\}$ NMR ($CDCl_3$).

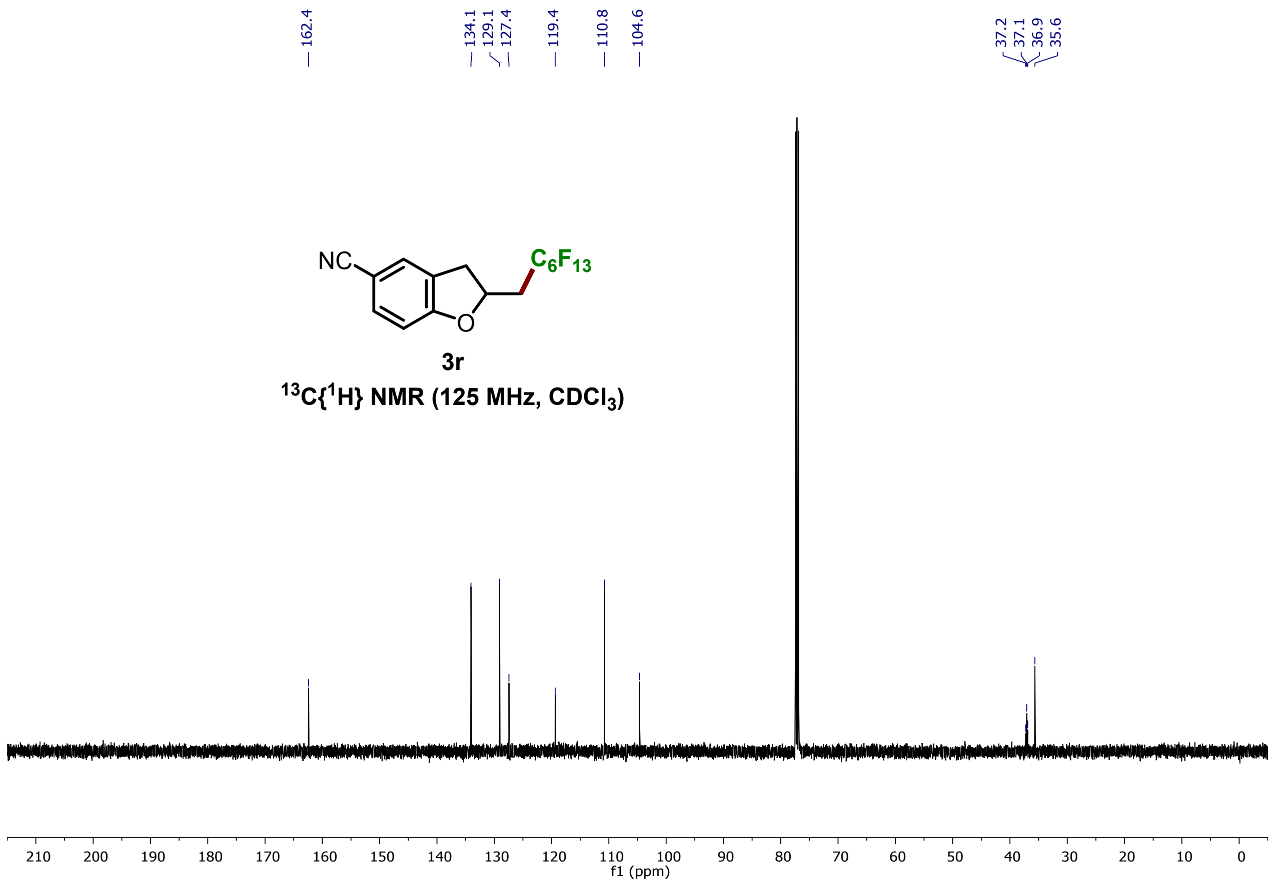

3s - ^{1}H NMR ($CDCl_3$).

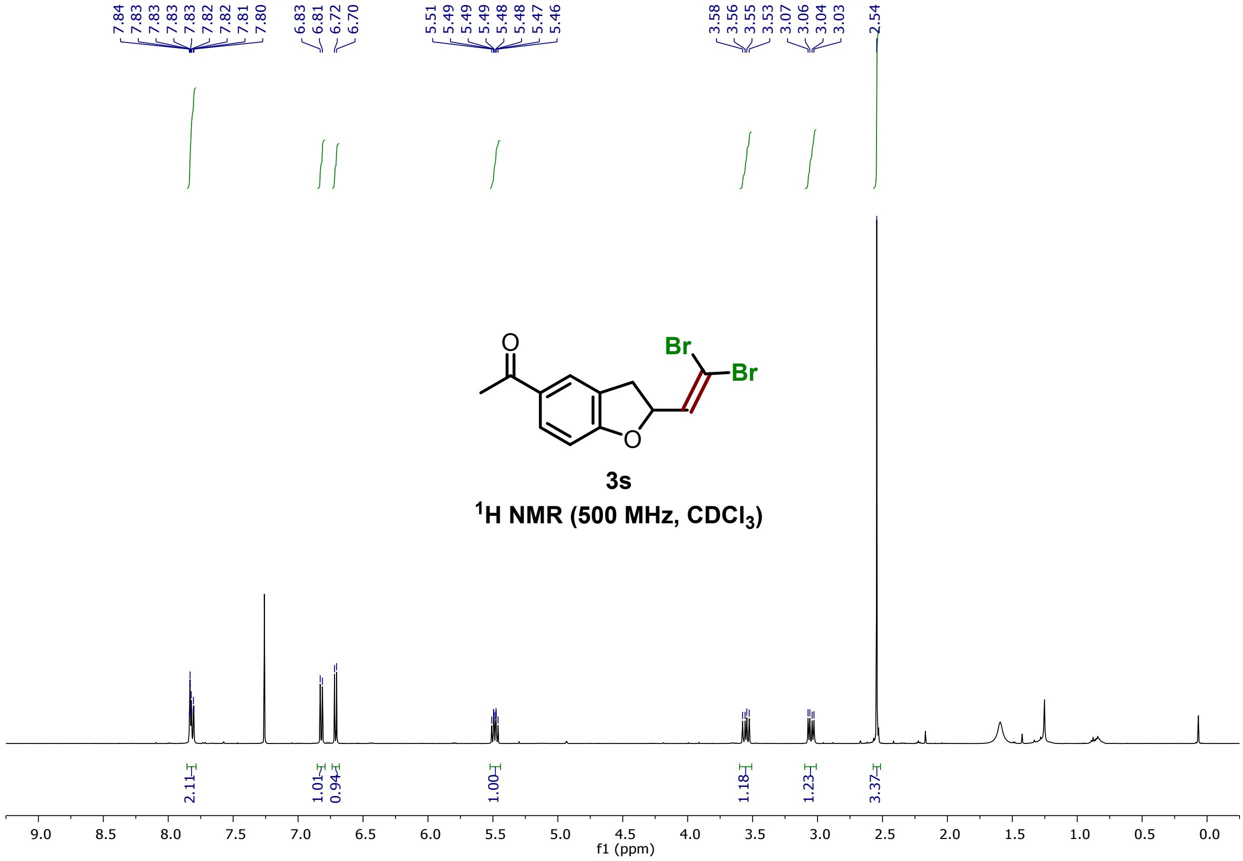

3s - $^{13}C\{^1H\}$ NMR ($CDCl_3$).

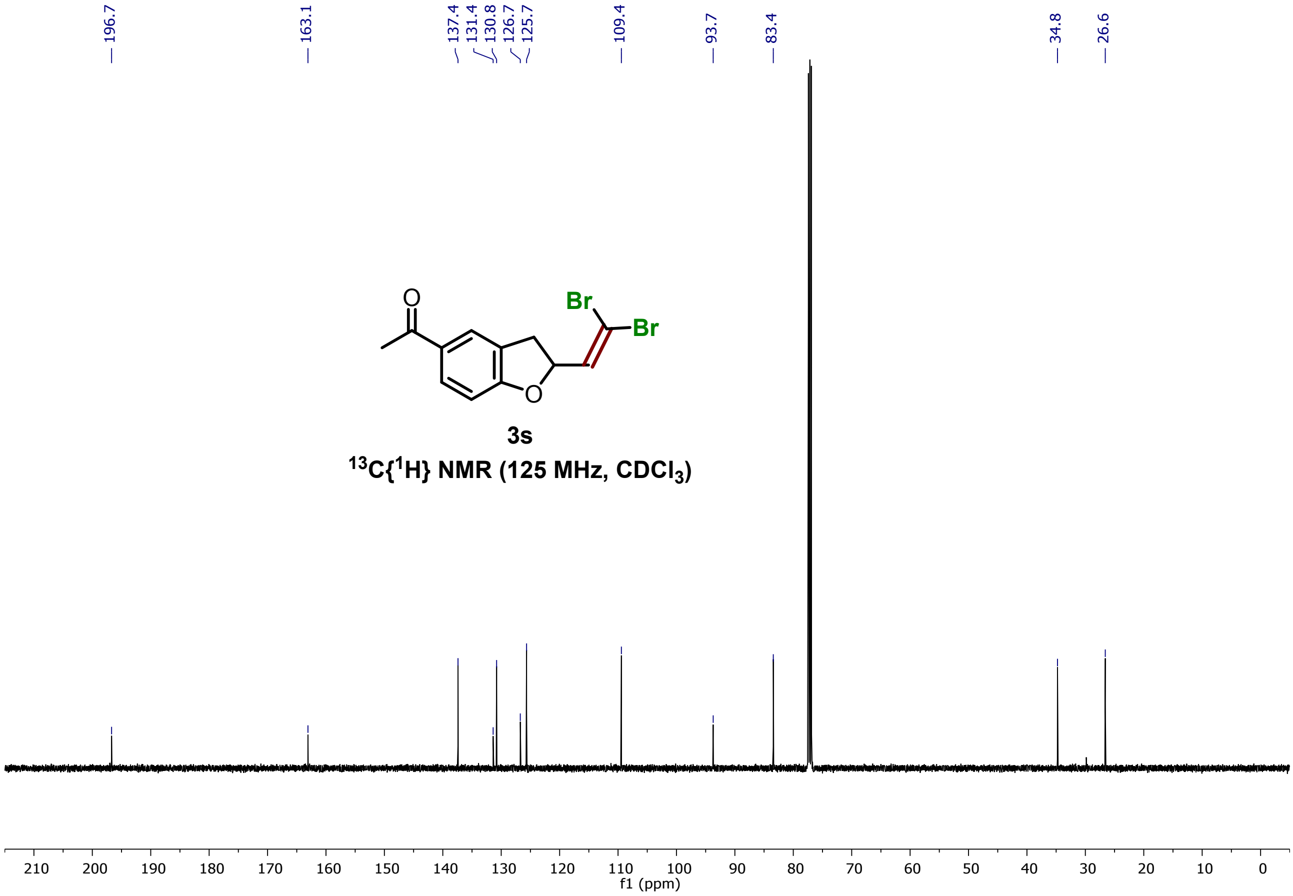

3t - ^{1}H NMR (CDCl$_3$).

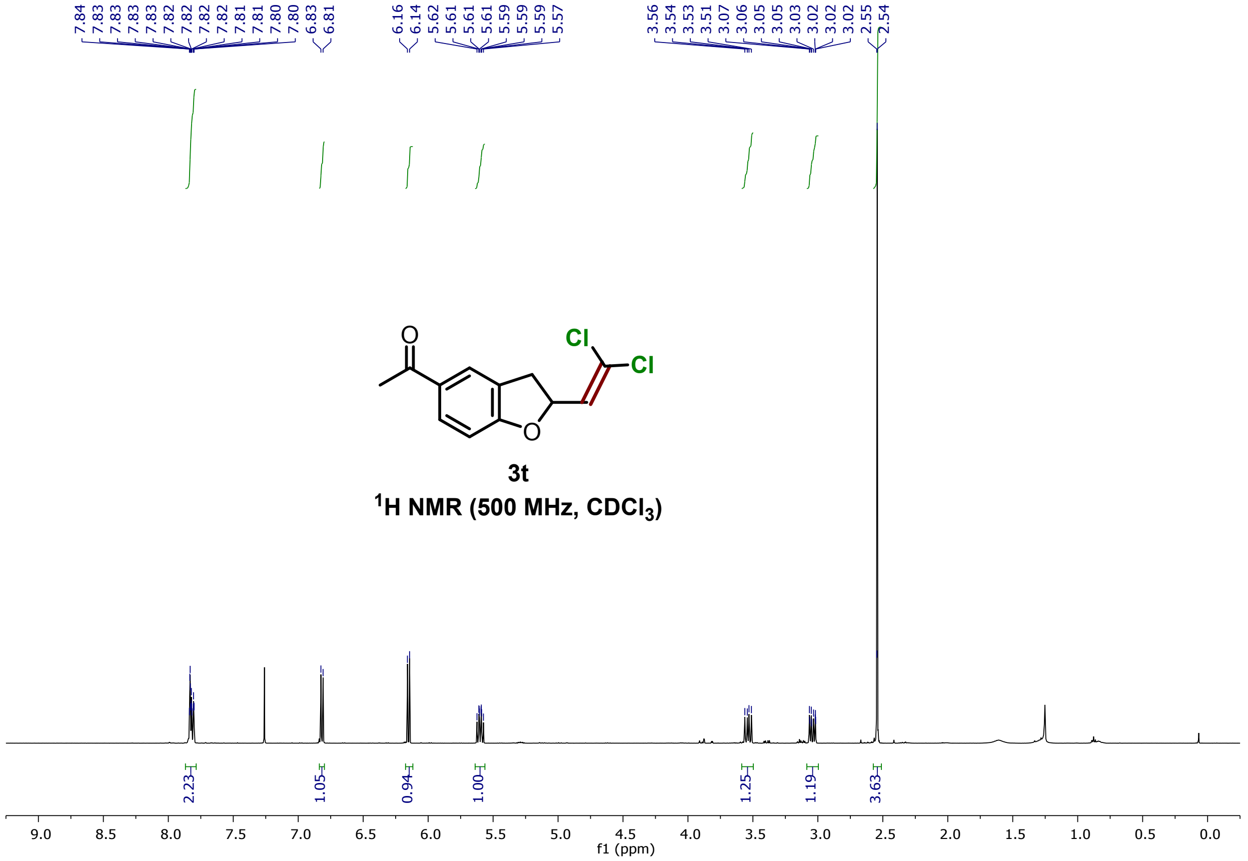

3t - ^{13}C{^{1}H} NMR (CDCl$_3$).

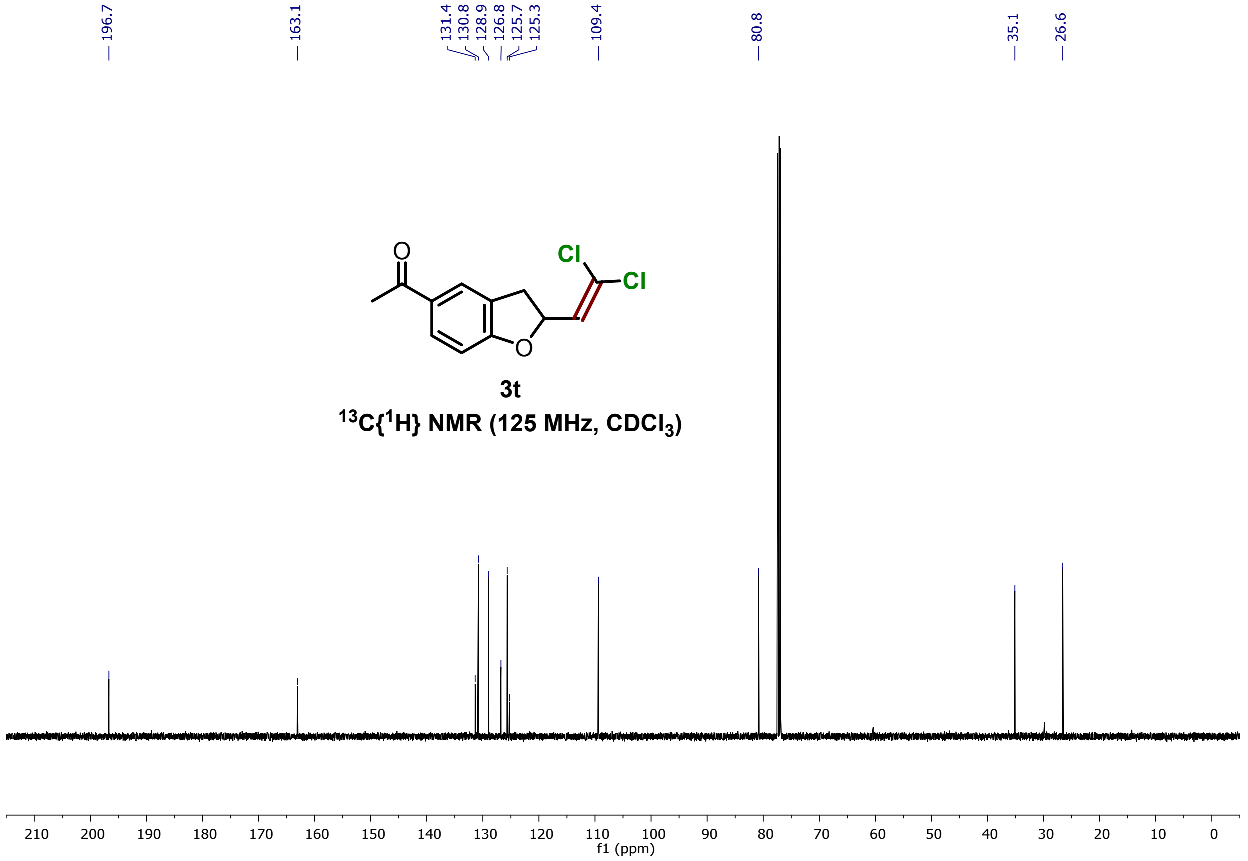

3aa - ^{1}H NMR (DMSO-d$_6$).

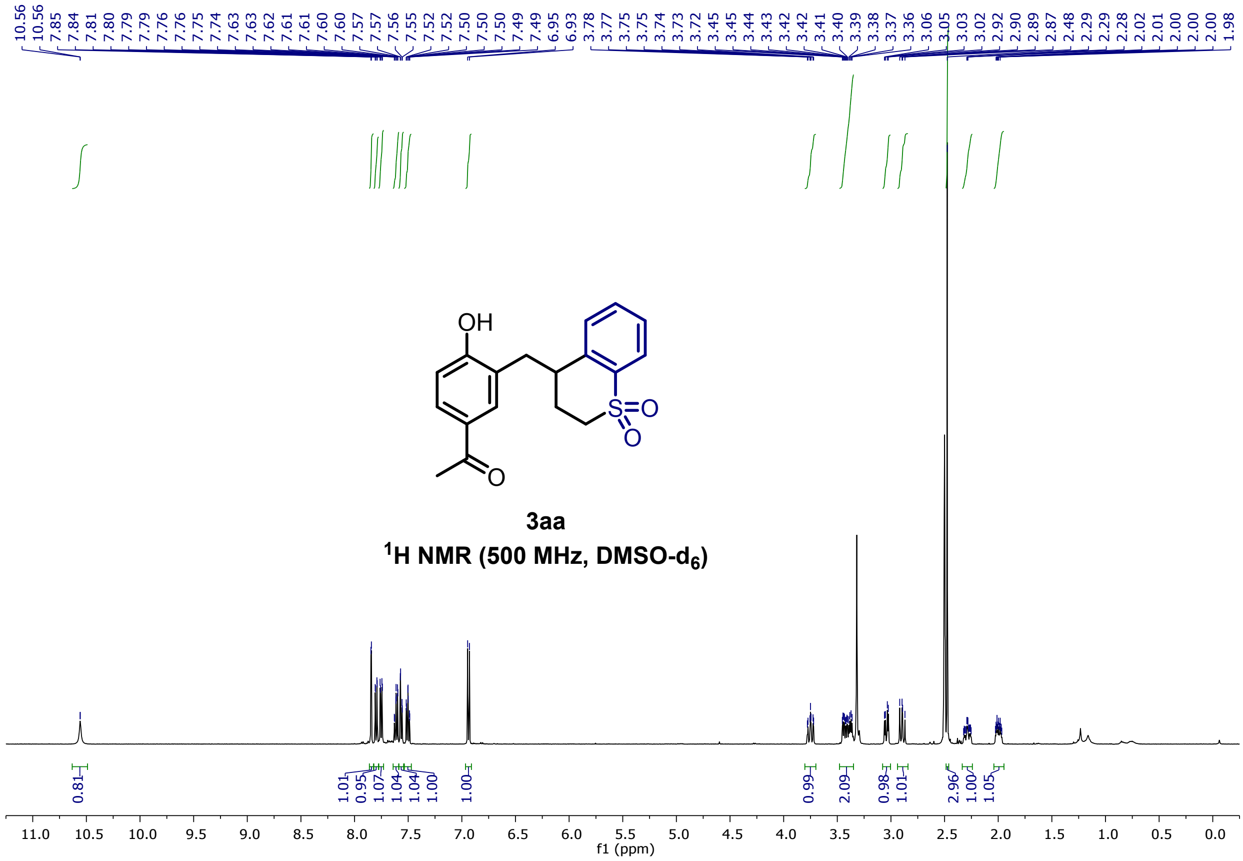

3aa - ^{13}C{^{1}H} NMR (DMSO-d$_6$).

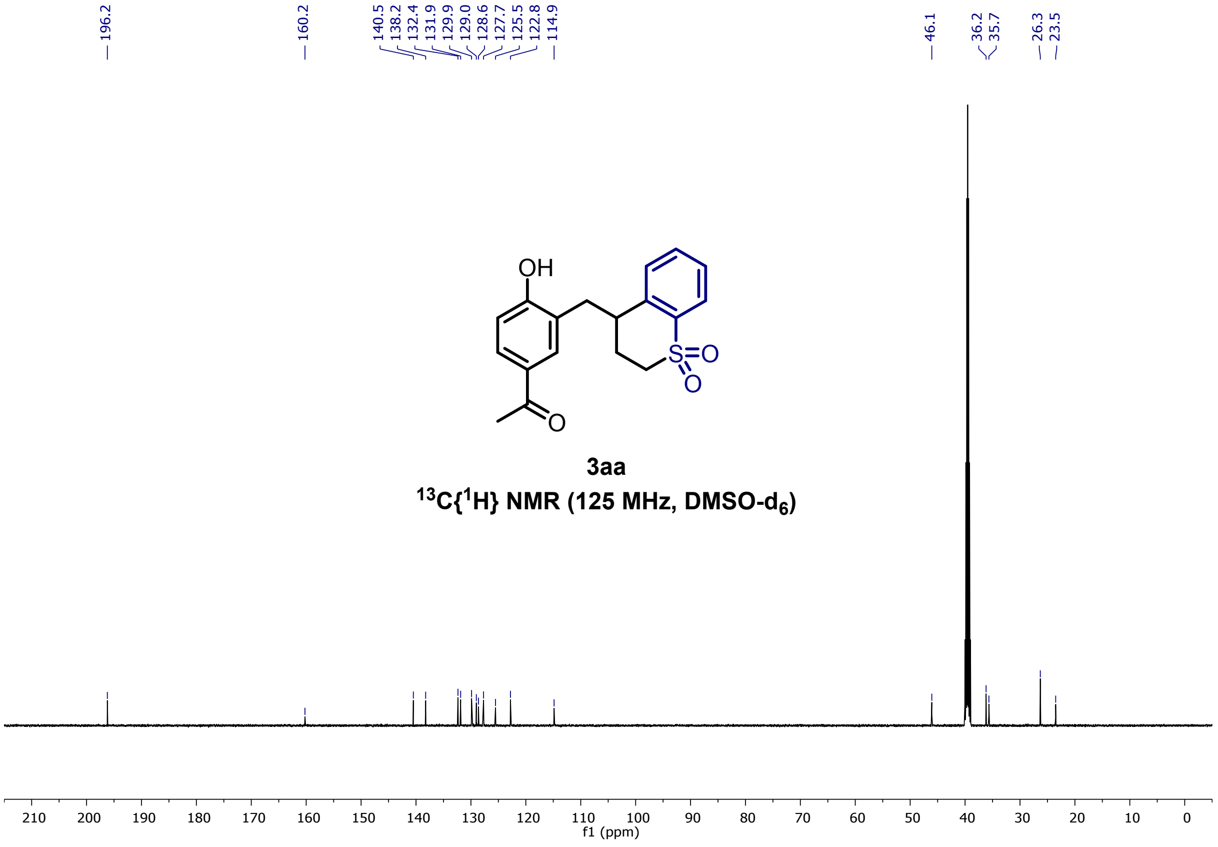

4a - ^{1}H NMR ($CDCl_3$).

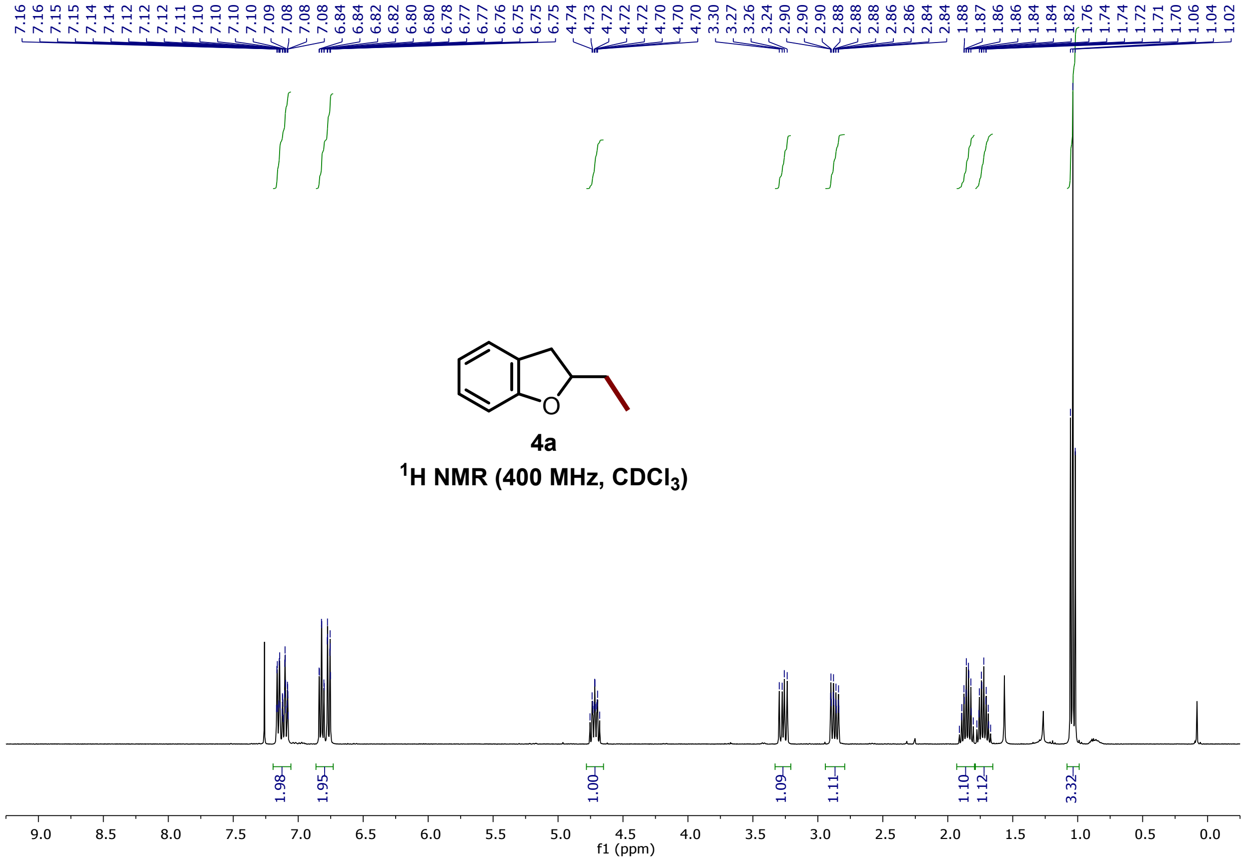

4a - $^{13}C\{^{1}H\}$ NMR ($CDCl_3$).

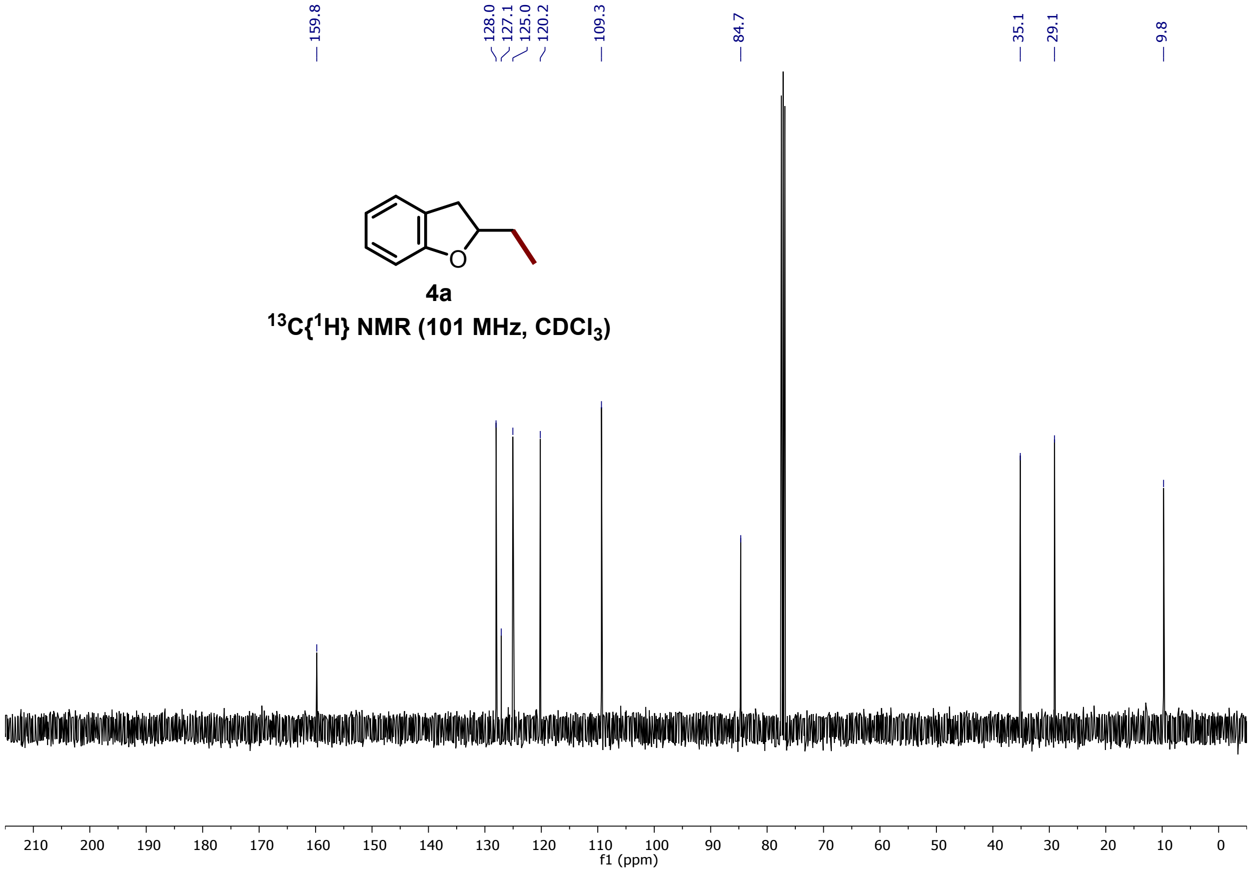

4b - ^{1}H NMR ($CDCl_3$).

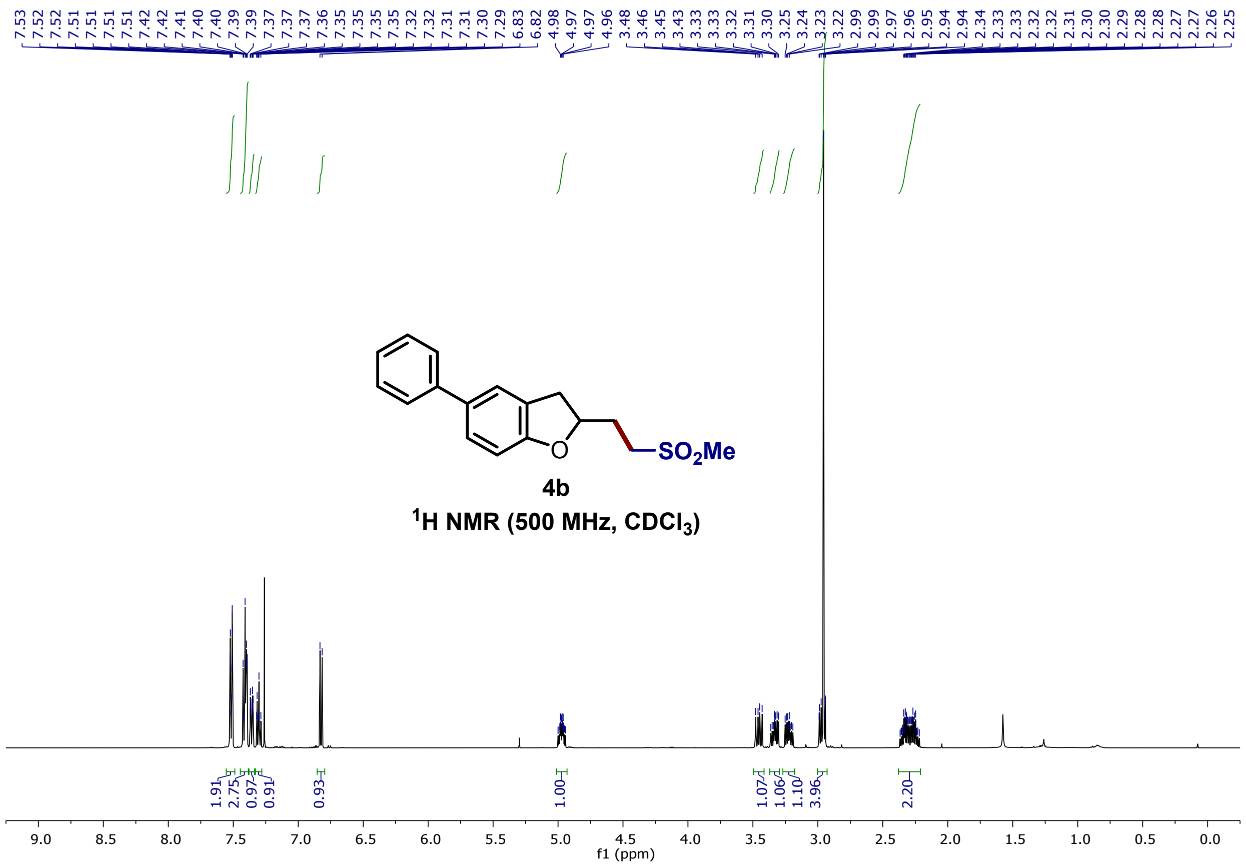

4b - ^{13}C{^{1}H} NMR ($CDCl_3$).

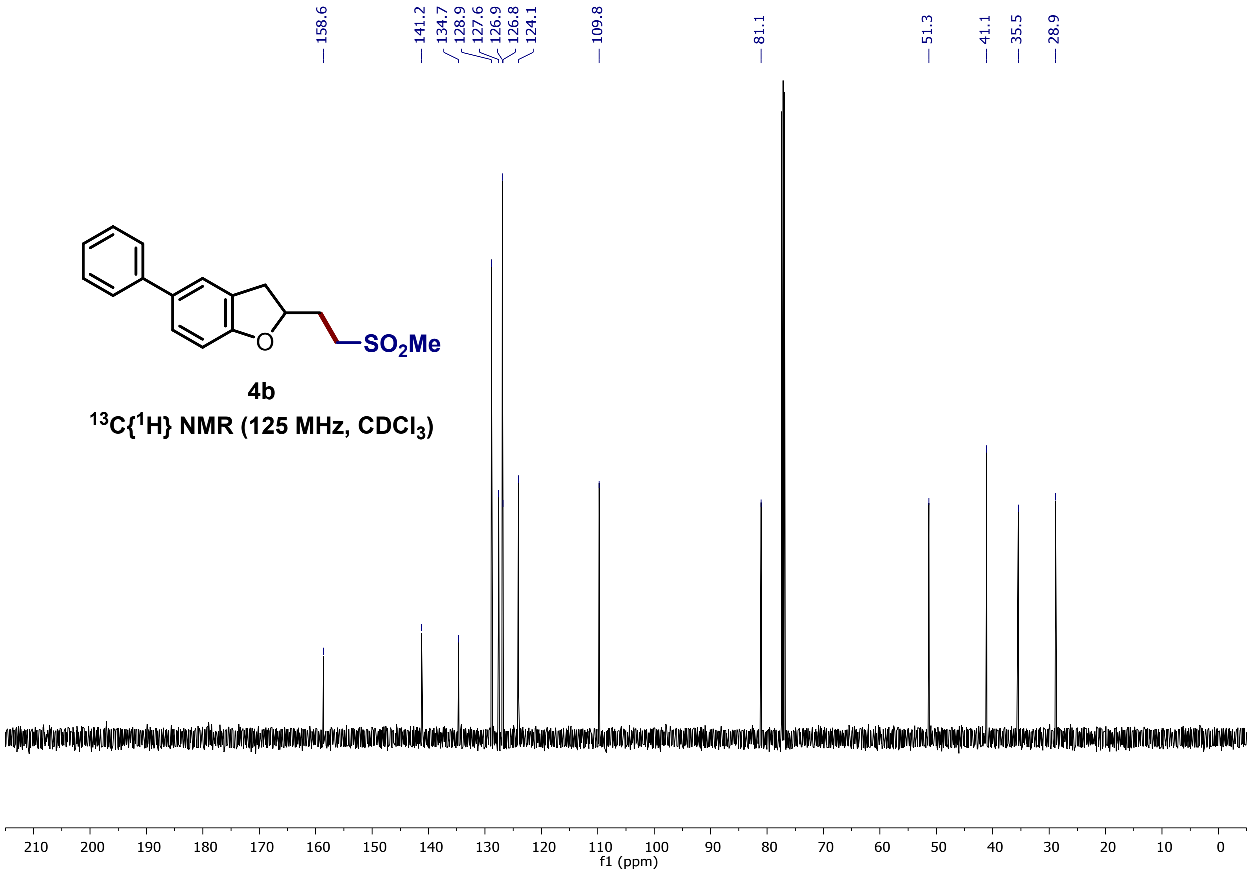

4c - ^{1}H NMR (CDCl$_3$).

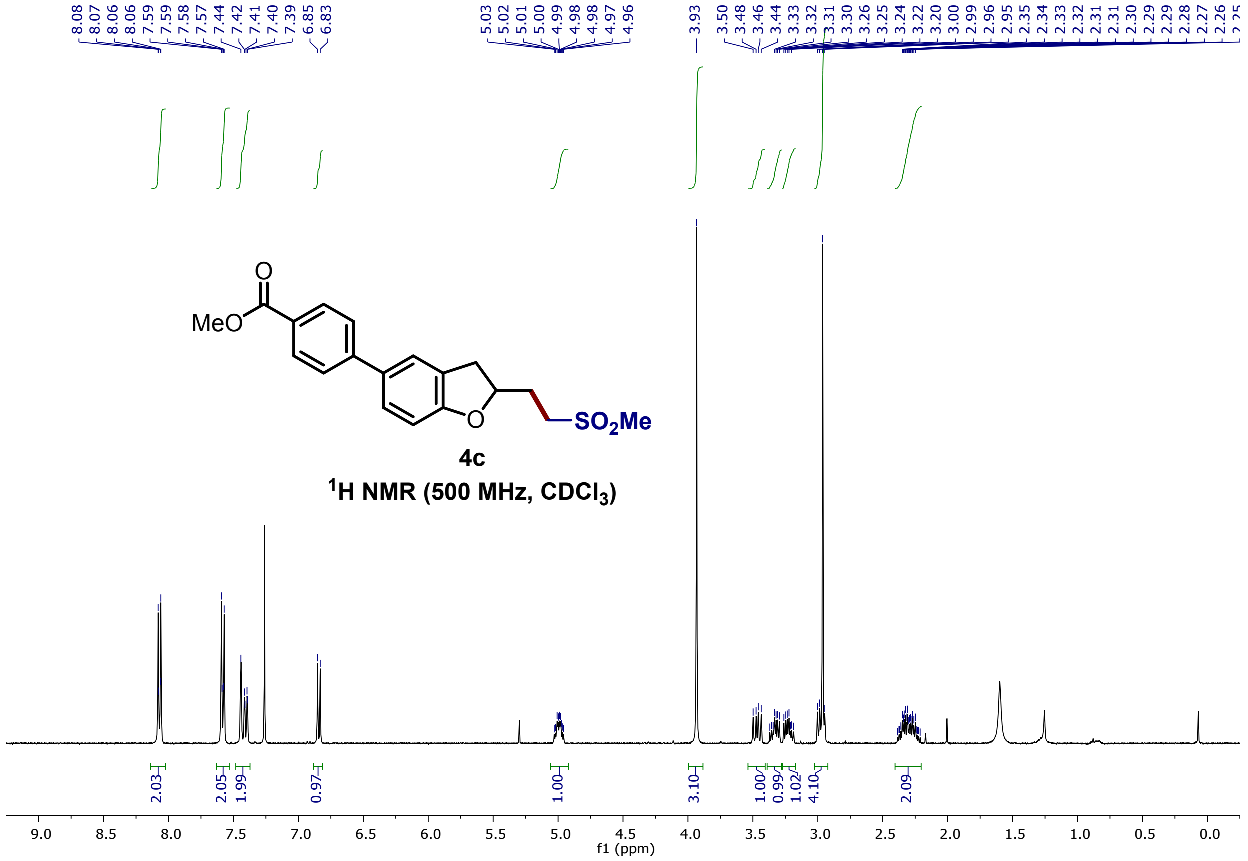

4c - ^{13}C{^{1}H} NMR (CDCl$_3$).

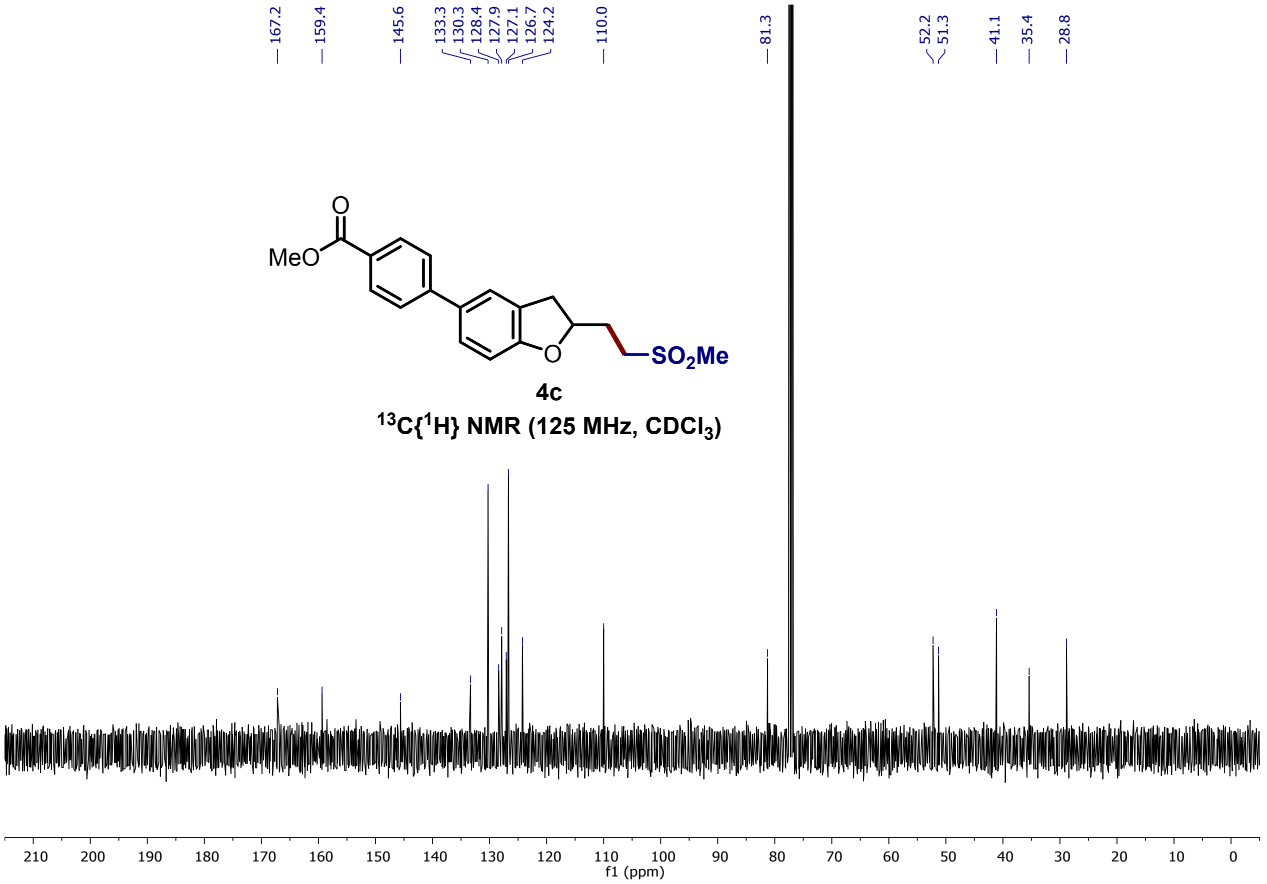

4d - ^{1}H NMR ($CDCl_3$).

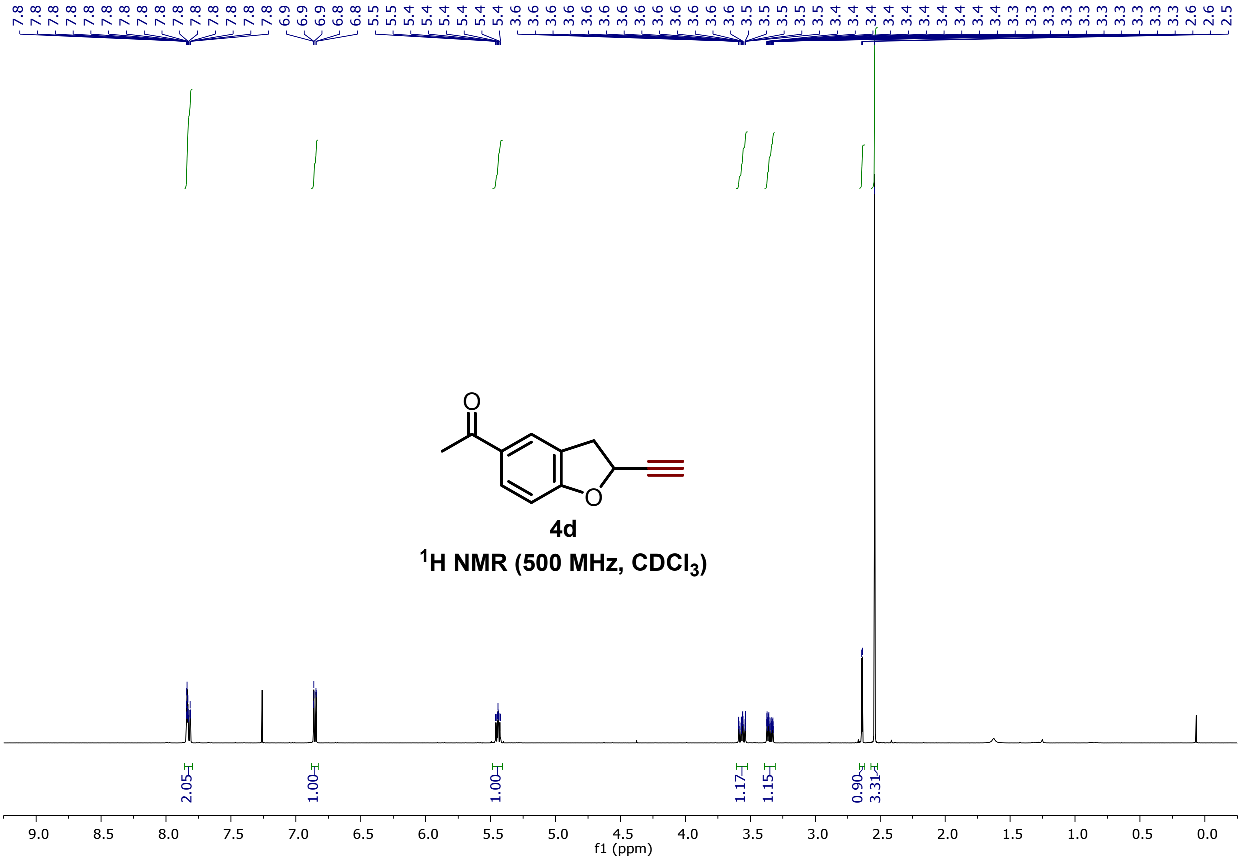

4d - ^{13}C{^{1}H} NMR ($CDCl_3$).

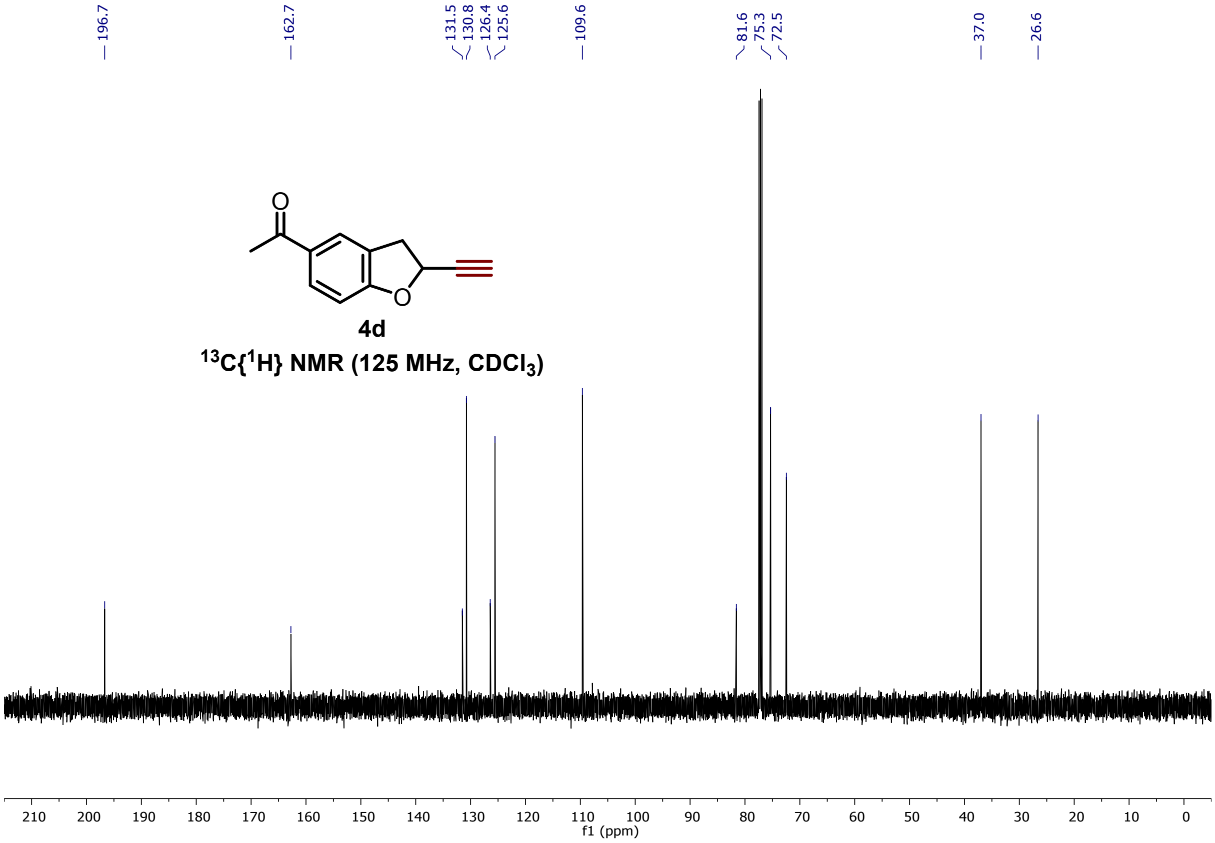

Supplement: Supplementary file 1 — jo3c00347_si_001.pdf [file jo3c00347_si_001.pdf]
